# Supplementary material for: Decadal sink-source shifts of forest aboveground carbon since 1988
Source: Nat Commun. 2026 Jul 30;17:7600. doi: 10.1038/s41467-026-76093-3 (PMC13424356; doi:10.1038/s41467-026-76093-3)
Supplement: Supplementary file 1 — Supplementary Information [file 41467_2026_76093_MOESM1_ESM.pdf]

Supplementary Figures

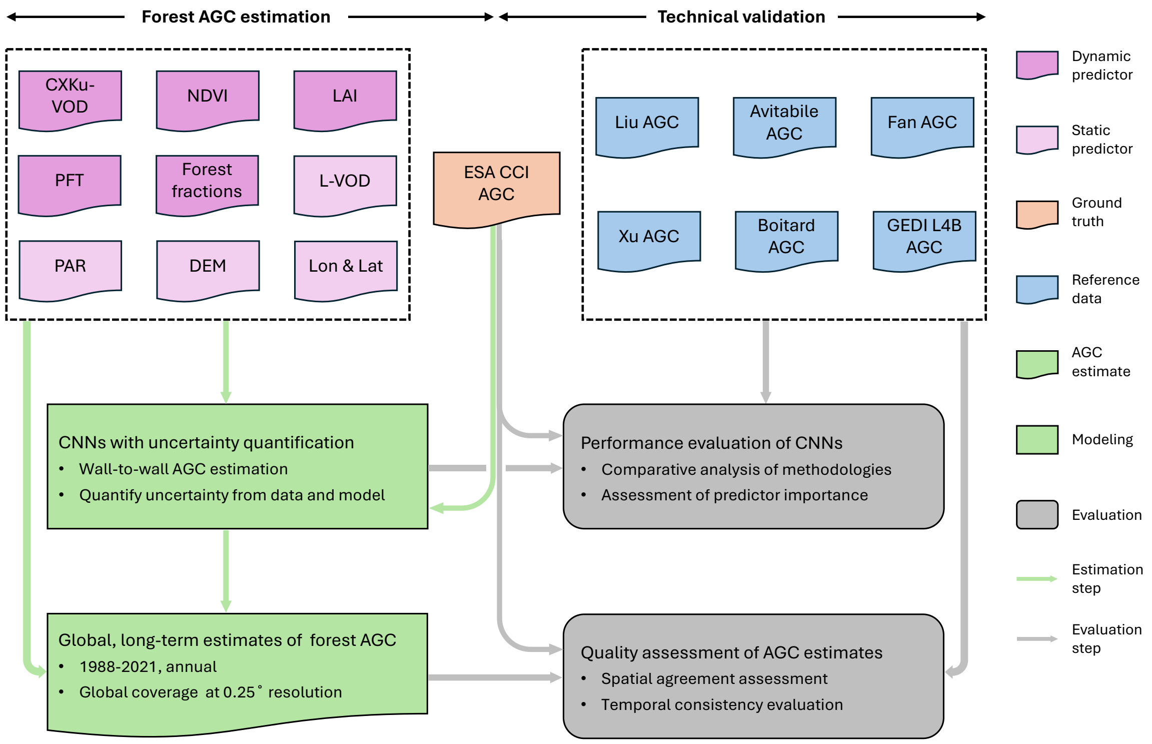

Supplementary Figure 1 Workflow for forest AGC estimation and technical validation. The framework consists of two main components: forest AGC estimation (left) and technical validation (right). The AGC estimation process integrates multi-source predictors (dynamic and static; see Table. 1) with a probabilistic convolutional neural network (CNN) ensemble approach to generate annual global AGC estimates from 1988 to 2021 at 0.25° resolution. Technical validation (right) assesses the accuracy and reliability of AGC estimates using multiple reference datasets, including ESA CCI AGC and other independent, shorter-term AGC products for evaluation. Evaluation steps include comparative analysis of methodologies, assessment of predictor importance, and quality evaluation of AGC estimates in terms of spatial agreement and temporal consistency.

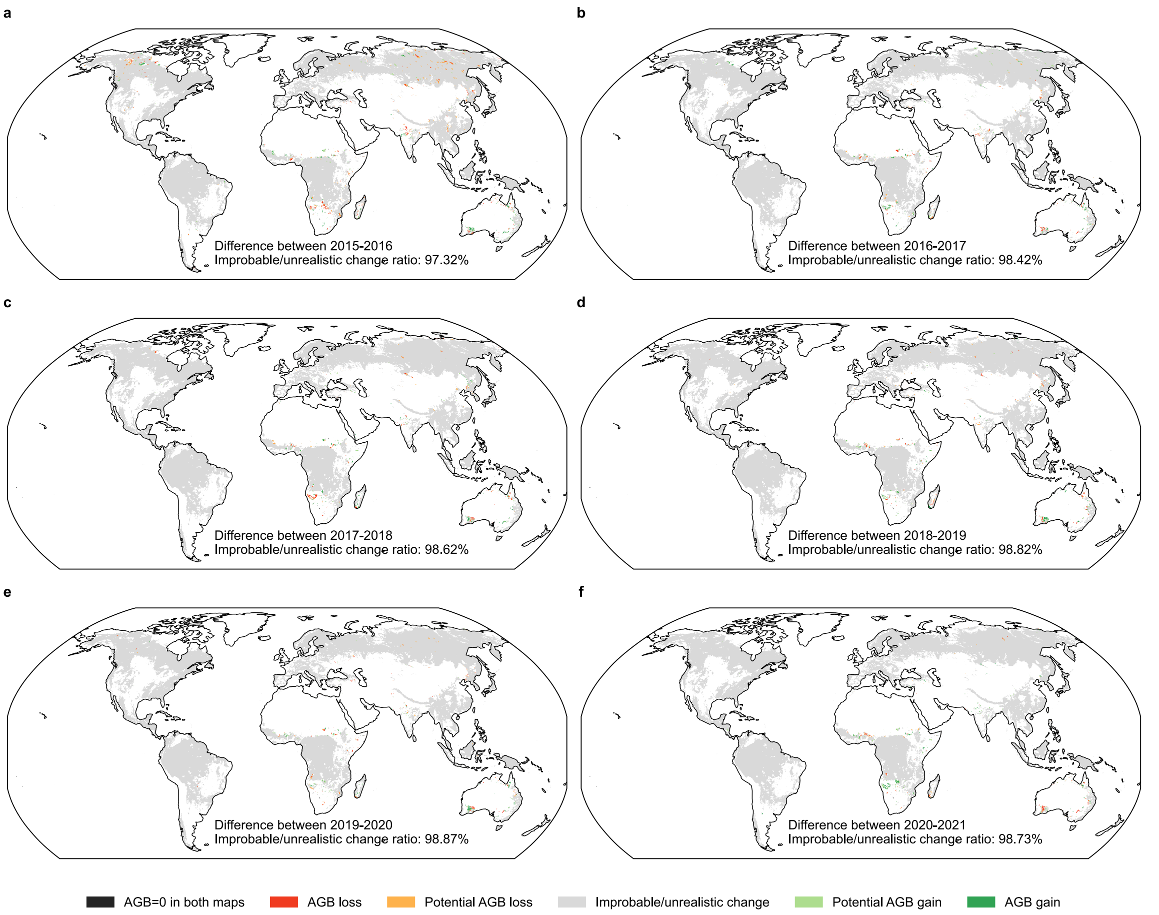

Supplementary Figure 2 Improbable or unrealistic changes in ESA CCI AGB annual differences. a–f, Global maps of annual AGB differences from ESA CCI for six consecutive years: 2015–2016 (**a**), 2016–2017 (**b**), 2017–2018 (**c**), 2018–2019 (**d**), 2019–2020 (**e**), and 2020–2021 (**f**). Across all years, over 95% of forested regions exhibit changes flagged as improbable or unrealistic, determined by the ESA CCI AGB user product and data quality flag, indicating substantial limitations in the reliability of interannual ESA CCI AGB estimates.

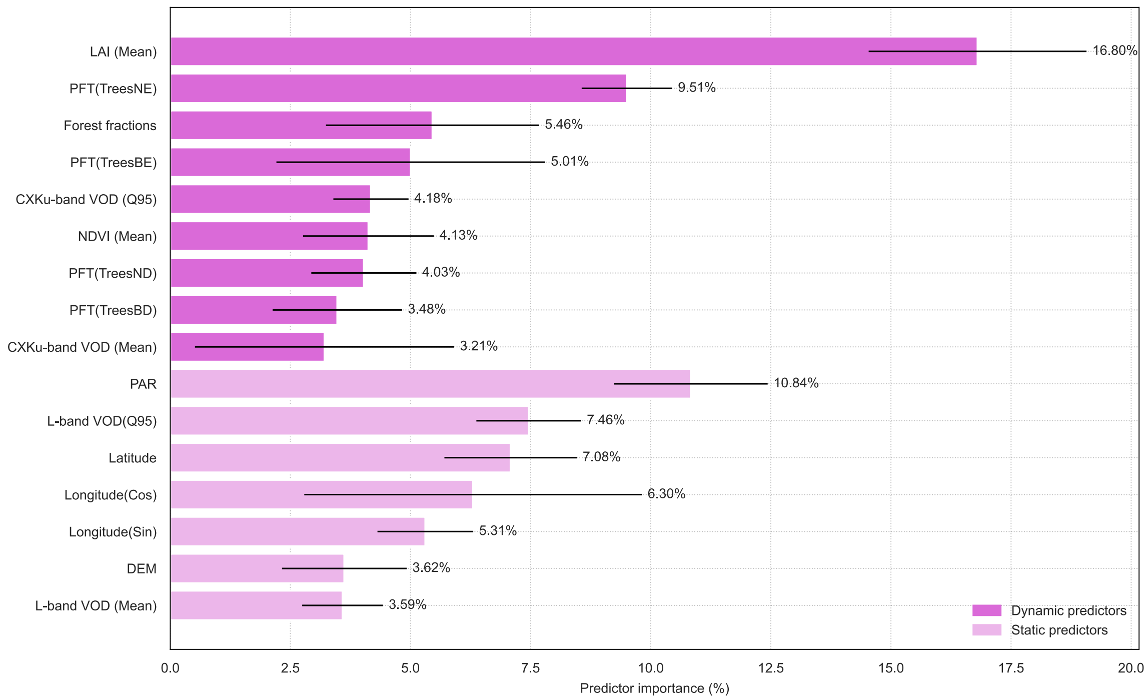

Supplementary Figure 3 Predictor importance in CNN-based AGC estimation. The relative importance of dynamic and static predictors in the final AGC estimates, computed using an explainable artificial intelligence technique (integrated gradients).

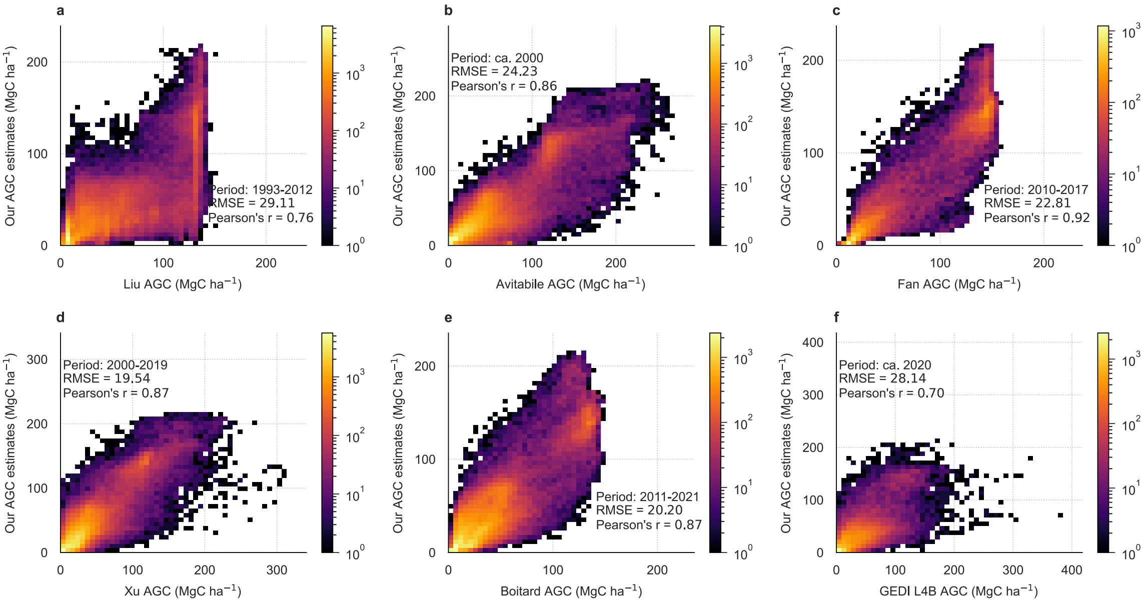

Supplementary Figure 4 Spatial consistency assessment between our AGC reconstruction and reference datasets. **a**–**f**, Hexbin scatter plots comparing the temporal mean of our reconstructed AGC against various reference AGC products. To ensure a fair spatial comparison, both our dataset and each reference product are temporally averaged over their specific overlapping time periods prior to calculating grid-cell-wise correlations: **a**, Liu et al. (1993–2012) [1], **b**, Avitabile et al. (ca. 2000) [2], **c**, Fan et al. (2010–2017) [3], **d**, Xu et al. (2000–2019) [4], **e**, Boitard et al. (2011–2021) [5], and **f**, GEDI L4B (ca. 2020) [6]. Each panel reports the root mean square error (RMSE) and the spatial Pearson correlation coefficient (r) computed across all valid grid cells. Overall, our AGC estimates exhibit strong spatial correlations with independent reference datasets (r ranging from 0.70 to 0.92), demonstrating the reliability of our reconstructed spatial patterns. All Pearson's r values reported here are statistically significant with $p < 0.001$.

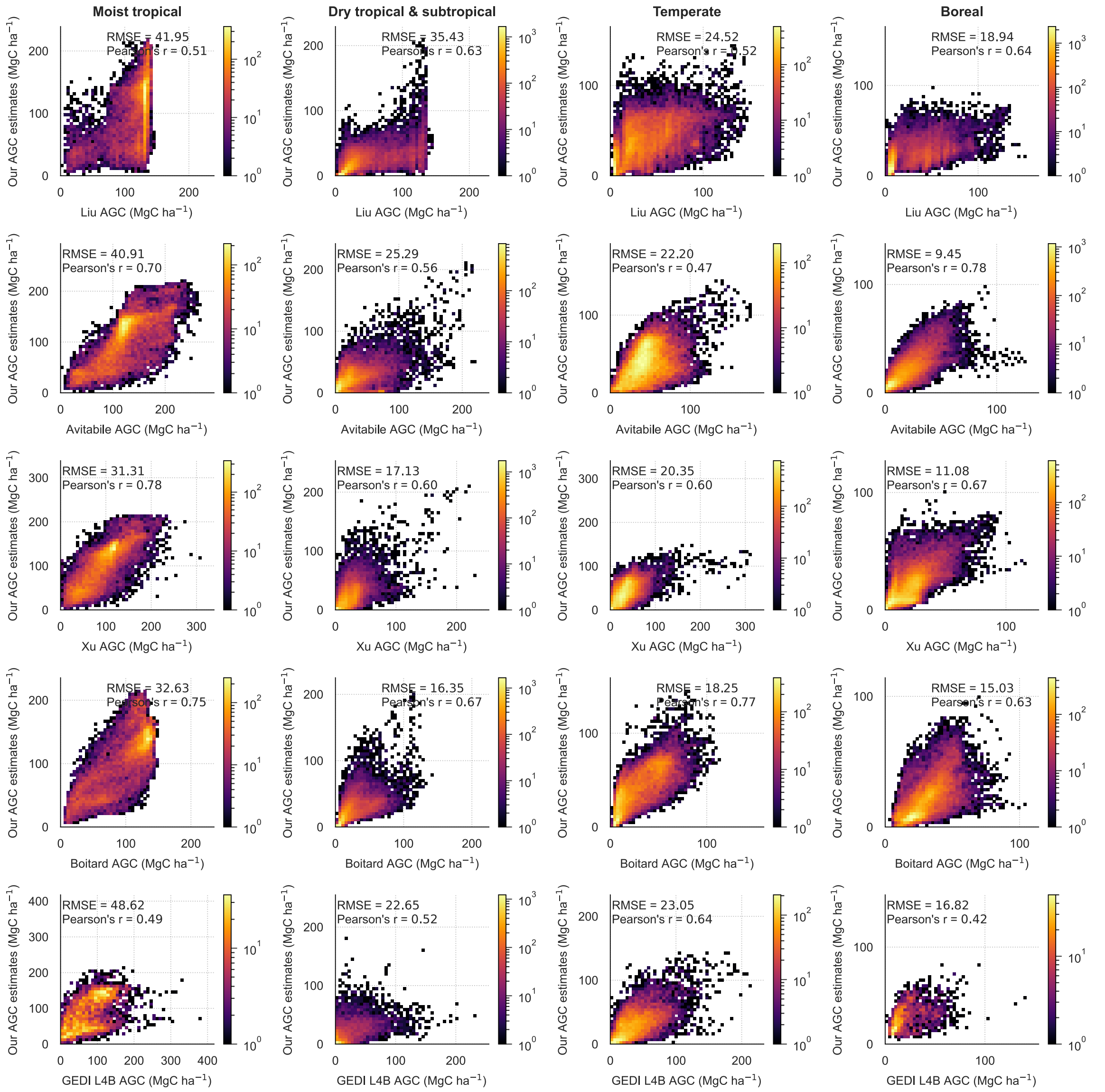

Supplementary Figure 5 Biome-scale spatial consistency between our AGC estimates and reference datasets. Hexbin scatter plots comparing the temporal mean of our AGC estimates with reference AGC products across four distinct biomes: moist tropical forests (first column), dry tropical & subtropical forests (second column), temperate forests (third column), and boreal forests (fourth column). Each row corresponds to a different reference dataset: Liu et al. [1], Avitabile et al. [2], Xu et al. [4], Boitard et al. [5], and GEDI L4B [6]. The root mean square error (RMSE) and Pearson correlation coefficient (r) are reported for each comparison. Although spatial correlations naturally decrease when evaluated within localized regions, the spatial consistency remains robust. The maximum spatial correlations achieved are $r = 0.78$ in moist tropical forests, $r = 0.67$ in dry tropical & subtropical forests, $r = 0.77$ in temperate forests, and $r = 0.78$ in boreal forests. Lower correlations are observed with the Liu et al. [1] and GEDI L4B [6] datasets, likely due to biases from high-frequency Ku-band VOD and empirical methods in the Liu dataset, as well as the sparse distribution of GEDI sampling, which may introduce bias when aggregated to the 0.25° grid. All Pearson's r values reported here are statistically significant with $p < 0.001$.

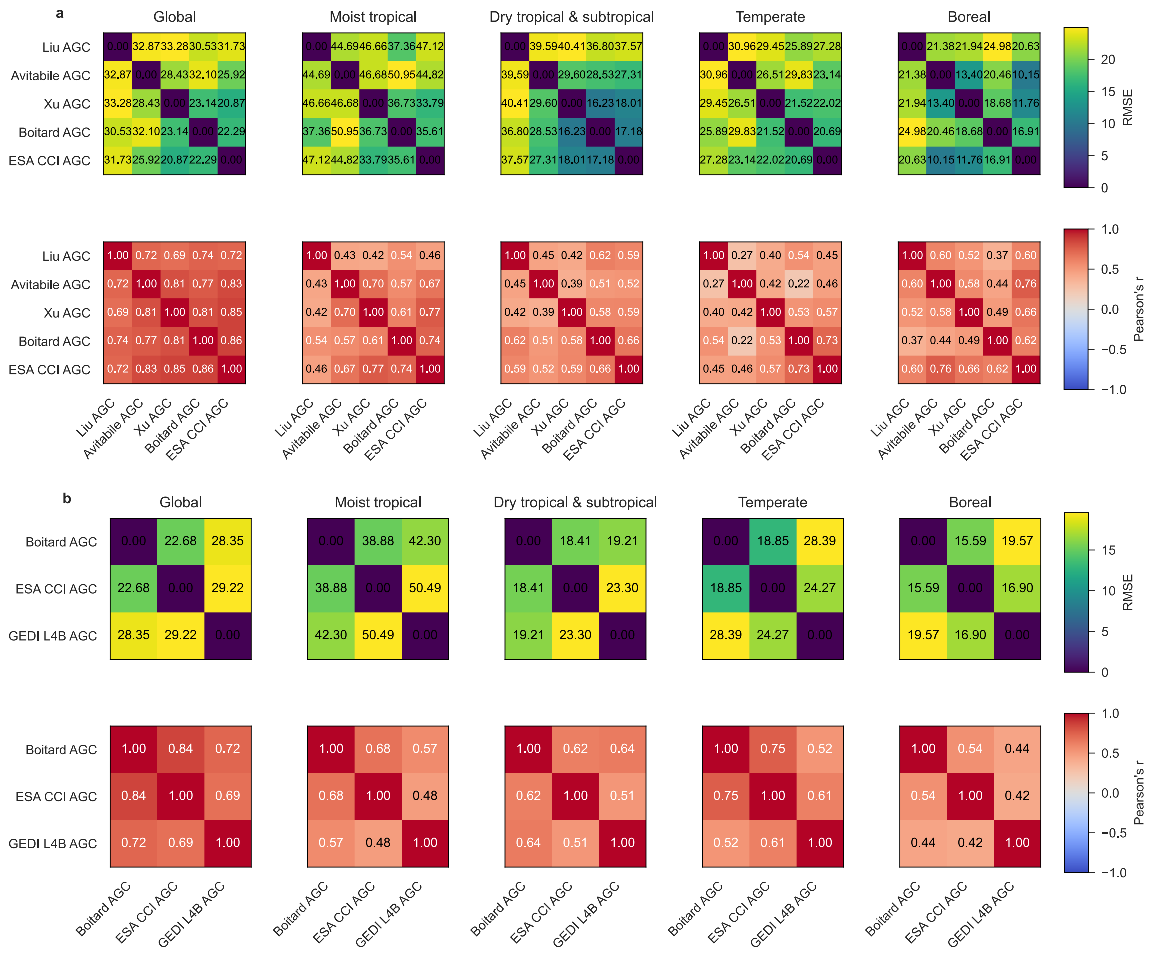

Supplementary Figure 6 Intercomparison of AGC reference datasets at global and biome scales. a, Pairwise comparisons of AGC reference datasets (Liu, Avitabile, Xu, Boitard, and ESA CCI) in terms of root mean square error (RMSE, top row) and Pearson correlation coefficient (r, bottom row) at global and biome scales (moist tropical, dry tropical & subtropical, temperate, and boreal forests). **b**, Pairwise comparisons of Boitard AGC, ESA CCI AGC, and GEDI L4B AGC, including RMSE (top row) and Pearson's r (bottom row). Correlations generally decrease from the global scale to individual biomes, highlighting inconsistencies in data acquisition and processing methods across references. Lower correlations in biome-level comparisons suggest that regional discrepancies in methodology and sensor coverage contribute to spatial inconsistencies among AGC reference datasets. All Pearson's correlation coefficients (r) reported here are statistically significant with $p < 0.001$.

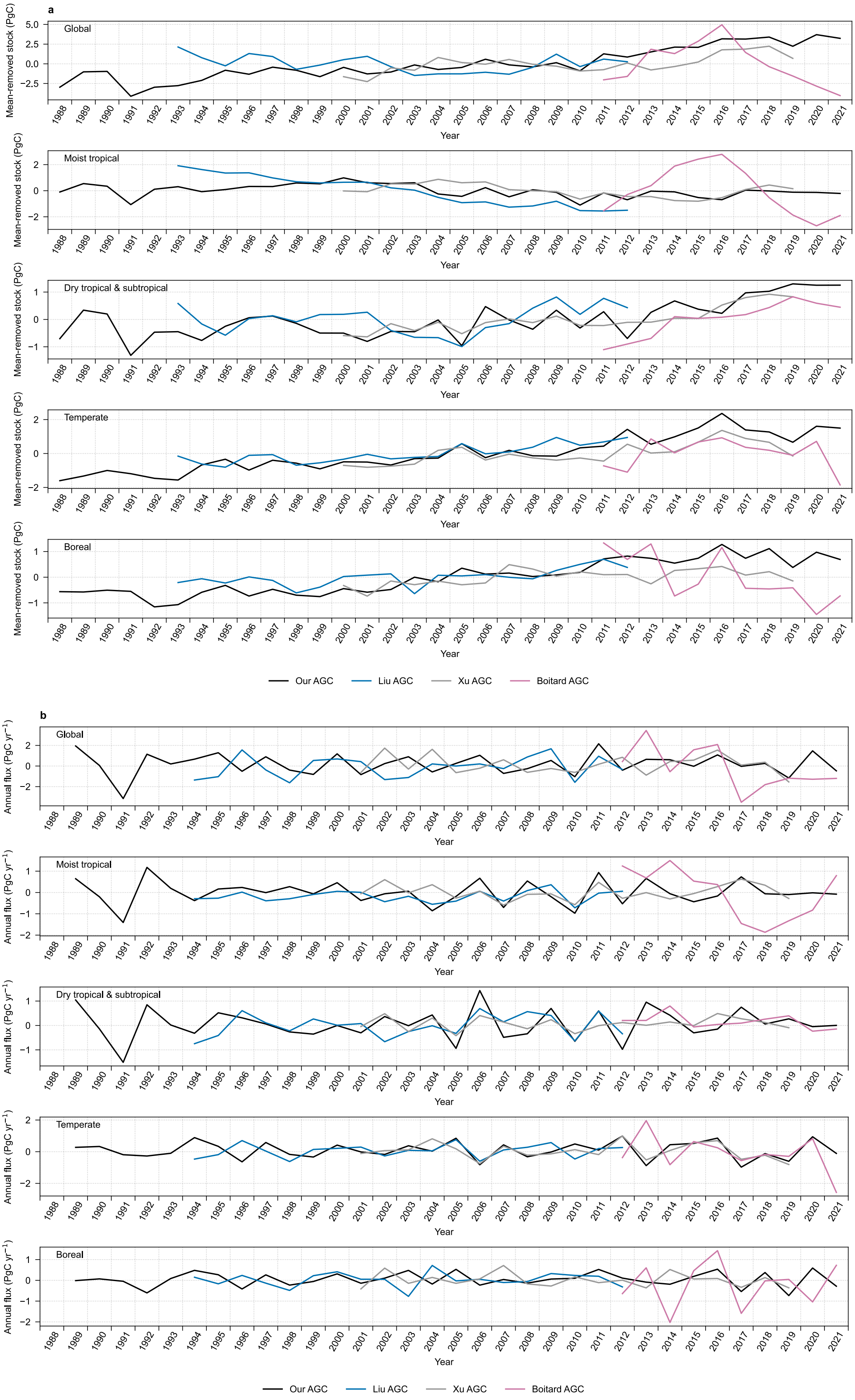

Supplementary Figure 7 Temporal agreement between our AGC estimates and reference datasets at global and biome scales. **a**, Time series of AGC stock from our estimates (black) compared with Liu et al. (blue) [1], Xu et al. (gray) [4], and Boitard et al. (pink) [5] at global and biome levels. **b**, Annual AGC flux comparisons, revealing generally strong agreement across biomes.

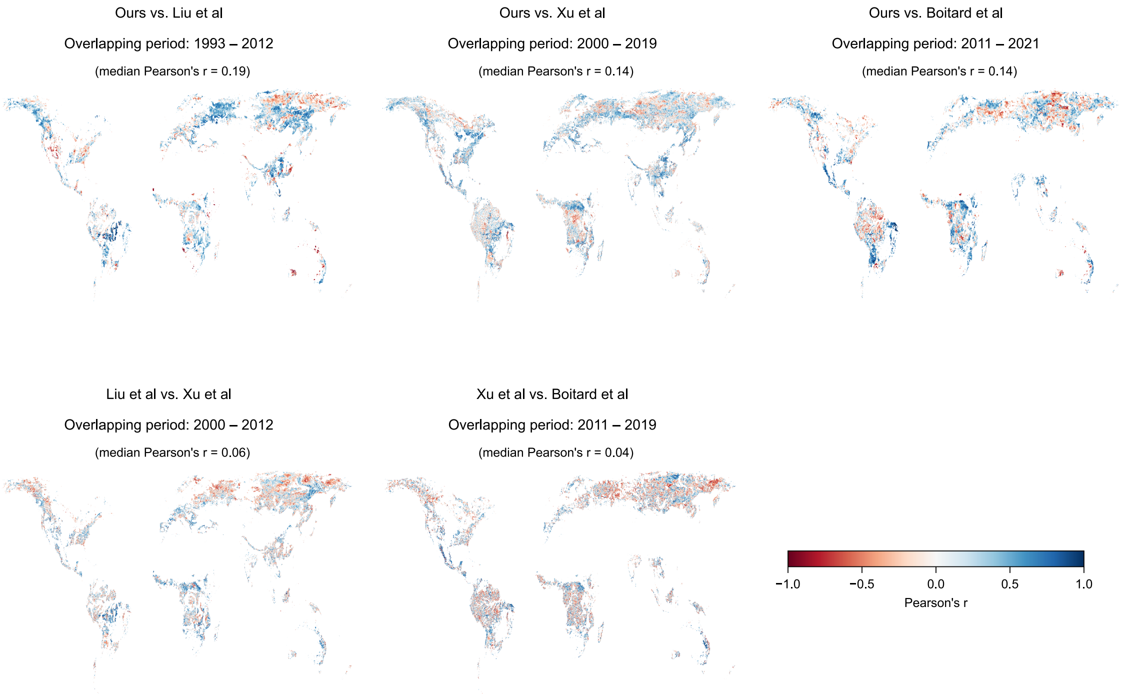

Supplementary Figure 8 Grid-cell level temporal correlation maps between AGC datasets. Spatial distribution of Pearson's r comparing annual AGC density time series across overlapping periods for different dataset pairs: our estimates versus Liu et al. [1], Xu et al. [4], and Boitard et al. [5], as well as comparisons among the reference datasets themselves. Each map shows the pairwise correlation computed at each grid cell, highlighting areas of stronger (blue) or weaker (red) agreement. Median correlation values are reported for each comparison.

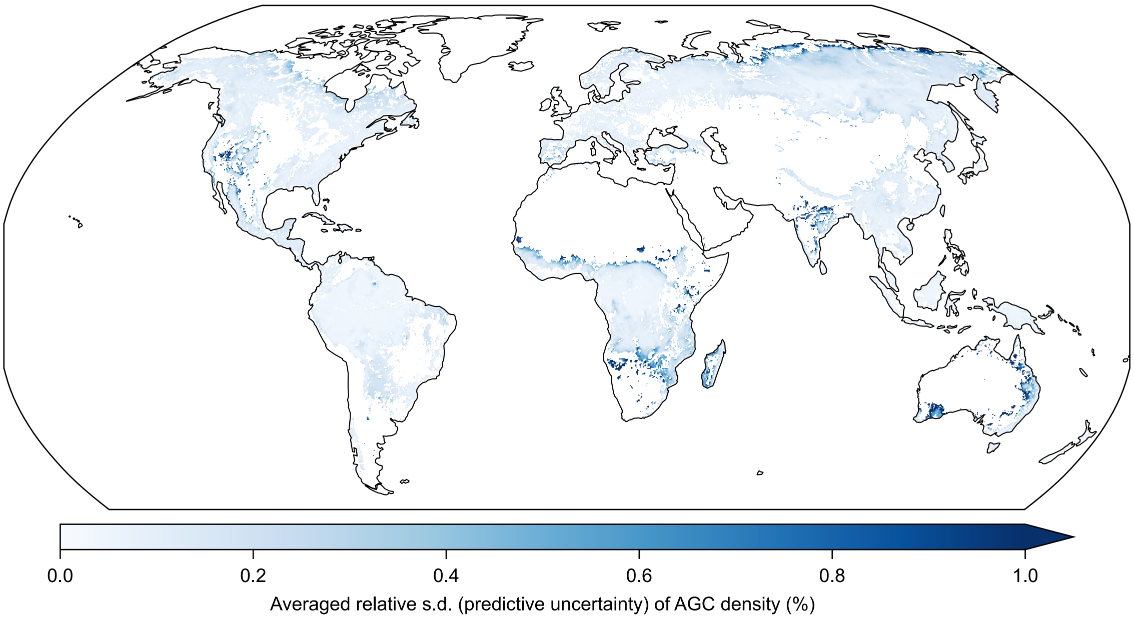

Supplementary Figure 9 Multi-year averaged relative predictive uncertainty (standard deviation). The relative uncertainty is calculated as the ratio of the predictive standard deviation to the predicted mean AGC for each year, then averaged over time. Higher relative uncertainty values (darker blue) indicate areas where AGC estimates have higher uncertainty relative to their mean values. Notably, while absolute predictive uncertainty is typically higher in high-biomass regions (e.g., dense tropical forests), relative uncertainty is elevated in low-biomass areas, where small absolute errors in estimation result in large relative variations.

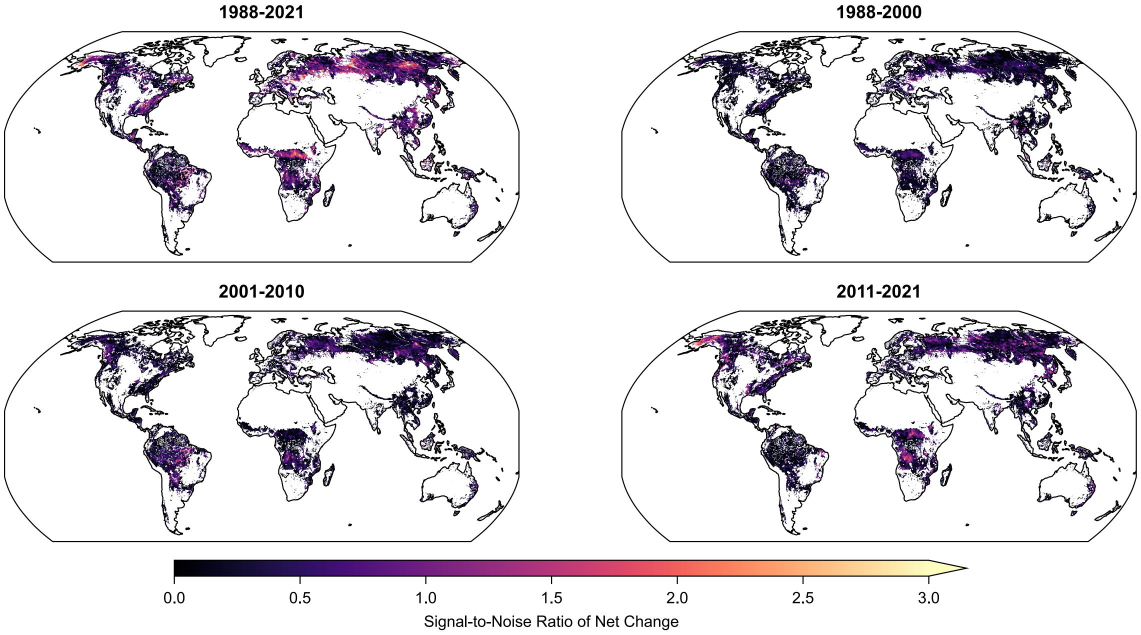

Supplementary Figure 10 Grid-cell-wise Signal-to-Noise Ratio (SNR) of forest AGC net changes. Spatially explicit SNR maps of cumulative AGC changes for the overall study period (1988–2021) and three decadal intervals (1988–2000, 2001–2010, and 2011–2021). The SNR is calculated at the 0.25° grid-cell level using a Monte Carlo ensemble approach (based on 1,000 simulations drawn from our model's predictive probability distributions). It is defined as the ratio of the absolute ensemble mean net change to the ensemble standard deviation.

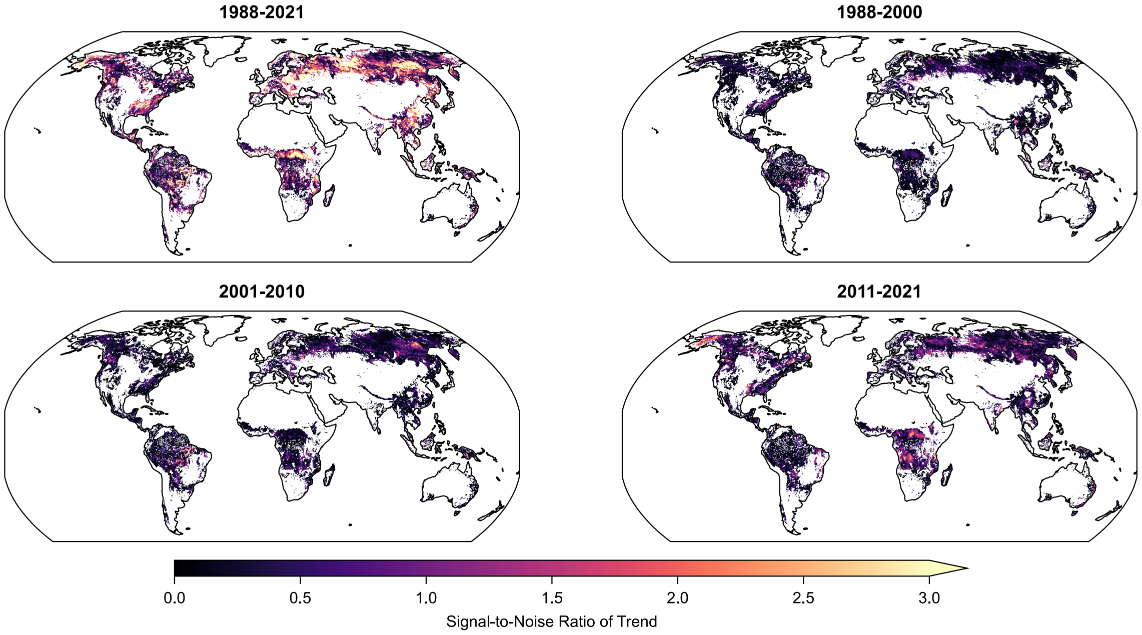

Supplementary Figure 11 Grid-cell-wise Signal-to-Noise Ratio (SNR) of forest AGC trends. Spatially explicit SNR maps of AGC trends for the overall study period (1988–2021) and three decadal intervals (1988–2000, 2001–2010, and 2011–2021). The trend SNR is calculated at the 0.25° grid-cell level utilizing a Monte Carlo ensemble approach (based on 1,000 simulations drawn from the model's predictive probability distributions). For each grid cell, the SNR is defined as the ratio of the absolute ensemble mean trend slope to the ensemble standard deviation of the slopes. Note that the years 1991 and 1992, which encompass the carbon flux anomalies caused by the Mt. Pinatubo eruption, are excluded from trend calculations.

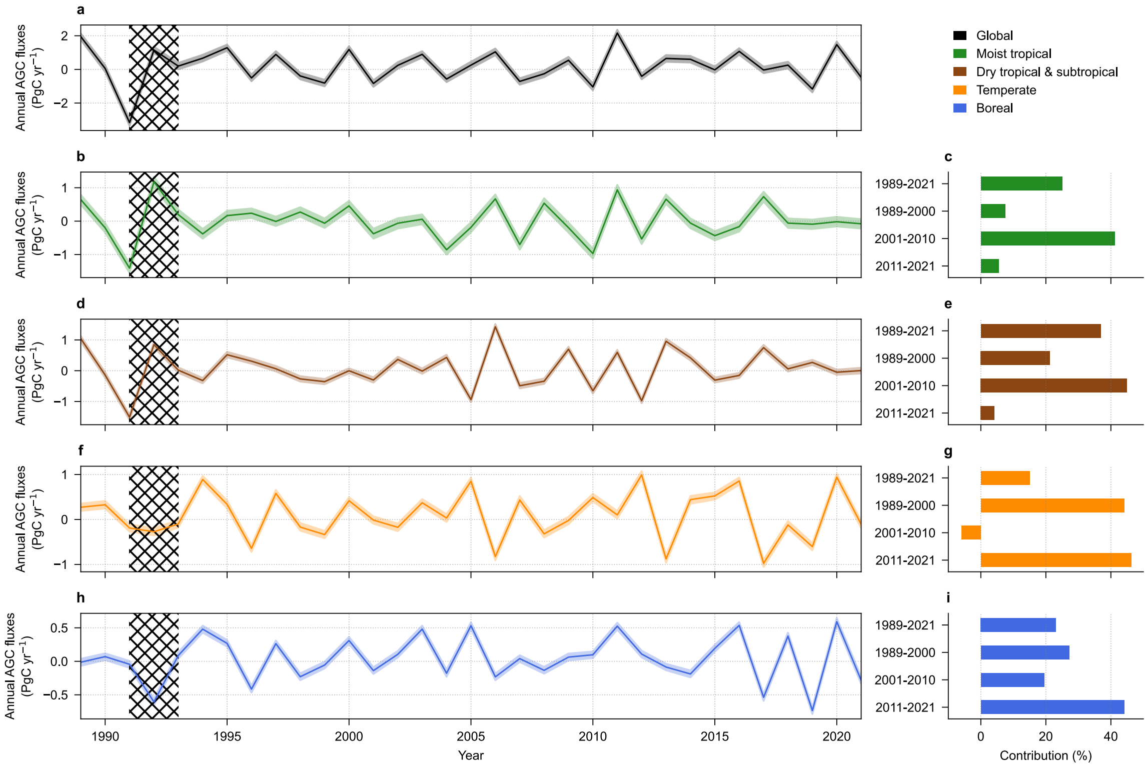

Supplementary Figure 12 Interannual variability of AGC flux for global and biome-specific regions. **a**, Global AGC flux interannual variability from 1989 to 2021. **b**, **d**, **f**, and **h**, AGC flux interannual variability for moist tropical, dry tropical & subtropical, temperate, and boreal biomes, respectively. **c**, **e**, **g**, and **i**, Contribution of each biome to the global AGC flux interannual variability for different periods (1989–2021, 1989–2000, 2001–2010, and 2011–2021). The calculation of contribution excludes the years 1991–1993 (the shaded cross-hatched region) due to unusual carbon flux anomalies caused by volcanic eruption. Shaded regions in each panel represent the 95% uncertainty interval (see Methods).

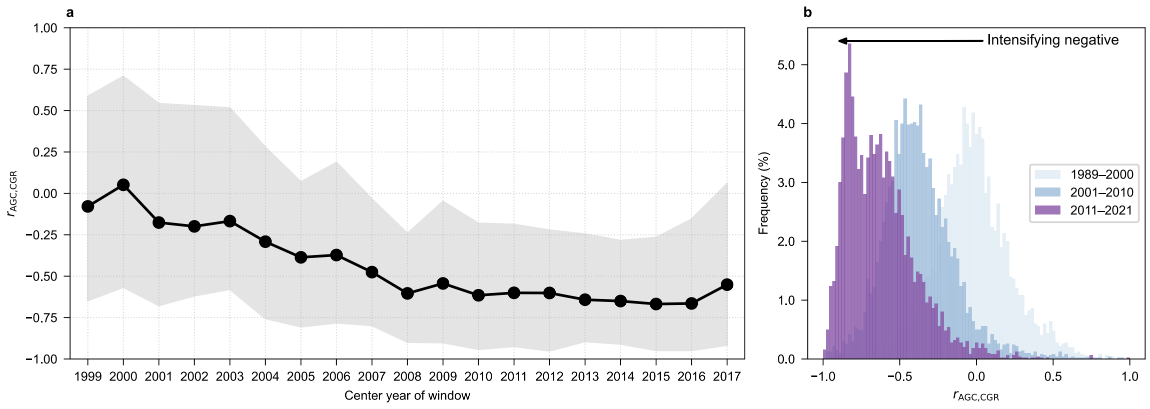

Supplementary Figure 13 Intensifying negative correlation between the interannual variability in tropical AGC fluxes and atmospheric CO_2 growth rates. a, Pearson's correlation coefficients (r) between detrended annual tropical aboveground carbon (AGC) fluxes and atmospheric CO_2 growth rates (CGR), calculated within 10-year moving windows from 1994 to 2017. The shaded area shows the 95% confidence interval estimated from 5000 bootstrap samples. The center year of each window ranges from 1994 to 2017, starting after the major volcanic eruptions in 1991–1993. **b**, Distributions of correlation coefficients derived from 5000 bootstrap resamples for the periods 1989–2000, 2001–2010, and 2011–2021. The years 1991–1993 are excluded due to major volcanic eruptions. A clear shift toward more negative correlations is observed over time, suggesting an intensifying negative AGC-CGR coupling in the tropics.

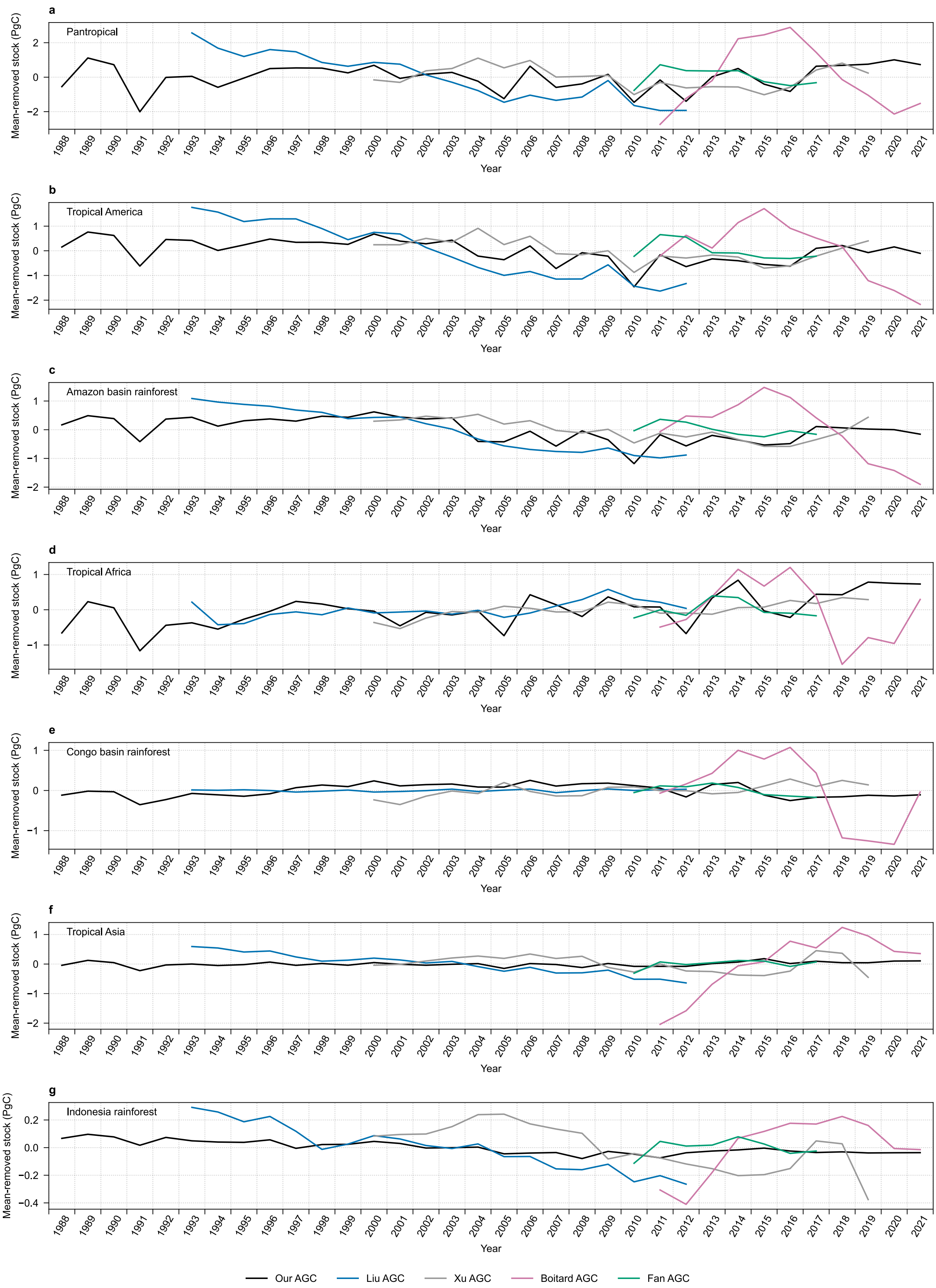

Supplementary Figure 14 AGC stock time series from four reference datasets in tropical regions. a–g, Time series of AGC stock from our study, Liu et al. [1], Xu et al. [4], Boitard et al. [5], and Fan et al. [3] across different tropical regions: pan-tropical (**a**), tropical America (**b**), Amazon Basin rainforest (**c**), tropical Africa (**d**), Congo Basin rainforest (**e**), tropical Asia (**f**), and Indonesian rainforest (**g**). We note that inconsistencies exist among these references. Fan AGC and Boitard AGC both rely on L-VOD and empirical methods but exhibit substantial differences in AGC dynamics. These discrepancies highlight the high uncertainty in AGC dynamics across tropical regions, making it challenging to integrate existing reference datasets for long-term AGC dynamics analysis.

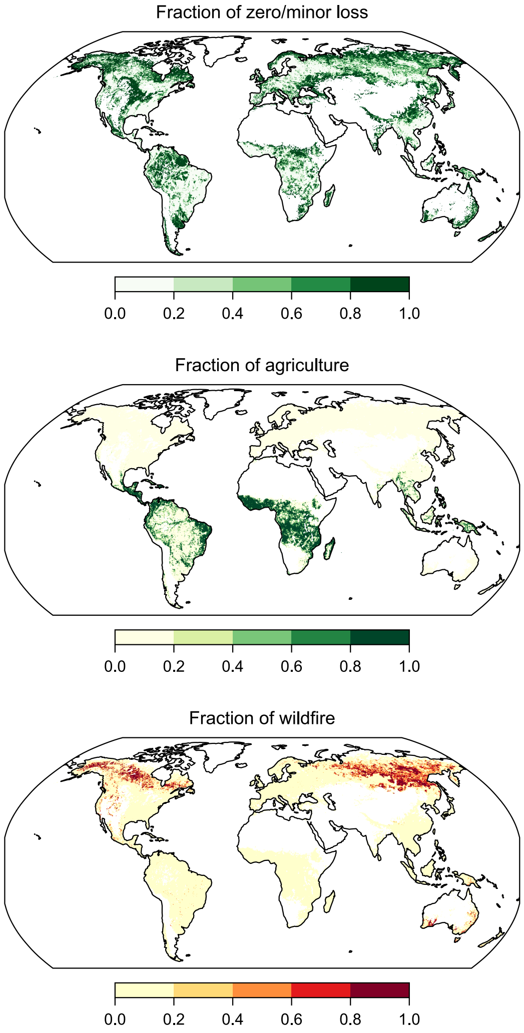

Supplementary Figure 15 Spatial distribution of global forest loss drivers at 0.25° resolution during 2001–2015. The maps display the area fraction for six distinct categories of forest cover loss: areas with zero or minor loss (i.e., regions without stand-replacing disturbance), alongside five specific forest loss drivers (commodity-driven deforestation, shifting agriculture, forestry, wildfire, and urbanization). To provide empirical context for the AGC dynamics observed in our study, the original high-resolution driver classification map from Curtis et al. [7] for the period 2001–2015 was spatially aggregated to our 0.25° grid. Grid-cell values ranging from 0.0 to 1.0 represent the areal proportion of forest loss attributed to each specific driver within the grid cell.

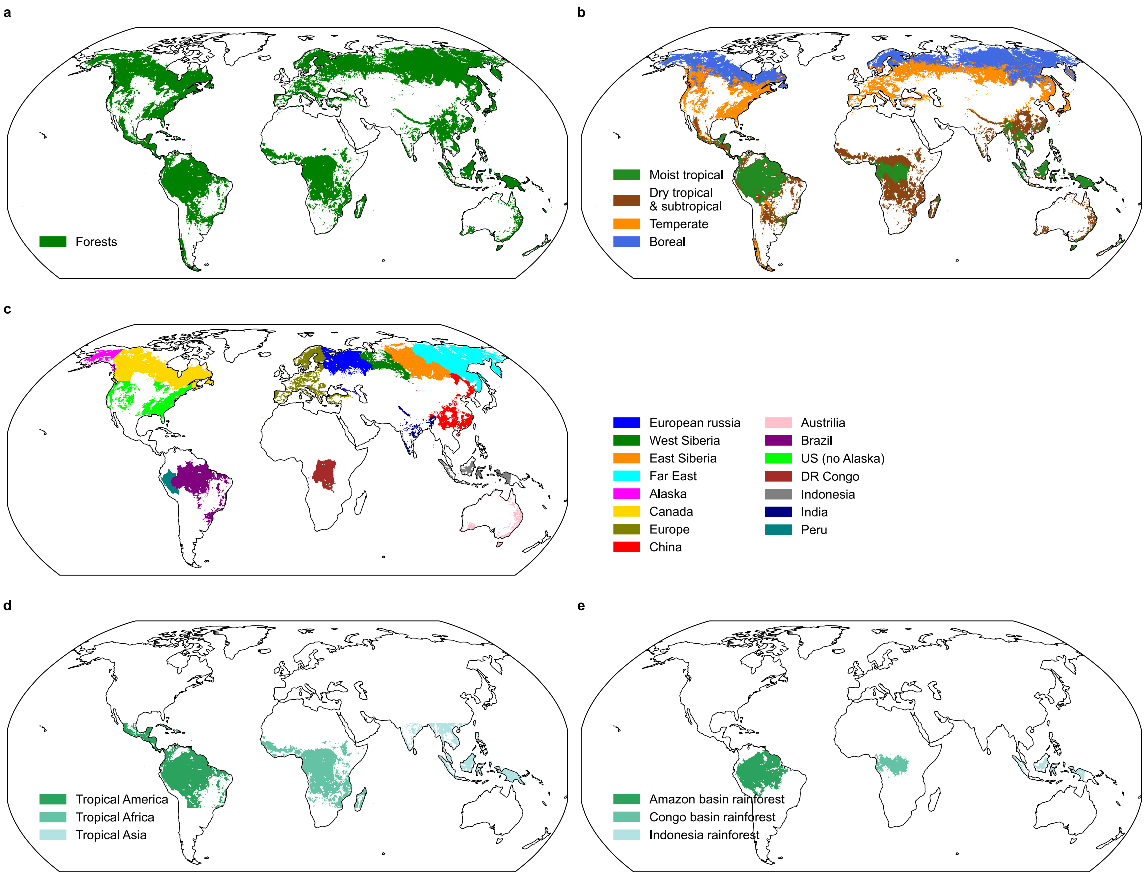

Supplementary Figure 16 Spatial masks used for analyzing global AGC distribution and dynamics. a, Global forest mask derived using the ESA CCI Land Cover dataset following the method from ref. [8]. This mask ensures consistent forest area representation over the analysis period. **b**, Forest biome classification based on ref. [4], delineating moist tropical, dry tropical & subtropical, temperate, and boreal biomes. **c**, Country-level masks for regions with the largest forest areas, as reported by the FAO, obtained from the Database of Global Administrative Areas (GADM). **d**, Pan-tropical region mask, spanning approximately 23.5°N to 23.5°S. **e**, Rainforest boundaries for the Amazon Basin, Congo Basin, and Indonesia, sourced from ArcGIS Online and GADM, extracted using the moist tropical forest mask. All masks were harmonized to a 0.25° grid to ensure spatial consistency in AGC analyses.

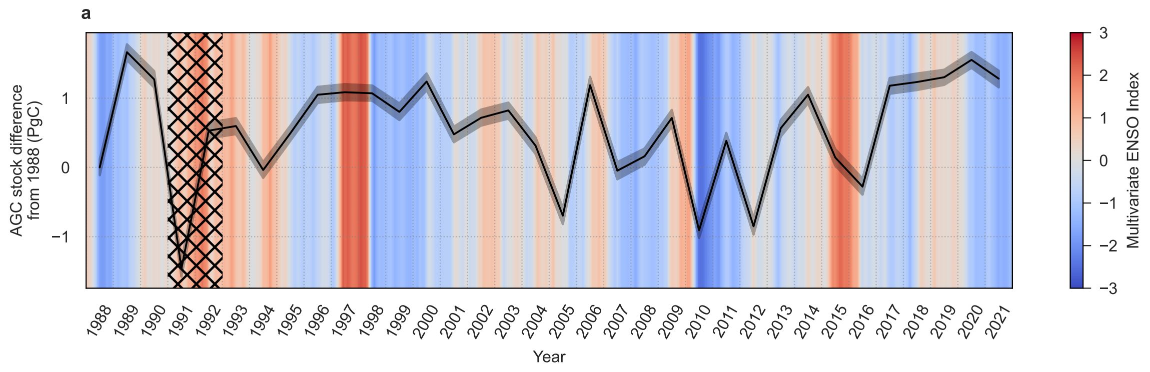

Supplementary Figure 17 Tropical AGC dynamics and ENSO variability from 1988 to 2021. Time series of AGC changes for all pan-tropical forests since 1988 in relation to ENSO phases. The shaded cross-hatched region is the period of volcanic eruption (1991-1992). Vertical bands show the intensity of La Niña (blue) and El Niño (red) events sourced from Multivariate ENSO Index Version 2.

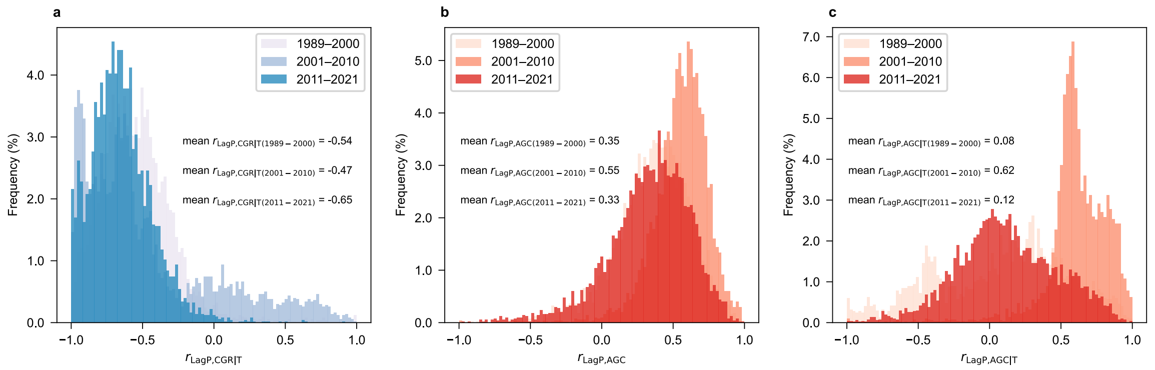

Supplementary Figure 18 Correlations between precipitation anomalies, AGC fluxes, and atmospheric CO_2 growth rate over three decades. a, Partial correlations between the interannual variability in six-month-lagged precipitation anomalies and atmospheric CO_2 growth rate, controlling for temperature ($r_{\mathrm{LagP,CGR|T}}$). **b**, Pearson correlations between the interannual variability in six-month-lagged precipitation anomalies and AGC fluxes ($r_{\mathrm{LagP,AGC}}$). **c**, Partial correlations between the interannual variability in six-month-lagged precipitation anomalies and AGC fluxes, controlling for temperature ($r_{\mathrm{LagP,AGC|T}}$). Distributions are estimated from 5000 bootstrap samples.

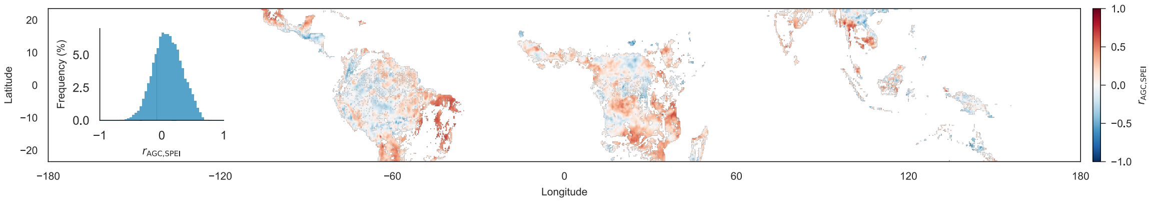

Supplementary Figure 19 Spatial correlation between AGC fluxes and SPEI at the grid-cell level. Pearson correlation coefficients between annual AGC density fluxes and concurrent SPEI03 values, computed at 0.25 ° resolution across the tropical domain from 1989 to 2021, excluding the years 1991–1993. A positive correlation indicates that SPEI and AGC fluxes are directionally aligned, i.e., wetter years are associated with AGC gain and drier years with AGC loss. Conversely, a negative correlation implies a mismatch in direction. Distribution of correlation coefficients shown in the map, indicating a weak overall correlation with a median Pearson's r of 0.09.

Supplementary Tables

Supplementary Table 1 Summary of predictor datasets used in AGC estimation.

Type	Predictors	Data source	Short description	Processing
Dynamic	CXKu-band vegetation optical depth (VOD)	VODCAv2[9]	A multi-sensor, multi-frequency microwave VOD (1987–2021) that captures upper canopy dynamics, vegetation water content, and biomass variations, offering improved temporal sampling and lower random errors compared to single-frequency VOD.	Annual mean and 95% quantile during growing seasons temporal resampling
	Normalized difference vegetation index (NDVI)	PKU GIMMS NDVI [10]	A global NDVI (1982–2022) derived from AVHRR and MODIS using machine learning and data consolidation with high-quality Landsat samples, correcting orbital drift and sensor degradation while improving accuracy over previous GIMMS NDVI.	Annual mean during growing seasons temporal resampling
	Leaf area index (LAI)	GIMMS LAI4g [11]	A global LAI (1982–2020) generated using a back-propagation neural network and high-quality Landsat LAI samples, effectively correcting orbital drift and sensor degradation while improving spatiotemporal consistency and accuracy over previous LAI products.	Annual mean during growing seasons temporal resampling; one year (2021) extending using ARIMA
	Plant functional types (PFTs) of trees	ESA CCI Land Cover [12]	A dataset providing annual fractional cover (1992–2021) for four tree plant functional types—broadleaved evergreen, broadleaved deciduous, needle-leaved evergreen, and needle-leaved deciduous—derived from ESA CCI Land Cover, improving representation of forest dynamics in Earth system models.	Aggregating to 0.25 ° using the ESA CCI user tool; extending the years 1988–1991 using ARIMA
	Forest cover fractions	ESA CCI Land Cover [12]	Annual forest cover fraction (1992–2021) derived from ESA CCI Land Cover by aggregating relevant forest-related classes, including broadleaved and needle-leaved forests (evergreen and deciduous), mixed leaf forests, mosaic tree and shrub cover (> 50%), and flooded forests.	Calculating land cover fraction and aggregating to 0.25 ° using the ESA CCI user tool; extending the years 1988–1991 using ARIMA
Static	L-band VOD	VODCAv2 [9]	A low-frequency microwave VOD (2010–2021) derived from SMOS and SMAP observations, offering deeper canopy penetration and stronger sensitivity to trunks and branches, with 10-day global coverage at 0.25 ° resolution.	Multi-year mean and 95th percentile during growing seasons across all years
	Digital elevation model DEM	ETOPO 2022 [13]	A high-resolution (15-arc-second) global DEM integrating multiple topographic and bathymetric datasets, providing seamless bare-earth elevation data for land, ocean, and ice surfaces with improved accuracy and global coverage.	Extracting land DEM and aggregating to 0.25 °
	Photosynthetically active radiation (PAR)	MODIS MCD18C2 [14]	A global MODIS-derived PAR product providing daily estimates at 0.05 ° resolution, derived using a look-up table approach that accounts for aerosols, clouds, and illumination conditions.	Multi-year mean and 0.25 ° aggregation
	Geographic coordinates	/	Cycle encoding of the geographic coordinates of the grid.	Sine and cosine transformations to longitude

Note: A total of 16 predictors are used, including CXKu-band VOD (annual growing season mean and Q95), NDVI (annual growing season mean), LAI (annual growing season mean), PFTs of trees (broadleaved deciduous, broadleaved evergreen, needle-leaved deciduous, needle-leaved evergreen), forest fractions, L-band VOD (growing season mean and Q95 across years), DEM, PAR, and geographic coordinates (latitude, sine and cosine of longitude).

Supplementary Table 2 Summary of reference datasets.

Reference	Spatiotemporal information	Short description
ESA CCI AGB [15]	Global; 100 m (native) – 50 km (aggregated); 2010, 2015–2021 (annual data)	ESA CCI AGB is a global biomass dataset to support carbon cycle and climate studies. It provides spatially explicit estimates of forest AGB (Mg ha^{-1}), enabling assessments of carbon stocks, fluxes, and forest disturbances. The dataset is derived using synthetic aperture radar (SAR) and LIDAR remote sensing, ensuring global consistency by leveraging methodologies from the ESA GlobBiomass project. The target accuracy is a relative error of less than 20% where AGB exceeds 50 Mg ha^{-1}.
Liu et al. AGB [1]	Global; 25 km; 1993–2012 (annual data)	Liu et al. estimated AGB using an empirical arctan-based VOD-AGB relationship applied to Ku&X-band VOD, which is sensitive to vegetation structure and biomass. To calibrate this relationship, they used the Saatchi et al. AGB map [16] for tropical regions as a reference, ensuring the conversion of VOD to above-ground biomass carbon was aligned with an established large-scale biomass dataset.
Avitabile et al. AGB [2]	Global; 1 km; ca. 2000	Avitabile et al. initially developed a pan-tropical AGB map by integrating two existing large-scale biomass maps [16, 17] with high-quality local observations and later expanded it into a global AGB map by incorporating boreal forest biomass data[18], providing improved spatial coverage and accuracy.
Fan et al. AGB [3]	Pantropical; 25 km; 2010–2017 (annual data)	Fan et al. estimated AGC using an empirical arctan-based VOD-AGB relationship applied to L-VOD from SMOS, leveraging its sensitivity to total vegetation water content and strong correlation with biomass, particularly in woody vegetation. To calibrate L-VOD and improve AGC retrievals, they incorporated multiple static benchmark AGC maps [2, 16, 17, 19], reducing dependence on a single biomass dataset and enhancing spatial consistency.
Xu et al. AGB [4]	Global; 10 km; 2000–2019 (annual data)	Xu et al. estimated total living AGB globally from 2000 to 2019 using a random forest model trained on GLAS lidar, airborne lidar, and ALOS-derived biomass data, incorporating bias correction to improve predictions. Since their dataset represents total living biomass, we applied grid-cell-wise root-to-shoot ratios [20] to decompose it into aboveground and belowground biomass components for consistency with other AGB datasets.
Boitard et al. AGB [5]	Global; 25 km; 2011–2023 (annual data)	Boitard et al. estimated global AGB from 2011 onward using L-band VOD from the SMOS mission as a proxy. They calibrated a global VOD-AGB relationship using logistic functions against the ESA CCI AGB and applied it to annual VOD data to derive a time series. Their dataset, which captures interannual biomass variability, shows overall consistency with ESA CCI AGB while generally presenting lower AGB estimates.
GEDI L4B AGB [6]	Global (51.6 ° N – 51.6 ° S; sparse); 1 km; ca.2020	GEDI L4B provides mean AGB density estimates from April 2019 to August 2021, derived from GEDI L4A footprint biomass observations using a statistical inference approach. As a spaceborne lidar mission aboard the International Space Station (ISS), GEDI samples Earth's 3D forest structure, offering the highest-resolution global biomass estimates among orbital lidar systems. The dataset includes uncertainty estimates accounting for sampling limitations and model-based inference errors.

Supplementary Table 3 Decadal AGC net changes and trends across biomes, countries/regions, and tropical regions.

Category	Region	1988 – 2000		2001 – 2010		2011 – 2021		1988 – 2021		
		Net change (TgC year^{-1})	Trend (TgC year^{-1})	Net change (TgC year^{-1})	Trend (TgC year^{-1})	Net change (TgC year^{-1})	Trend (TgC year^{-1})	Net change (TgC year^{-1})	Trend (TgC year^{-1})	Total net change (TgC)
**Global**		210.1 (194.2 – 226.0)	105.5 (78.7 – 131.9)	43.0 (22.1 – 63.7)	125.5 (94.8 – 159.8)	196.3 (176.8 – 215.4)	265.9 (243.5 – 286.8)	187.8 (181.9 – 193.5)	182.2 (178.0 – 186.3)	6196.2 (6001.1 – 6386.8)
**Biome**	**Moist tropical**	90.2 (79.0 – 102.2)	54.4 (42.9 – 66.8)	-190.6 (-206.2 – -174.2)	-122.0 (-142.6 – -100.8)	-4.7 (-18.1 – 9.3)	5.6 (-7.8 – 20.1)	-3.2 (-7.6 – 1.3)	-22.3 (-24.7 – -19.8)	-106.8 (-251.9 – 42.5)
	**Dry tropical & subtropical**	16.9 (9.7 – 24.2)	-17.8 (-28.7 – -8.6)	54.3 (45.0 – 64.4)	72.4 (60.6 – 87.9)	97.2 (88.5 – 106.0)	129.6 (119.8 – 141.4)	59.2 (56.6 – 61.8)	46.1 (43.8 – 48.5)	1955.2 (1868.5 – 2038.6)
	**Temperate**	92.6 (86.2 – 99.7)	80.5* (74.0 – 87.4)	93.1 (84.2 – 102.3)	73.6* (56.9 – 88.9)	106.6 (98.6 – 114.9)	65.0* (48.2 – 78.3)	94.0 (91.4 – 96.5)	93.0* (91.1 – 95.0)	3102.2 (3016.2 – 3184.6)
	**Boreal**	9.9 (5.5 – 14.3)	0.0 (-7.7 – 7.0)	85.8 (80.2 – 91.6)	75.3 (59.6 – 84.7)	-2.5 (-7.5 – 3.0)	-0.1 (-6.8 – 8.1)	38.0 (36.5 – 39.6)	60.6 (59.4 – 61.7)	1253.0 (1203.7 – 1305.5)
**Country/region**[1]	**Russia**	75.2 (70.5 – 79.9)	58.8 (49.9 – 66.6)	119.0 (112.4 – 125.5)	114.8 (105.9 – 121.6)	41.6 (35.3 – 47.7)	50.9 (41.1 – 59.6)	89.6 (88.0 – 91.5)	92.1 (90.6 – 93.6)	2957.9 (2904.1 – 3019.0)
	**European Russia**	29.1 (26.4 – 31.7)	25.5 (23.0 – 28.4)	51.8 (48.4 – 55.2)	24.7 (18.5 – 30.7)	14.6 (11.9 – 17.8)	15.3 (12.2 – 19.5)	25.6 (24.7 – 26.5)	25.7 (25.0 – 26.4)	844.8 (814.3 – 874.7)
	**West Siberia**	26.7 (24.5 – 29.0)	21.5 (18.4 – 24.8)	4.0 (1.0 – 7.1)	10.3 (7.3 – 14.7)	36.2 (33.5 – 38.8)	35.0 (31.1 – 38.0)	25.8 (25.0 – 26.5)	19.5 (18.9 – 20.1)	850.2 (824.2 – 876.1)
	**East Siberia**	9.7 (7.3 – 12.4)	4.4 (2.4 – 6.6)	19.0 (15.7 – 22.4)	15.7 (11.7 – 19.1)	13.2 (10.2 – 16.3)	32.6 (29.0 – 36.4)	18.1 (17.2 – 19.0)	18.9 (18.4 – 19.5)	596.3 (566.1 – 625.7)
	**Far East**	9.6 (7.1 – 11.9)	2.2 (-1.7 – 5.8)	44.3 (40.9 – 48.0)	47.8 (43.0 – 54.5)	-22.6 (-25.6 – -19.6)	-5.3 (-10.3 – 0.2)	20.2 (19.2 – 21.1)	25.2 (24.6 – 25.7)	666.3 (634.8 – 697.2)
	**Brazil**	11.6 (4.8 – 19.1)	-15.0 (-22.8 – -7.2)	-143.1 (-152.2 – -133.5)	-84.4 (-105.7 – -66.7)	-1.3 (-9.9 – 7.4)	45.1 (32.8 – 56.5)	-14.5 (-17.1 – -12.0)	-29.6 (-31.8 – -27.6)	-478.2 (-563.0 – -396.3)
	**Canada**	-20.0 (-23.7 – -16.1)	-15.4 (-20.6 – -9.6)	24.5 (18.9 – 29.7)	10.0 (4.8 – 15.2)	-6.0 (-10.7 – -1.0)	-37.4 (-55.4 – -23.2)	-9.6 (-11.0 – -8.0)	11.4 (9.9 – 13.0)	-316.1 (-361.5 – -263.5)
	**US (no Alaska)**	16.3 (12.7 – 19.7)	24.4* (21.2 – 27.8)	13.1 (8.5 – 17.8)	-10.2* (-14.6 – -6.0)	37.5 (33.4 – 41.8)	29.7* (25.6 – 33.2)	21.6 (20.3 – 22.8)	19.6* (18.8 – 20.4)	711.6 (670.4 – 751.3)
	**Alaska**	1.9 (1.0 – 2.8)	1.7 (0.9 – 2.4)	1.5 (0.3 – 2.6)	0.3 (-1.8 – 2.2)	27.0 (25.9 – 28.1)	20.2 (18.4 – 22.2)	7.4 (7.1 – 7.8)	5.8 (5.6 – 6.1)	245.3 (234.1 – 257.5)
	**China**	3.0 (0.2 – 5.7)	3.0 (0.1 – 5.9)	3.0 (-0.6 – 6.7)	20.8 (16.6 – 25.1)	30.4 (27.2 – 33.7)	32.2 (28.3 – 36.6)	18.1 (17.1 – 19.1)	19.2 (18.5 – 19.8)	597.9 (565.1 – 631.4)
	**Europe**	16.7 (14.4 – 18.9)	13.9* (10.5 – 16.6)	20.1 (17.4 – 22.9)	25.4* (22.1 – 28.6)	-10.5 (-13.4 – -7.9)	2.6* (-0.3 – 5.2)	14.1 (13.3 – 14.9)	15.8* (15.2 – 16.3)	465.4 (437.7 – 493.1)
	**Australia**	-2.8 (-4.4 – -1.1)	-2.8 (-3.9 – -1.6)	8.8 (6.7 – 11.0)	1.4 (-2.2 – 4.8)	-14.8 (-16.8 – -12.8)	-13.0 (-15.0 – -11.0)	-2.5 (-3.1 – -1.9)	-1.6 (-2.0 – -1.2)	-81.3 (-101.1 – -61.7)
	**DR Congo**	20.6 (14.7 – 26.0)	13.8 (8.4 – 19.7)	22.9 (14.6 – 30.9)	13.9 (6.3 – 20.8)	13.1 (5.8 – 20.4)	14.1 (6.9 – 21.2)	7.7 (5.5 – 9.6)	1.8 (0.8 – 2.9)	253.0 (183.1 – 318.1)
	**Indonesia**	-2.0 (-4.7 – 0.9)	-5.4 (-7.6 – -3.1)	-8.2 (-11.6 – -4.5)	-8.3 (-11.6 – -5.2)	4.7 (1.4 – 8.0)	0.1 (-3.0 – 3.3)	-3.0 (-4.1 – -2.0)	-3.5 (-4.0 – -3.0)	-99.7 (-135.0 – -65.3)
	**India**	1.1 (-0.2 – 2.4)	1.0 (-0.2 – 2.1)	1.4 (-0.3 – 3.3)	2.8 (1.2 – 4.4)	7.6 (5.9 – 9.4)	9.8 (8.2 – 11.4)	5.1 (4.5 – 5.5)	3.6 (3.3 – 3.9)	166.9 (150.0 – 183.0)
	**Peru**	1.6 (-1.8 – 4.6)	0.8 (-1.4 – 3.1)	-17.8 (-22.2 – -13.7)	-13.5 (-18.1 – -9.0)	1.4 (-2.5 – 5.1)	8.6 (4.9 – 12.3)	0.6 (-0.5 – 1.8)	-0.1 (-0.7 – 0.5)	19.4 (-17.5 – 58.8)
**Tropics**	**Pantropical**	103.0 (89.3 – 116.0)	49.0 (18.4 – 75.1)	-154.2 (-171.6 – -136.5)	-82.8 (-104.9 – -60.7)	89.6 (72.8 – 105.8)	129.3 (113.7 – 145.2)	38.6 (34.0 – 43.4)	2.7 (-0.6 – 6.1)	1275.2 (1121.7 – 1432.2)
	**Tropical America**	43.9 (34.7 – 53.1)	-1.9 (-15.0 – 11.0)	-206.1 (-218.5 – -192.3)	-148.7 (-181.7 – -113.2)	5.5 (-7.2 – 16.9)	48.8 (37.9 – 60.5)	-7.7 (-11.2 – -4.3)	-26.3 (-28.6 – -24.1)	-255.7 (-369.4 – -142.6)
	**Tropical Africa**	51.6 (43.7 – 59.7)	39.6 (25.3 – 52.7)	59.9 (47.9 – 71.5)	59.2 (45.7 – 71.9)	65.2 (54.7 – 74.7)	86.5 (73.6 – 99.3)	42.1 (39.1 – 45.1)	25.3 (23.5 – 27.2)	1388.4 (1291.7 – 1488.7)
	**Tropical Asia**	7.6 (3.1 – 12.3)	-1.3 (-5.2 – 2.8)	-7.9 (-13.6 – -1.9)	-2.6 (-8.4 – 3.1)	18.3 (12.6 – 23.9)	15.9 (11.1 – 20.1)	4.5 (2.8 – 6.1)	2.2 (1.3 – 3.1)	150.0 (91.7 – 200.2)
	**Amazon Basin Rainforest**	37.5 (29.1 – 46.2)	18.1 (9.8 – 25.8)	-180.3 (-191.2 – -168.8)	-115.8 (-132.2 – -100.7)	1.8 (-8.8 – 11.3)	31.2 (21.6 – 41.0)	-9.8 (-12.6 – -6.7)	-22.3 (-24.4 – -20.4)	-323.3 (-417.0 – -220.1)
	**Congo Basin Rainforest**	29.7 (23.6 – 36.2)	22.5 (16.9 – 28.5)	1.0 (-7.7 – 10.0)	3.3 (-4.4 – 10.7)	-16.7 (-24.9 – -8.6)	-10.4 (-21.1 – 1.0)	0.2 (-2.1 – 2.5)	-1.4 (-2.7 – 0.0)	7.2 (-67.9 – 82.8)
	**Indonesia Rainforest**	-1.8 (-4.3 – 0.7)	-4.8 (-6.9 – -2.7)	-8.6 (-12.1 – -5.1)	-8.4 (-11.5 – -5.5)	3.7 (0.5 – 6.9)	-0.2 (-3.1 – 2.3)	-3.1 (-4.1 – -2.2)	-3.6 (-4.1 – -3.2)	-103.9 (-136.6 – -73.8)

Note: The extent of these regions is shown in Fig. 16b, c, d, and e. red color represents a negative AGC net change, and the brown color denotes a negative AGC trend. The net change shown here represents the annual mean AGC difference over a specific period. For trend calculation, we consider only grid cells with valid data across all years, and AGC stock values for 1991 and 1992 are set to no data. All trends are calculated using the Theil-Sen slope, and * indicates a p-value < 0.05 using the modified Mann-Kendall test. Values in parentheses show 95% uncertainty interval (see Methods).

[1]The countries and regions listed are those with the largest forest areas, as identified based on data compiled by the Food and Agriculture Organization of the United Nations (FAO).

Supplementary Table 4 Signal-to-noise ratio (SNR) of decadal AGC net changes and trends across biomes, countries/regions, and tropical regions.

Category	Region	1988 – 2000		2001 – 2010		2011 – 2021		1988 – 2021	
		SNR of net change	SNR of trend	SNR of net change	SNR of trend	SNR of net change	SNR of trend	SNR of net change	SNR of trend
**Global**		24.5	6.8	4.1	8.3	18.5	24.5	63.6	83.6
**Biome**	**Moist tropical**	15.0	8.6	22.2	11.8	0.6	0.8	1.5	17.5
	**Dry tropical & subtropical**	4.4	3.3	11.2	10.1	22.3	25.6	43.3	40.6
	**Temperate**	26.9	22.2	19.3	9.5	25.4	8.4	72.5	94.5
	**Boreal**	4.8	0.0	30.3	11.2	0.8	0.0	47.0	102.0
**Country/region**[1]	**Russia**	30.6	14.1	36.5	28.5	13.9	10.4	96.0	129.1
	**European Russia**	21.3	19.3	29.8	7.5	9.1	9.2	49.2	69.6
	**West Siberia**	24.1	13.3	2.6	6.0	25.2	19.5	67.7	63.7
	**East Siberia**	7.4	3.8	11.4	8.2	8.4	18.4	39.7	59.5
	**Far East**	7.5	1.1	27.0	16.2	14.5	2.0	45.4	80.0
	**Brazil**	3.1	3.8	30.5	8.5	0.3	7.2	11.0	27.2
	**Canada**	10.4	5.7	9.4	3.7	2.3	4.5	12.6	14.6
	**US (no Alaska)**	10.1	14.4	5.8	4.4	17.3	14.5	35.0	50.6
	**Alaska**	4.2	4.5	2.4	0.3	48.5	21.9	43.1	49.6
	**China**	2.1	2.2	1.6	9.6	18.0	15.5	35.7	54.7
	**Europe**	14.4	9.1	12.8	16.0	7.5	2.0	34.2	66.5
	**Australia**	3.5	4.6	7.8	0.8	14.4	12.8	8.2	8.4
	**DR Congo**	7.3	4.5	5.7	3.9	3.8	4.1	7.5	3.7
	**Indonesia**	1.5	4.8	4.4	5.0	2.7	0.1	6.0	14.0
	**India**	1.6	1.6	1.6	3.1	8.8	11.6	20.6	22.5
	**Peru**	0.9	0.6	7.7	5.6	0.7	4.7	1.1	0.3
**Tropics**	**Pantropical**	15.1	3.4	16.6	7.1	10.7	15.8	15.0	1.7
	**Tropical America**	8.8	0.3	31.3	9.0	1.0	8.9	4.4	22.5
	**Tropical Africa**	11.9	5.4	10.0	9.7	12.0	13.1	26.9	27.6
	**Tropical Asia**	3.5	0.6	2.6	0.8	6.8	6.8	5.6	4.7
	**Amazon Basin Rainforest**	9.1	4.3	31.7	13.8	0.3	6.1	6.3	20.8
	**Congo Basin Rainforest**	8.6	7.7	0.3	0.9	4.0	1.9	0.1	2.0
	**Indonesia Rainforest**	1.4	4.3	4.6	5.3	2.4	0.1	6.3	14.9

Note: The extent of these regions is shown in Fig. 16b, c, d, and e. The SNR values are derived using a Monte Carlo ensemble approach ($N = 1000$) to rigorously propagate grid-cell-level predictive uncertainties to the aggregated regional scales, providing robust reliability support for the estimated AGC net changes and trends. For trend calculation, we consider only grid cells with valid data across all years, and AGC stock values for 1991 and 1992 are set to no data.

[1]The countries and regions listed are those with the largest forest areas, as identified based on data compiled by the Food and Agriculture Organization of the United Nations (FAO).

Supplementary Table 5 Correlation of AGB time series among observation products across tropical forest regions during overlapping years.

Comparison	Pantropical	Trop. Amer.	Amazon	Trop. Afr.	Congo	Trop. Asia	Indonesia
**Pearson's r for AGC stock time series**							
Ours vs Liu et al. [1] (1993–2012)	0.57**	0.71***	0.79***	0.40	-0.20	0.51*	0.87***
Ours vs Xu et al. [4] (2000–2019)	0.45*	0.68***	0.60**	0.44	-0.64**	-0.22	0.15
Ours vs Fan et al. [3] (2010–2017)	0.33	0.21	0.03	0.60	0.65	0.56	0.18
Ours vs Boitard et al. [5] (2011–2021)	-0.22	-0.59	-0.63*	-0.16	0.25	0.65*	0.49
Liu et al. vs Xu et al. (2000–2012)	0.18	0.52	0.74**	0.46	0.62*	0.40	0.63*
Xu et al. vs Fan et al. (2010–2017)	0.20	0.51	0.64	-0.45	-0.85**	0.16	-0.53
Xu et al. vs Boitard et al. (2011–2019)	-0.28	-0.88**	-0.95***	-0.22	-0.26	0.17	-0.03
Fan et al. vs Boitard et al. (2011–2017)	-0.81*	-0.61	-0.75	0.28	-0.47	-0.09	-0.32
**Pearson's r for AGC flux time series**							
Ours vs Liu et al. (1994–2012)	0.56*	0.36	0.24	0.42	0.12	0.61**	0.32
Ours vs Xu et al. (2001–2019)	0.71***	0.62**	0.48*	0.05	-0.25	0.05	-0.24
Ours vs Fan et al. (2011–2017)	0.66	0.66	0.51	0.74	0.49	0.39	-0.43
Ours vs Boitard et al. (2012–2021)	-0.23	-0.22	-0.49	0.18	0.05	-0.30	0.00
Liu et al. vs Xu et al. (2001–2012)	0.36	0.36	0.17	0.12	0.42	0.27	-0.29
Xu et al. vs Fan et al. (2011–2017)	0.65	0.66	0.44	-0.31	-0.60	0.46	-0.19
Xu et al. vs Boitard et al. (2012–2019)	-0.38	-0.69	-0.92**	-0.09	-0.12	0.07	0.19
Fan et al. vs Boitard et al. (2012–2017)	-0.14	0.20	-0.28	0.54	0.15	-0.41	0.57

Note: Asterisks denote significance levels: * $p < 0.05$, ** $p < 0.01$, *** $p < 0.001$, based on two-tailed t-test. Abbreviations: Trop. Amer., Tropical America; Trop. Afr., Tropical Africa; Trop. Asia, Tropical Asia. Amazon, Congo, and Indonesia refer to their respective basin rainforests.

Supplementary Table 6 Hyperparameter settings for our deep learning model

Item	Hyperparameter
Window slicing[1]	Training: window size = 15, sliding step = 9
	Validation and test: window size = 15, sliding step = 15
Batch size[2]	256
Noise rate[3]	0.25
Learning rate[4]	0.01 (pretraining), 0.0001 (fine-tuning)
Loss function[5]	MSE (pretraining), Gaussian NLL (fine-tuning)
Optimizer[6]	AdamW
Weight decay[7]	0.001
Scheduler type[8]	Step decay
Scheduler step size[9]	24
Scheduler gamma[10]	0.25
Maximum training epochs[11]	256
Early stopping patience[12]	25

[1]Window slicing is a data preparation hyperparameter used to extract patches from predictors and ESA CCI AGB via a sliding window. For training data, we set the sliding step (9 grid cells) smaller than the window size (15 grid cells), which expands the dataset while preserving spatial continuity and avoiding the loss of edge grid cells.

[2]Batch size determines the number of samples processed in each training iteration, balancing memory efficiency and model convergence stability.

[3]Noise rate refers to the fraction of grid cells that are randomly masked, acting as a regularization method to prevent model overfitting.

[4]Learning rate controls the step size of model weight updates during training, influencing convergence speed and stability. A higher learning rate (0.01) is used during pretraining to enable faster exploration of the loss landscape, while a lower learning rate (0.0001) in fine-tuning ensures more precise adjustments and prevents overshooting, refining the model on the target task.

[5]Loss function determines how the model optimizes its predictions. During pretraining, Mean Squared Error (MSE) is used to first learn the expected AGB values, ensuring stable convergence. In fine-tuning, Gaussian Negative Log-Likelihood (NLL) is applied to model both the mean and variance, enabling the network to capture prediction uncertainty. We found that directly training with Gaussian NLL led to poor convergence, so pretraining with MSE helps establish a strong initial representation before refining the uncertainty estimation.

[6]The optimizer updates model weights during training to minimize the loss function, ensuring efficient convergence. AdamW is used for optimization, which combines the benefits of Adam (adaptive learning rates) with decoupled weight decay to improve generalization.

[7]In AdamW, weight decay is decoupled from gradient updates, ensuring more effective regularization without interfering with adaptive learning rates.

[8]A learning rate scheduling strategy that reduces the learning rate by a fixed factor at predefined intervals (steps). This helps the model converge efficiently by making large updates initially and refining weights with smaller updates in later stages, improving stability and performance.

[9]The interval (number of epochs) after which the learning rate is reduced in step decay scheduling.

[10]The multiplicative factor by which the learning rate is reduced at each step, controlling the rate of decay.

[11]The upper limit on the number of training iterations, ensuring the model has sufficient opportunities to learn while preventing excessive computation.

[12]The number of consecutive epochs without improvement before training stops early, preventing overfitting and saving computational resources.

Supplementary Notes

Supplementary Note 1: Quality assessment of AGC reconstruction

To assess spatial consistency, we evaluate the spatial correlations between our modeled AGC estimates and six independent reference products (Supplementary Figs. 4 and 5). To ensure a fair comparison, we first calculate the temporal mean of both our dataset and each respective reference product over their specific overlapping time period (e.g., 1993–2012 for the Liu et al. dataset) to generate static AGC maps. We then compute the spatial Pearson correlation across all valid grid cells. At the global scale, these spatial correlations range from 0.70 to 0.87 depending on the reference product being compared, reaching a maximum of 0.92 in pan-tropical forests. Furthermore, we evaluate these spatial correlations separately within four distinct biomes. Although correlation coefficients decrease, the spatial consistency remains robust. The maximum spatial correlations achieved across the reference products are $r = 0.78$ in moist tropical forests, $r = 0.67$ in dry tropical and subtropical forests, $r = 0.77$ in temperate forests, and $r = 0.78$ in boreal forests. All reported correlations are statistically significant ($p < 0.001$). Lower correlations are observed with the Liu et al. [1] AGC and GEDI L4B AGC [6] datasets. This likely reflects biases introduced by the high-frequency Ku- and X-band VOD alongside empirical methods used in the Liu et al. product, as well as the sparse distribution of GEDI sampling, which can introduce systematic errors during aggregation to the 0.25° grid. Furthermore, cross-comparing the reference datasets (Supplementary Fig. 6) reveals that correlations generally decline from the global scale to individual biomes, underscoring the inherent inconsistencies in data acquisition and processing methods across products.

We then examine temporal agreement by comparing our reconstructed AGC stock and flux time series to the reference products (Table 2 and Supplementary Fig. 7). Globally, our AGC stock trends closely match Xu et al. (2000–2019; $r = 0.70, p < 0.001$), yet diverge from Boitard et al. (2011–2021; $r = -0.01, p > 0.05$) and Liu et al. (1993–2012; $r = -0.36, p > 0.05$), which shows a biologically implausible decline of global forest AGC [21, 22]. Biome-specific comparisons also exhibit good alignment with reference datasets, with statistically significant high correlation coefficients. For instance, we observe $r = 0.64, p < 0.01$ in moist tropical forests (1993–2012), $r = 0.85, p < 0.001$ and $r = 0.84, p < 0.001$ in dry tropical & subtropical forests (2000–2019 and 2011–2021), $r = 0.70, p < 0.001$ and $r = 0.92, p < 0.001$ in temperate forests (1993–2012 and 2000–2019), and $r = 0.61, p < 0.01$ and $r = 0.59, p < 0.01$ in boreal forests (1993–2012 and 2000–2019). The temporal agreement for annual AGC fluxes is generally lower than that for total AGC stocks. Nonetheless, our AGC flux estimates achieve maximum correlations exceeding $r = 0.5\,(p < 0.05)$ across all biomes, with the exception of boreal forests.

Notably, cross-comparisons among the various reference time series themselves reveal massive discrepancies in both stock trajectories and annual AGC fluxes. Compared with these references, our AGC reconstruction shows a more consistent agreement with individual reference datasets than they do with each other across long-term trajectories and interannual variability. This result does not only show in regionally aggregated AGC changes, but also in time series at the grid-cell level (Supplementary Fig. 8). While pixel-level temporal correlations are inherently lower across all comparisons due to high-frequency noise and local-scale discrepancies, our AGC records consistently yield higher median temporal correlations with individual references (median r ranging from 0.14 to 0.19) than those references achieve when cross-compared against one another (median r ranging from 0.04 to 0.06).

Overall, this spatiotemporal validation indicates a relatively high reliability of our AGC reconstruction. Our comparative analysis reveals that existing short-term reference datasets frequently exhibit systematic baseline offsets and even contradictory temporal trends. Consequently, simply concatenating these disjointed products across time to construct a long-term AGC record would introduce severe artificial shifts and uncertainty. This highlights the critical need for more comprehensive cross-comparison and methodological harmonization in global and biome-level biomass assessments.

Supplementary Discussion

Our spatially explicit AGC dataset complements the recent work by ref. [22], which assessed decadal carbon sinks in global natural forests over the past three decades based on compiled forest inventory data. In particular, our AGC estimates enable the identification of critical regions undergoing sink-to-source transitions. Furthermore, the detailed interannual variability in AGC fluxes facilitates a deeper exploration of forest carbon dynamics driven by climatic and anthropogenic disturbances. Conversely, inventory-based carbon estimates in ref. [22], despite their limitations in accuracy for tropical regions due to sparse inventory coverage, provide a comparative benchmark for assessing overall changes in the natural forest carbon sink. Integrating both results can enhance our understanding of global forest carbon dynamics and their underlying drivers.

Another recent study reported that the majority of terrestrial carbon accumulation between 1992 and 2019 occurred in nonliving organic matter pools, with gains in living biomass accounting for less than 5% of the total, corresponding to approximately 1 PgC [23]. In contrast, we estimate that forest living biomass increased by approximately 9.5 PgC over the same period. Assuming an AGB-to-belowground biomass ratio from ref. [20], this would account for roughly 25–30% of the total terrestrial carbon gain. Consequently, while our study focuses specifically on forested areas rather than entire terrestrial ecosystems, our findings suggest that although some imbalance likely exists, the contribution of living biomass to the overall carbon sink may be much larger than previously reported. One potential source of discrepancy may stem from the data harmonization approach. In particular, the AGC products in ref.[23] were, following typical procedures, harmonized through linear interpolation and averaging of existing datasets, which may introduce substantial uncertainty. Our analysis highlights that existing AGC reference datasets can differ considerably. Even products based on similar input data and methodologies may exhibit divergent changes or trends; for example, the datasets from ref.[3] and ref.[5], both based on L-VOD and empirical approaches, show substantial inconsistencies in AGC dynamics and interannual variability (Supplementary Fig. 14 and Table 5). While established AGC datasets provide valuable insights, the observed inconsistencies suggest that harmonizing them for long-term estimates can be highly complex. Our approach, grounded in independent, multi-source, state-of-the-art predictors, as well as a probabilistic deep learning approach, offers a promising way to minimize these compounding uncertainties.

Interestingly, our analysis reveals an intensifying negative correlation between annual tropical AGC fluxes and atmospheric CO_2 growth rate (Supplementary Fig. 13). This highlights the growing importance of tropical AGC dynamics in shaping the terrestrial carbon cycle variability. Our results are consistent with an earlier finding showing a strong association between tropical AGC fluxes and CO_2 growth rates during 2011–2017 [3]. Moreover, our results also provide independent support for previous exploratory evidence of a strengthening negative AGC–CO_2 correlation in tropics from 1989–2003 to 2002–2016 [24]. While the authors acknowledged uncertainties in their VOD-derived AGC estimates, particularly the sensitivity of VOD signals to soil moisture [25], our multivariable reconstruction approach helps mitigate these confounding effects. Moreover, our newly developed AGC estimates offer continuous decadal-scale evidence from 1989 to 2021, extending the previously observed relationship. This earlier study [24] further interpreted the intensifying negative AGC-CO_2 correlation as a reflection of strengthened water–carbon coupling in the tropics, likely associated with ENSO teleconnections, based primarily on a comparison between 1960–1989 and 1989–2018. However, our decadal-scale analysis from 1988–2021 does not reveal a consistent relationship between ENSO phases and AGC dynamics (Supplementary Fig.17), nor a clear pattern of intensifying negative water-carbon coupling (Supplementary Fig. 18a). Both the direct correlation between water availability and AGC flux, and the partial correlation after accounting for temperature effects, also show no clear strengthening trend over the 1989–2021 period (Supplementary Fig.18b,c). Notably, the decade 2001–2010 exhibits a marked shift in the coupling structures between water availability and both atmospheric CO_2 growth rate and AGC flux. This anomaly in 2001–2010 may be related to more moderate but frequent climatic fluctuations, as well as accelerating deforestation [26]. Our findings suggest that attributing the intensifying negative AGC-CO_2 correlation solely to ENSO-induced water stress is likely insufficient and oversimplified. In addition, improvements in observational data quality over time, such as changes in satellite retrieval algorithms or sensor characteristics, may have introduced additional sources of uncertainty into the observed AGC-CO_2 coupling. These considerations underscore the need for further investigation to disentangle the relative contributions of climatic and anthropogenic drivers.

Moreover, our results do not show a clear impact of environmental conditions, such as well-documented droughts, on the interannual tropical AGC dynamics, except to some extent in the Amazon. At the grid-cell level, the relationship between AGC flux and the SPEI is generally weak, with a median correlation coefficient of 0.09 across the pan-tropical forests (Supplementary Fig. 19). However, stronger positive correlations are observed in dry tropical and subtropical forests, likely due to their higher sensitivity to water availability [27, 28]. The overall weak drought fingerprint observed in AGC dynamics may result from several factors: First, interannual variability and long-term stock trajectories in tropical AGC differ across datasets (Supplementary Fig. 14 and Table. 5). Hence, the precision of AGC estimates may still not be high enough to observe drought responses sufficiently well, owing to ongoing uncertainties in existing datasets. In particular, recent studies have shown that purely VOD-derived AGC estimates may be influenced by changes in water stress, which could overstate biomass fluctuations during water scarcity periods [24, 25]. Second, AGC changes are influenced by environmental conditions in complex ways, and affected by multiple local disturbance factors, and by tree regrowth and reforestation. For example, drought is associated with lower cloud coverage and hence higher downwelling short-wave radiation, which may in some cases even increase gross primary productivity and hence AGC [29]. Consequently, further research is needed to clarify the extent to which drought events influence AGC storage.

References

[1] Liu, Y. Y. *et al.* Recent reversal in loss of global terrestrial biomass. *Nature Climate Change* **5**, 470–474 (2015).

[2] Avitabile, V. *et al.* An integrated pan-tropical biomass map using multiple reference datasets. *Global Change Biology* **22**, 1406–1420 (2016).

[3] Fan, L. *et al.* Satellite-observed pantropical carbon dynamics. *Nature Plants* **5**, 944–951 (2019).

[4] Xu, L. *et al.* Changes in global terrestrial live biomass over the 21st century. *Science Advances* **7**, eabe9829 (2021).

[5] Boitard, S. *et al.* Aboveground biomass dataset from SMOS L-band vegetation optical depth and reference maps. *Earth System Science Data* **17**, 1101–1119 (2025).

[6] Dubayah, R. *et al.* GEDI L4B gridded aboveground biomass density, version 2.1 (2023). URL https://daac.ornl.gov/cgi-bin/dsviewer.pl?ds_id=2299.

[7] Curtis, P. G., Slay, C. M., Harris, N. L., Tyukavina, A. & Hansen, M. C. Classifying drivers of global forest loss. *Science* **361**, 1108–1111 (2018).

[8] Besnard, S. *et al.* Global sensitivities of forest carbon changes to environmental conditions. *Global Change Biology* **27**, 6467–6483 (2021).

[9] Zotta, R.-M. *et al.* VODCA v2: Multi-sensor, multi-frequency vegetation optical depth data for long-term canopy dynamics and biomass monitoring. *Earth System Science Data* **16**, 4573–4617 (2024).

[10] Li, M. *et al.* Spatiotemporally consistent global dataset of the GIMMS Normalized Difference Vegetation Index (PKU GIMMS NDVI) from 1982 to 2022. *Earth System Science Data* **15**, 4181–4203 (2023).

[11] Cao, S. *et al.* Spatiotemporally consistent global dataset of the GIMMS leaf area index (GIMMS LAI4g) from 1982 to 2020. *Earth System Science Data* **15**, 4877–4899 (2023).

[12] CCI, E. L. C. Land Cover CCI Product user guide version 2.0. *UCL-Geomatics: London, UK* (2017).

[13] MacFerrin, M., Amante, C., Carignan, K., Love, M. & Lim, E. The earth topography 2022 (ETOPO 2022) global DEM dataset. *Earth System Science Data Discussions* **2024**, 1–24 (2024).

[14] Wang, D. *et al.* A New Set of MODIS Land Products (MCD18): Downward Shortwave Radiation and Photosynthetically Active Radiation. *Remote Sensing* **12**, 168 (2020).

[15] Santoro, M. *et al.* Design and performance of the Climate Change Initiative Biomass global retrieval algorithm. *Science of Remote Sensing* **10**, 100169 (2024).

[16] Saatchi, S. S. *et al.* Benchmark map of forest carbon stocks in tropical regions across three continents. *Proceedings of the National Academy of Sciences* **108**, 9899–9904 (2011).

[17] Baccini, A. *et al.* Estimated carbon dioxide emissions from tropical deforestation improved by carbon-density maps. *Nature Climate Change* **2**, 182–185 (2012).

[18] Santoro, M. *et al.* Forest growing stock volume of the northern hemisphere: Spatially explicit estimates for 2010 derived from Envisat ASAR. *Remote Sensing of Environment* **168**, 316–334 (2015).

[19] Rodríguez-Fernández, N. J. *et al.* An evaluation of SMOS L-band vegetation optical depth (L-VOD) data sets: High sensitivity of L-VOD to above-ground biomass in Africa. *Biogeosciences* **15**, 4627–4645 (2018).

[20] Spawn, S. A., Sullivan, C. C., Lark, T. J. & Gibbs, H. K. Harmonized global maps of above and belowground biomass carbon density in the year 2010. *Scientific Data* **7**, 112 (2020).

[21] Yang, H. *et al.* Global increase in biomass carbon stock dominated by growth of northern young forests over past decade. *Nature Geoscience* **16**, 886–892 (2023).

[22] Pan, Y. *et al.* The enduring world forest carbon sink. *Nature* **631**, 563–569 (2024).

[23] Bar-On, Y. M. *et al.* Recent gains in global terrestrial carbon stocks are mostly stored in nonliving pools. *Science* **387**, 1291–1295 (2025).

[24] Liu, L. *et al.* Increasingly negative tropical water–interannual CO2 growth rate coupling. *Nature* **618**, 755–760 (2023).

[25] Konings, A. G., Holtzman, N. M., Rao, K., Xu, L. & Saatchi, S. S. Interannual Variations of Vegetation Optical Depth are Due to Both Water Stress and Biomass Changes. *Geophysical Research Letters* **48**, e2021GL095267 (2021).

[26] Malhi, Y. *et al.* Climate Change, Deforestation, and the Fate of the Amazon. *Science* **319**, 169–172 (2008).

[27] Ahlström, A. *et al.* The dominant role of semi-arid ecosystems in the trend and variability of the land CO_2 sink. *Science* **348**, 895–899 (2015).

[28] Poulter, B. *et al.* Contribution of semi-arid ecosystems to interannual variability of the global carbon cycle. *Nature* **509**, 600–603 (2014).

[29] Jones, M. O., Kimball, J. S. & Nemani, R. R. Asynchronous Amazon forest canopy phenology indicates adaptation to both water and light availability. *Environmental Research Letters* **9**, 124021 (2014).
