# Supplementary material for: Decadal sink-source shifts of forest aboveground carbon since 1988
Source: Nat Commun. 2026 Jul 30;17:7600. doi: 10.1038/s41467-026-76093-3 (PMC13424356; doi:10.1038/s41467-026-76093-3)
Supplement: Supplementary file 2 — Reporting Summary [file 41467_2026_76093_MOESM2_ESM.pdf]

nature portfolio

Corresponding author(s): Zhen Qian, Niklas Boers

Last updated by author(s): May 24, 2026

# Reporting Summary

Nature Portfolio wishes to improve the reproducibility of the work that we publish. This form provides structure for consistency and transparency in reporting. For further information on Nature Portfolio policies, see our Editorial Policies and the Editorial Policy Checklist.

## Statistics

For all statistical analyses, confirm that the following items are present in the figure legend, table legend, main text, or Methods section.

| n/a | Confirmed | |
|---|---|---|
| ☐ | ☒ | The exact sample size (*n*) for each experimental group/condition, given as a discrete number and unit of measurement |
| ☐ | ☒ | A statement on whether measurements were taken from distinct samples or whether the same sample was measured repeatedly |
| ☐ | ☒ | The statistical test(s) used AND whether they are one- or two-sided<br>*Only common tests should be described solely by name; describe more complex techniques in the Methods section.* |
| ☐ | ☒ | A description of all covariates tested |
| ☐ | ☒ | A description of any assumptions or corrections, such as tests of normality and adjustment for multiple comparisons |
| ☐ | ☒ | A full description of the statistical parameters including central tendency (e.g. means) or other basic estimates (e.g. regression coefficient) AND variation (e.g. standard deviation) or associated estimates of uncertainty (e.g. confidence intervals) |
| ☒ | ☐ | For null hypothesis testing, the test statistic (e.g. *F*, *t*, *r*) with confidence intervals, effect sizes, degrees of freedom and *P* value noted<br>*Give P values as exact values whenever suitable.* |
| ☒ | ☐ | For Bayesian analysis, information on the choice of priors and Markov chain Monte Carlo settings |
| ☒ | ☐ | For hierarchical and complex designs, identification of the appropriate level for tests and full reporting of outcomes |
| ☐ | ☒ | Estimates of effect sizes (e.g. Cohen's *d*, Pearson's *r*), indicating how they were calculated |

*Our web collection on statistics for biologists contains articles on many of the points above.*

## Software and code

Policy information about availability of computer code

| | |
|---|---|
| Data collection | No custom software was used for primary data collection. Publicly available datasets (e.g., VODCA v2, PKU GIMMS LAI4g/NDVI) were downloaded from their respective official repositories. |
| Data analysis | All data preprocessing, statistical analyses, and machine learning modeling were conducted using Python (version 3.11). The Convolutional Neural Network (CNN) and probabilistic framework were implemented using PyTorch (version 2.2.2). Spatial data processing and the handling of geospatial raster/vector files were performed using xarray (v2026.1.0), geopandas (v1.1.2), and rasterio (v1.4.4). Data structuring and array operations were managed with pandas (v3.0.0) and numpy (v2.4.2). Statistical computations and evaluation metrics were conducted using scipy (v1.17.0), scikit-learn (v1.8.0), and statsmodels (v0.14.6). Data visualization and mapping were carried out using matplotlib (v3.10.8), cartopy (v0.25.0), and seaborn (v0.13.2). |

For manuscripts utilizing custom algorithms or software that are central to the research but not yet described in published literature, software must be made available to editors and reviewers. We strongly encourage code deposition in a community repository (e.g. GitHub). See the Nature Portfolio guidelines for submitting code & software for further information.

# Data

Policy information about availability of data

All manuscripts must include a data availability statement. This statement should provide the following information, where applicable:

- Accession codes, unique identifiers, or web links for publicly available datasets
- A description of any restrictions on data availability
- For clinical datasets or third party data, please ensure that the statement adheres to our policy

All predictors and reference data used in this study are derived from publicly available sources:
Climate and Environmental Data: The Standardized Precipitation Evapotranspiration Index (SPEI) data are available at https://spei.csic.es/. Global CO2 growth rates are available at https://gml.noaa.gov/ccgg/trends/gl_gr.html. CRU temperature and precipitation data are available at https://www.uea.ac.uk/groups-and-centres/climatic-research-unit.
Regional Data: Brazilian Amazon deforestation data (PRODES) are available at http://www.obt.inpe.br/OBT/assuntos/programas/amazonia/prodes.
Predictor Datasets: Dynamic and static predictors including VODCA v2, PKU GIMMS NDVI, GIMMS LAI4g, ESA CCI Land Cover (PFTs and Forest cover fractions), ETOPO 2022 DEM, and MODIS MCD18C2 PAR were obtained from their respective official repositories as detailed in the Methods and Supplementary Information.
Reference AGB/AGC Datasets: The reference aboveground biomass/carbon datasets used for model training and comparison, including ESA CCI AGB, GEDI L4B AGB, and products from Liu et al. (2015), Avitabile et al. (2016), Fan et al. (2019), Xu et al. (2021), and Boitard et al. (2025), are publicly accessible via the repositories associated with their respective publications.
The produced AGB datasets are available at https://doi.org/10.5281/zenodo.20365283.

# Research involving human participants, their data, or biological material

Policy information about studies with human participants or human data. See also policy information about sex, gender (identity/presentation), and sexual orientation and race, ethnicity and racism.

| | |
|---|---|
| Reporting on sex and gender | *Use the terms sex (biological attribute) and gender (shaped by social and cultural circumstances) carefully in order to avoid confusing both terms. Indicate if findings apply to only one sex or gender; describe whether sex and gender were considered in study design; whether sex and/or gender was determined based on self-reporting or assigned and methods used. Provide in the source data disaggregated sex and gender data, where this information has been collected, and if consent has been obtained for sharing of individual-level data; provide overall numbers in this Reporting Summary. Please state if this information has not been collected. Report sex- and gender-based analyses where performed, justify reasons for lack of sex- and gender-based analysis.* |
| Reporting on race, ethnicity, or other socially relevant groupings | *Please specify the socially constructed or socially relevant categorization variable(s) used in your manuscript and explain why they were used. Please note that such variables should not be used as proxies for other socially constructed/relevant variables (for example, race or ethnicity should not be used as a proxy for socioeconomic status). Provide clear definitions of the relevant terms used, how they were provided (by the participants/respondents, the researchers, or third parties), and the method(s) used to classify people into the different categories (e.g. self-report, census or administrative data, social media data, etc.) Please provide details about how you controlled for confounding variables in your analyses.* |
| Population characteristics | *Describe the covariate-relevant population characteristics of the human research participants (e.g. age, genotypic information, past and current diagnosis and treatment categories). If you filled out the behavioural & social sciences study design questions and have nothing to add here, write "See above."* |
| Recruitment | *Describe how participants were recruited. Outline any potential self-selection bias or other biases that may be present and how these are likely to impact results.* |
| Ethics oversight | *Identify the organization(s) that approved the study protocol.* |

Note that full information on the approval of the study protocol must also be provided in the manuscript.

# Field-specific reporting

Please select the one below that is the best fit for your research. If you are not sure, read the appropriate sections before making your selection.

☐ Life sciences ☐ Behavioural & social sciences ☒ Ecological, evolutionary & environmental sciences

For a reference copy of the document with all sections, see nature.com/documents/nr-reporting-summary-flat.pdf

# Ecological, evolutionary & environmental sciences study design

All studies must disclose on these points even when the disclosure is negative.

| | |
|---|---|
| Study description | This study reconstructs a harmonized, uncertainty-aware global forest aboveground carbon (AGC) record from 1988 to 2021 at a 0.25-degree resolution. It integrates multi-source satellite observations (including vegetation optical depth, NDVI, and LAI) with probabilistic deep learning models to assess decadal sink-source shifts and interannual variability in global forest ecosystems. |

| | |
|---|---|
| Research sample | The research sample comprises wall-to-wall, spatially explicit global forest grid cells at a 0.25-degree spatial resolution. The primary datasets include historical satellite observations (VODCAv2 CXKu-band and L-band VOD, PKU GIMMS NDVI, GIMMS LAI4g), ESA CCI Land Cover, MODIS PAR, ETOPO 2022 DEM, and ESA CCI AGC reference maps. |
| Sampling strategy | No statistical sampling methods were used to predetermine sample size. |
| Data collection | No new field data were collected. All data are derived from publicly available, long-term Earth observation satellite products, environmental data, and open-source administrative boundaries. |
| Timing and spatial scale | The study encompasses global forest ecosystems at a spatial resolution of 0.25-degree (approximately 25 km at the equator). The temporal scale is annual, spanning continuously over 34 years from 1988 to 2021. |
| Data exclusions | Grid cells with missing values across time were excluded from temporal analyses to ensure consistent spatial coverage and prevent data gap artifacts. To mitigate measurement uncertainties from stratospheric aerosols caused by the Mt. Pinatubo eruption, data from 1991–1992 were excluded from AGC stock trend analyses, and data from 1991–1993 were excluded from interannual flux variability analyses. Non-forest areas were excluded prior to analysis. |
| Reproducibility | To ensure the robustness of the deep learning models and avoid overfitting to specific years, an ensemble approach was used. Five independent probabilistic CNNs, each initialized randomly, were trained for each year between 2015 and 2020, yielding 30 model realizations at inference time. Predictive uncertainties were rigorously quantified using a Monte Carlo approach (drawing 1,000 samples per pixel per year). All custom code and datasets are available at https://doi.org/10.5281/zenodo.20365283. |
| Randomization | Not applicable. This is an observation-based Earth system science study relying on historical satellite remote sensing records. No experimental treatments were applied, making the random allocation of samples or experimental groups irrelevant to this research design. |
| Blinding | Not applicable. This study analyzes historical Earth observation data using automated deep learning algorithms. Blinding during data collection and analysis is not feasible or relevant to this purely observational and computationally driven study design. |

Did the study involve field work? ☐ Yes ☒ No

# Reporting for specific materials, systems and methods

We require information from authors about some types of materials, experimental systems and methods used in many studies. Here, indicate whether each material, system or method listed is relevant to your study. If you are not sure if a list item applies to your research, read the appropriate section before selecting a response.

## Materials & experimental systems

| n/a | Involved in the study | |
|---|---|---|
| ☒ | ☐ | Antibodies |
| ☒ | ☐ | Eukaryotic cell lines |
| ☒ | ☐ | Palaeontology and archaeology |
| ☒ | ☐ | Animals and other organisms |
| ☒ | ☐ | Clinical data |
| ☒ | ☐ | Dual use research of concern |
| ☒ | ☐ | Plants |

## Methods

| n/a | Involved in the study | |
|---|---|---|
| ☒ | ☐ | ChIP-seq |
| ☒ | ☐ | Flow cytometry |
| ☒ | ☐ | MRI-based neuroimaging |

## Plants

| | |
|---|---|
| Seed stocks | *Report on the source of all seed stocks or other plant material used. If applicable, state the seed stock centre and catalogue number. If plant specimens were collected from the field, describe the collection location, date and sampling procedures.* |
| Novel plant genotypes | *Describe the methods by which all novel plant genotypes were produced. This includes those generated by transgenic approaches, gene editing, chemical/radiation-based mutagenesis and hybridization. For transgenic lines, describe the transformation method, the number of independent lines analyzed and the generation upon which experiments were performed. For gene-edited lines, describe the editor used, the endogenous sequence targeted for editing, the targeting guide RNA sequence (if applicable) and how the editor was applied.* |
| Authentication | *Describe any authentication procedures for each seed stock used or novel genotype generated. Describe any experiments used to assess the effect of a mutation and, where applicable, how potential secondary effects (e.g. second site T-DNA insertions, mosiacism, off-target gene editing) were examined.* |
