# Supplementary material for: Decadal sink-source shifts of forest aboveground carbon since 1988
Source: Nat Commun. 2026 Jul 30;17:7600. doi: 10.1038/s41467-026-76093-3 (PMC13424356; doi:10.1038/s41467-026-76093-3)
Supplement: Supplementary file 3 — Transparent Peer Review file [file 41467_2026_76093_MOESM3_ESM.pdf]

nature portfolio

Peer Review File

Decadal sink-source shifts of forest aboveground carbon since 1988

Corresponding Author: Mr Zhen Qian

This file contains all reviewer reports in order by version, followed by all author rebuttals in order by version.

Version 0:

Reviewer comments:

Reviewer #1

(Remarks to the Author)
The manuscript addresses an important topic—understanding aboveground carbon (AGC) dynamics in the context of climate change. However, this study currently suffers from a number of methodological and conceptual issues that limit its scientific rigor and impact.
1. The abstract states: "In the face of ongoing climate change, sustaining global forest AGC sinks calls for large-scale afforestation and reforestation efforts…" This is a broad policy-level statement that is not directly supported or explored in the study. If included, the manuscript should provide evidence or citations linking AGC trends to afforestation/reforestation strategies.
In addition, the interactions between climate change and forest are complex. To clearly address this, the manuscript should include considerations of biophysical feedbacks and socioecological dimensions, beyond the biogeochemical processes currently emphasized.
2. Describing 0.25-degree (~25 km) resolution as "high spatial resolution" is misleading. In the context of forest monitoring and ecological heterogeneity, such resolution is generally considered coarse.
3. Why Decadal-Scale Biomass Change? The rationale for focusing on decadal-scale AGB/AGC change is not adequately justified. What is the ecological, climatological, or policy significance of tracking biomass change over decades, rather than annual or multi-year periods? The authors should explain why this temporal resolution is scientifically meaningful.
4. A critical shortcoming is the lack of robust validation for the long-term biomass maps. The manuscript presents AGC changes and trends derived from these products, but without demonstrating their accuracy or uncertainty bounds over time. It is essential to provide evidence to support the trustworthiness of these maps.
5. Signal vs. Noise: Are the Reported Changes Statistically Robust? The observed AGC changes appear to be smaller than the associated uncertainties. This raises concerns about whether the trends reported are statistically distinguishable from noise.
6. The use of static geographic variables as model predictors poses a risk of overfitting. The manuscript should demonstrate how the modeling approach accounts for or mitigates overfitting, possibly through spatial cross-validation or independent testing.
7. The dynamic predictors employed (e.g., LAI) may not be sufficiently sensitive to interannual or decadal-scale changes in AGB. The authors should critically evaluate the relevance and responsiveness of their chosen variables and consider incorporating additional or alternative indicators that are more tightly linked to biomass dynamics (e.g., tree height from LiDAR, canopy structure, etc.).

Reviewer #2

(Remarks to the Author)
The paper entitled "Decadal sink-source shifts of forest aboveground carbon since 1988" focuses on the long-term dynamics of aboveground carbon (AGC) in global forests from 1988 to 2021. It investigates the sink-source transitions of forest AGC, the role of natural and anthropogenic disturbances, and the implications for the terrestrial carbon cycle. The study uses a deep learning approach to integrate multi-source satellite observations and ground-based data, producing harmonized AGC estimates at high spatial resolution.
The paper covered a relevant topic for the forest sector and environmental monitoring. Several findings emerged from the paper, some of which could be highlighted more in the manuscript. The following are the most important findings I

discovered:
Over the period from 1988 to 2021, global forests consistently acted as a net carbon sink, sequestering an estimated 6.2 PgC. However, this global trend masks important regional differences that reflect varying disturbance regimes, land use changes, and climate dynamics.
In particular, moist tropical forests exhibited a concerning shift in their carbon balance. During the decade 2001–2010, these ecosystems transitioned from functioning as carbon sinks to becoming significant sources of carbon emissions. This reversal was largely attributed to widespread deforestation, prolonged droughts, and increased fire activity. Some recovery was observed in the subsequent decade (2011–2021), although the overall trend remains a point of concern for the stability of tropical carbon stocks.
Regionally, temperate forests made the most substantial contribution to global carbon sequestration, accounting for approximately 3.1 PgC of the total uptake. In contrast, boreal forests, which had historically played a buffering role in the carbon cycle, shifted toward being a weak carbon source in the last decade. This change is primarily due to an upsurge in natural disturbances, particularly wildfires and insect outbreaks. Meanwhile, dry tropical and subtropical forests demonstrated a more stable dynamic, with consistent aboveground carbon (AGC) gains over the study period.
A particularly noteworthy case is the Brazilian Amazon, where the nature of AGC losses has changed over time. In the earlier period (1988–2000), deforested areas accounted for around 60% of total AGC losses. By contrast, in the 2011–2021 period, this share had declined sharply to 13%. Simultaneously, carbon losses from undisturbed (intact) forest areas increased to 76%, underscoring the growing influence of climate-related disturbances, such as heatwaves and drought-induced tree mortality.
Another critical finding is the increasingly negative correlation between tropical AGC fluxes and atmospheric CO2 growth rates. Over the last decade, this relationship reached a Pearson's correlation coefficient of -0.63, suggesting that tropical forests are playing an even more pronounced role in modulating year-to-year variability in the global carbon cycle.
From a methodological perspective, the study employed a probabilistic convolutional neural network (CNN) framework, which significantly outperformed traditional estimation techniques. This approach enabled improved accuracy in both spatial and temporal estimates of AGC, representing a major advancement in forest carbon monitoring at the global scale.
Despite the significant advancements presented in this study, particularly in terms of global AGC estimation and methodological innovation, several limitations and open issues remain that warrant further consideration.
A key methodological limitation lies in the temporal assumptions underpinning the analysis. Specifically, the study assumes that the spatial relationships between predictors and aboveground carbon (AGC) remain stable over time. While this simplification may be necessary for model implementation, it may not fully capture the evolving influence of climate variability and land-use changes on forest structure and function.
Moreover, the reliability of long-term estimates is challenged by persistent gaps in observational data, especially during the earlier years of the study period. These data deficiencies could result in the underestimation of AGC stocks in certain regions, particularly where monitoring infrastructure or historical records are sparse.
Another important limitation concerns the attribution of observed AGC changes. Although the study successfully identifies major trends and regional transitions, it does not explicitly disentangle the relative contributions of different drivers, such as anthropogenic deforestation, forest degradation, or climate-induced stressors. This makes it difficult to assess the effectiveness of mitigation strategies or to anticipate future dynamics under alternative scenarios.
The study's focus on aboveground biomass, while valuable, also excludes a substantial portion of the forest carbon pool. Belowground carbon, particularly root biomass and soil organic carbon, plays a critical role in ecosystem functioning and long-term carbon storage. As such, the absence of this component limits the comprehensiveness of the carbon budget presented.
Certain aspects of forest response to climate extremes remain underexplored. While the analysis highlights the increasing role of climate-related disturbances in the Amazon, it does not systematically assess the impact of extreme climatic events (such as droughts, heatwaves, and storms) across other regions. Similarly, although El Niño–Southern Oscillation (ENSO) is acknowledged as a factor influencing AGC variability, the study does not establish a robust or quantitative relationship between ENSO phases and carbon fluxes.
From a policy perspective, the study rightly underscores the importance of afforestation, reforestation, and forest conservation. However, it stops short of translating these scientific insights into actionable policy recommendations. There is a missed opportunity to outline how these strategies could be operationalized at national or international levels, or how they might align with climate agreements such as the Paris Accord or REDD+ initiatives.
Finally, the study would benefit from a more integrated approach that considers AGC in the context of other forest carbon pools. Linking aboveground trends with changes in soil carbon or deadwood would offer a more holistic understanding of forest carbon dynamics and improve the ability to model long-term carbon sequestration potential.
In summary, the study represents a substantial advancement in our ability to monitor global aboveground carbon (AGC) dynamics with high spatial and temporal resolution. Through a robust and detailed analysis spanning over three decades, it effectively highlights key trends, including critical sink-to-source transitions and the increasing vulnerability of tropical forests to both anthropogenic and climate-related pressures. The innovative use of a probabilistic convolutional neural network contributes significantly to improving AGC estimates and sets a new benchmark for global carbon monitoring. However, despite these important contributions, several limitations remain—such as assumptions of temporal stationarity, data gaps, limited attribution of change drivers, and the exclusion of belowground carbon pools. Furthermore, the lack of a comprehensive assessment of extreme climatic events, insufficient integration of ENSO-related variability, and the absence of concrete policy guidance somewhat limit the study's broader applicability. Addressing these critical issues would not only strengthen the scientific robustness of the findings but also enhance their relevance for informing effective climate policy and sustainable forest management strategies.

General Overview:
The document is well-written and highly technical, with a clear structure and detailed explanations. However, due to its scientific nature, some sections are dense and difficult to read for non-expert audiences. There are instances of redundancy,

overly complex sentences, and inconsistent verb usage. These issues could be improved to enhance readability and flow.
Recommendations:
1. Avoid Redundancy: Eliminate repetitive phrases, especially in the abstract and results sections.
The abstract repeats the idea of "integrating multi-source satellite observations with probabilistic deep learning models" multiple times. Please, condense the abstract to avoid redundancy.
Line 581–583: "From the spatially explicit net change and the statistical summaries, we observe AGC sink-to-source shifts in tropical and boreal forests over the past three decades." This idea is repeated in other sections. Consider reducing similar sentences.
2. Improve Readability: Use simpler language where possible, especially in technical sections.
By addressing these issues, the document will be more concise, readable, and impactful. Break down long sentences into shorter

Version 1:

Reviewer comments:

Reviewer #1

(Remarks to the Author)
I would like to thank the authors for the substantial effort invested in revising the manuscript and for the thorough, well-structured, and generally convincing responses to my comments. The revised version demonstrates clear improvements in methodological transparency, validation, and discussion of limitations. The authors have addressed all major concerns raised during the review process, and the manuscript now meets the solid scientific rigor expected in remote sensing studies of this kind.While the level of methodological innovation remains consistent with current practices in the remote sensing literature, the study is well-executed and provides a valuable, consistent long-term dataset of aboveground carbon dynamics. The responses are generally appropriate, balanced, and technically sound.At this stage, only a few minor points remain that would benefit from further clarification or slight refinement before final acceptance:
1. Attribution of AGC changes. The authors appropriately clarify that a formal causal attribution is beyond the scope of the study. However, it would strengthen the manuscript to briefly outline (in the Discussion) how the presented dataset could be used in future attribution frameworks or coupled with process-based models.
2. Role of climate extremes and ENSO. The discussion on climate extremes and ENSO variability has been expanded but remains somewhat inconclusive. The lack of a strong or consistent relationship is itself a result and could be stated more explicitly. Please consider emphasizing that this finding reflects the complexity and regional heterogeneity of AGC responses, rather than limitations alone.
3. Treatment of early-period data gaps. The explanation of data gaps and the masking strategy is clear and appropriate. However, the statement that this approach “does not affect the main findings” might be slightly too strong. A more cautious wording (e.g., indicating that the approach minimizes potential bias) would improve the robustness and transparency of the interpretation.Overall, the manuscript is now scientifically sound, clearly structured, and suitable for publication after minor revisions. The remaining points are relatively small and do not require substantive methodological changes.

Version 2:

Reviewer comments:

Reviewer #2

(Remarks to the Author)
I appreciate the authors' considerable effort in revising the manuscript and their detailed, constructive responses. The revised version shows notable improvements in methodological transparency, discussion of uncertainty, and interpretation of results.

The revised manuscript offers a balanced, scientifically sound evaluation of long-term global forest AGC dynamics. The discussion is grounded in recent literature, and uncertainty is addressed throughout the paper.

Overall, I believe the authors have adequately addressed my previous concerns.

Open Access This Peer Review File is licensed under a Creative Commons Attribution 4.0 International License, which permits use, sharing, adaptation, distribution and reproduction in any medium or format, as long as you give appropriate credit to the original author(s) and the source, provide a link to the Creative Commons license, and indicate if changes were made.
In cases where reviewers are anonymous, credit should be given to 'Anonymous Referee' and the source.
The images or other third party material in this Peer Review File are included in the article’s Creative Commons license, unless indicated otherwise in a credit line to the material. If material is not included in the article’s Creative Commons license and your intended use is not permitted by statutory regulation or exceeds the permitted use, you will need to obtain permission directly from the copyright holder.
To view a copy of this license, visit https://creativecommons.org/licenses/by/4.0/

Point-by-point response to Reviewers' comments

In the following, the referee comments will be shown in blue, our responses in black. And the quoted changes we made to the manuscript main text are in *black italics.*

Reviewer #1:

General Comment: The manuscript addresses an important topic—understanding aboveground carbon (AGC) dynamics in the context of climate change. However, this study currently suffers from a number of methodological and conceptual issues that limit its scientific rigor and impact.

Response: We thank the reviewer for the constructive overall assessment and agree with the insightful comments. To address these concerns, we have substantially revised the methods and interpretation by adding stronger validation and uncertainty characterization, testing the robustness of reported changes through Signal-to-Noise Ratio analysis, tempering claims, and expanding the discussion of limitations.

Comment 1: The abstract states: "In the face of ongoing climate change, sustaining global forest AGC sinks calls for large-scale afforestation and reforestation efforts. . . " This is a broad policy-level statement that is not directly supported or explored in the study. If included, the manuscript should provide evidence or citations linking AGC trends to afforestation/reforestation strategies. In addition, the interactions between climate change and forest are complex. To clearly address this, the manuscript should include considerations of biophysical feedbacks and socioecological dimensions, beyond the biogeochemical processes currently emphasized.

Response: Thanks for pointing this out. We agree that the statement on "calls for large-scale afforestation and reforestation" was too broad, as well as that forest-climate interactions are highly complex and encompass dimensions far beyond carbon fluxes.

To address this, we have removed the overly broad policy prescription from the abstract. The revised concluding sentence of the abstract now strictly focuses on the direct scientific implications:

> *"Our findings highlight the vulnerability of carbon stocks in key biomes and provide a benchmark to track emerging sink-source shifts under anthropogenic climate change."*

Furthermore, to explicitly acknowledge the complexity of forest-climate interactions (including biophysical feedbacks and socioecological dimensions) as suggested by the reviewer, we have added a concise and targeted discussion in the final paragraph of the Discussion section (Page 7, Lines 390-397):

> *"Consequently, global carbon management frameworks, such as the Paris Agreement and Reducing Emissions from Deforestation and Forest Degradation (REDD+) initiatives, must urgently evolve to reflect these shifting drivers. Moving beyond merely curbing clear-cutting, national and international strategies should be operationalized to prioritize the protection and resilience of intact forest ecosystems against accumulating*

climate stress. While fully translating these insights into actionable policies requires integrating complex socioecological dimensions and biophysical climate feedbacks, an endeavor that falls beyond the scope of our study, we hope our multi-decadal baseline will actively facilitate these crucial future efforts."

Comment 2: Describing 0.25-degree (25 km) resolution as "high spatial resolution" is misleading. In the context of forest monitoring and ecological heterogeneity, such resolution is generally considered coarse.

Response: We agree with the reviewer. From the perspective of forest monitoring and ecological heterogeneity, 0.25° is indeed considered coarse to moderate. To avoid confusion among readers in the remote sensing and ecological communities, we have systematically removed the term "high spatial resolution" throughout the manuscript and now specify the resolution as "at 0.25°".

Comment 3: Why Decadal-Scale Biomass Change? The rationale for focusing on decadal-scale AGB/AGC change is not adequately justified. What is the ecological, climatological, or policy significance of tracking biomass change over decades, rather than annual or multi-year periods? The authors should explain why this temporal resolution is scientifically meaningful.

Response: The rationale for focusing on decadal-scale changes is to filter out short-term natural variability. Interannual biomass variability is typically driven by transient climate anomalies [1]. Consequently, evaluating year-to-year carbon sink-to-source transitions can be noisy and less indicative of fundamental ecosystem alterations. Focusing on decadal-scale shifts enables us to assess changes that are driven by climate and land use forcing and persistent disturbance regimes. An even longer time scale (e.g., via computing a trend over the entire available time period) would not resolve systematic changes over time. Furthermore, this decadal approach for evaluating global carbon budgets aligns with established community standards, such as the recent comprehensive inventory-based assessments by Pan et al. [2].

Importantly, our study does not focus exclusively on decadal scales; rather, we also adjust our temporal resolution to the specific ecological process being investigated. While we use decadal periods to robustly assess broad sink-source transitions, we use interannual variability for analyses of other critical mechanisms. For instance, we analyze the interannual contribution of different biomes to global AGC fluctuations, demonstrate the increasingly negative correlation between tropical AGC IAV and atmospheric CO_2 growth rates, and track the year-to-year dynamics of gross AGC losses in deforested versus intact forests in the Brazilian Amazon basin.

To make this rationale explicitly clear to readers, we have reinforced the justification in the Introduction (Page 1, Lines 48-50):

> *"Understanding such long-term, persistent shifts, beyond short-term natural variability driven by transient climate anomalies, helps reveal AGC dynamics associated with climate and land-use forcing and with long-term disturbance regimes."*

Furthermore, to ensure the distinction between our decadal assessments and interannual analyses is clearly delineated, we have refined the text at some transition points in the Results section.

For example:

1. When shifting focus to the biome-level contributions to global interannual variability (Pages 5, Lines 235-238):

> *"Beyond these decadal sink-source shifts, we analyze interannual variability in AGC fluxes to identify which biomes drive year-to-year fluctuations in global forest AGC. We adopt the flux partitioning approach developed by Ahlström et al. [3], which considers both the magnitude and the correlation of regional flux anomalies relative to the global signal."*

2. When analyzing the tropical AGC-CO2 coupling (Page 5, Lines 256-261):

> *"Given that our reconstruction reveals more pronounced decadal variability in tropical AGC compared to other regions (Fig. 4 and Supplementary Table 3), we now focus specifically on the dynamics of these critical ecosystems. To investigate tropical AGC dynamics and their coupling with the global carbon cycle, we analyze the correlation between interannual AGC fluxes and the atmospheric CO_2 growth rate, both linearly detrended over the 1988–2021 period."*

3. When tracking the Brazilian Amazon dynamics (Page 6, Lines 306-308, Lines 317-319):

> *"The interannual variability in AGC fluxes across tropical forests stems from a complex interplay between human activities, climatic variability, and plant physiological responses [4]."*

> *"Our partitioning analysis of the interannual variability of gross AGC losses reveals changing contributions from human- and nature-related factors over three decades (Fig. 6)."*

Comment 4: A critical shortcoming is the lack of robust validation for the long-term biomass maps. The manuscript presents AGC changes and trends derived from these products, but without demonstrating their accuracy or uncertainty bounds over time. It is essential to provide evidence to support the trustworthiness of these maps.

Response: We thank the reviewer for highlighting this critical aspect. We agree that providing evidence of trustworthiness is essential for our long-term AGC reconstruction.

Before detailing our validation framework, it is important to acknowledge a fundamental limitation inherent to all historical AGC reconstructions: there is currently no continuous, time-dependent global "ground truth" data for historical AGC [5]. Therefore, a direct evaluation of long-term, year-to-year AGB changes is, strictly speaking, not possible. To illustrate the magnitude of this challenge within the remote sensing community, we refer to the intercomparison conducted by Yang et al. [5] (in the table below). This table clearly demonstrates that existing AGC datasets that might be considered as ground truth data exhibit profound discrepancies.

Table 1 | The net total biomass changes (PgC yr^{-1}) from 2010 to 2019 globally and for five biomes (wet tropics, dry tropics, arid, temperate and boreal)

	Global	Wet tropics	Dry tropics	Arid	Temperate	Boreal
L-VOD (2010–2019)	0.50±0.20	−0.07±0.02	−0.01±0.007	−0.02±0.03	0.13±0.09	0.37±0.12
Xu et al. Ref. 6 (2010–2019)	0.56	0.05	+0.11	−0.11	+0.23	+0.17
Harris et al. Ref. 5 (2001–2019)	2.05±13.24	0.46±2.16		0.18±0.22	0.97±12.97	0.43±0.30
Liu et al. Ref. 21 (1993–2012)	0.01	−0.15	−0.17	0.15	−0.004	0.19
Pan et al. Ref. 2 (1990–2007)	1.10±0.80	−0.11±0.80		-	0.72±0.08	0.50±0.08

Comparison of regional biomass changes from this study with the recent published other products. For the estimations from L-VOD, Harris et al. and Pan et al., mean±s.d. is shown in the table.

Given that even the most established historical references exhibit pronounced differences, we cannot simply rely on a single existing product as an absolute validation standard. We instead evaluated our products through three distinct ways: first, by testing our model's predictive performance on withheld years of ESA CCI AGC data and comparing it against empirical VOD-AGC functions and machine learning methods; second, by benchmarking our AGC products against multiple independent remote-sensing AGC references to demonstrate our relative reliability; and third, by comparing our AGC stocks and changes against national inventory data.

To address the reviewer's concern, we have substantially restructured our manuscript. We completely rewrote the first subsection of the Results (now titled "Reconstruction of global forest AGC") to explicitly focus on evaluating our model's predictive performance, precision, and spatiotemporal consistency.

Specifically, we implemented three major improvements to demonstrate the accuracy of our maps over time:

1. Enhanced clarity on methodological benchmarking: To explicitly highlight the robustness of our approach, we have expanded the description of our model evaluation on withheld years of ESA CCI AGB (2010 and 2021) and our benchmarking against conventional empirical and machine learning methods. We have elevated these core results, which were already included in our original submission, from the supplement to the main text as the new Table 1. This explicitly demonstrates that our model achieves higher predictive performance and temporal consistency than alternative methods.

2. Enhanced clarity for satellite cross-validation: While the comprehensive spatiotemporal comparisons against multiple independent remote-sensing references (e.g., NASA GEDI, ESA CCI, VOD-based products, multi-source remote sensing-derived products) were already conducted in our original submission, we agree that our previous presentation was not sufficiently clear to fully convey the robustness of our AGC data. To address this, we have substantially expanded the explicit description of these validations in the main text and elevated the core benchmarking results from the supplement to the new Table 2. We now clearly articulate that while existing short-term references exhibit pronounced discrepancies among themselves, our dataset achieves a much broader and more robust alignment.

3. New inventory-based benchmarking (new Fig.1): To provide robust macro-scale validation, particularly for the historical periods lacking satellite ground-truth, we introduced a direct

comparison against the latest decadal national inventory data from Pan et al. [2]. While the aforementioned Yang et al. [5] intercomparison highlights the profound discrepancies among existing products, our analysis in Figure 1b conducts a similar but substantially more rigorous and equitable evaluation. Specifically, we strictly aligned the overlapping observational time periods across different countries and biomes to ensure a fair comparison.

We have reframed these critical validation outcomes into the revised Results (Page 3, Lines 117-155):

> *"To evaluate its predictive performance, we benchmark our model against conventional empirical VOD-to-AGC conversions and classical machine learning methods (Table 1). While VOD is a valuable biomass proxy, empirical models relying on VOD alone exhibit limited predictive capability on held-out ESA CCI test data from 2010 and 2021 ($R^2 \approx 0.2$–0.6), and their estimated time series of global annual total AGC show weak correlation with the independent 2000–2019 reference records [6] ($r = 0.35$, $p = 0.13$). In contrast, integrating multi-source data via our probabilistic CNNs improves both predictive performance ($R^2 = 0.97$) and agreement of the global total AGC time series ($r = 0.70$, $p < 0.001$). Compared with classical machine-learning models using the same multi-source predictors, linear models (Lasso and ridge) perform worse in both space and time, whereas random forest achieves comparable predictive performance ($R^2 = 0.98$) but lower temporal consistency ($r = 0.45$, $p < 0.05$)."*

> *"We further compare the reconstructed AGC maps with multiple independent remotely sensed AGC references (Supplementary Table 2 and Supplementary Note 1). Globally, our reconstructions demonstrate strong spatiotemporal agreement with the AGC references, with grid-cell-wise spatial correlations up to 0.87 and temporal correlations of regionally aggregated AGC stocks up to 0.70 (Table 2 and Supplementary Fig. 4). Although this spatial agreement decreases when evaluated within individual biomes, and temporal agreement drops for interannual AGC fluxes compared to total stocks, cross-comparisons reveal even more pronounced discrepancies among the reference datasets themselves due to differing data sources and methodologies (Supplementary Figs. 6, 7 and 8). Notably, compared to these reference products, our AGC estimates tend to correlate more strongly with each individual dataset than these datasets correlate with each other. This indicates that our new observational AGC record provides a useful, internally consistent benchmark for long-term AGC assessments."*

> *"Beyond comparisons with satellite-derived AGC references, we compare our reconstruction with national inventory data of living biomass carbon (i.e., AGC plus belowground carbon) from Pan et al. [2]. Following their biome definitions and temporal intervals (used here only for benchmarking), we find high agreement in regional carbon stocks (Fig. 1a). When evaluating net changes in carbon stocks at decadal scales (Fig. 1b), our estimates generally fall within the spread of values reported by other independent satellite-derived products for the 2000s and 2010s. Furthermore,*

across boreal and temperate regions, the temporal trajectories of our estimated carbon changes generally align with the inventory-based estimates from Pan et al. [2]. We note that the estimated magnitudes from our model, as well as those from other satellite-derived products, tend to be lower than the inventory data. This discrepancy likely arises from differences in the target variables: assessments by Pan et al. [2] consider total living biomass carbon, while ours focuses on AGC. Larger discrepancies between satellite-based estimates and inventory data occur primarily in tropical regions. One likely reason is that ground-based inventory coverage is sparse in the tropics, so inventory assessments rely more heavily on bookkeeping approaches to represent losses associated with deforestation and degradation [5]. In this context, the contrast between satellite observations and inventories underscores the value of our continuous long-term AGC record as a complement to sparse ground monitoring networks, particularly during the observationally limited 1990s."

We also reinforced this reliability in the Discussion section (Page 7, Lines 362-367):

> *"While cross-comparisons among existing short-term reference datasets reveal discrepancies, our reconstruction demonstrates generally higher correlations with these diverse datasets (Table 2 and Supplementary Note 1). The aggregated long-term AGC stocks and changes derived from our reconstruction generally align with recent forest inventory assessments [2], despite the persistent methodological disparities between continuous remote sensing observations and sparse plot networks, particularly in tropical regions (Fig. 1)."*

For the reviewer's convenience, we provide the newly added Table 1, Table 2, and Figure 1 below:

Table 1: Comparison of model performance in estimating AGC.

Method	Grid-cell-wise comparison		Global AGC time series
	R^2	RMSE ($MgC\,ha^{-1}$)	Pearson's r
VOD-only empirical models			
Arctan (CXKu-band)	0.22	70.26	0.35
Logistic (CXKu-band)	0.21	70.78	0.35
Arctan (L-band)	0.64	47.18	/
Logistic (L-band)	0.35	63.33	/
Multivariate machine learning models			
Ridge regression	0.81	34.60	0.64**
Lasso regression	0.78	37.72	0.62**
Random forest	0.98	10.44	0.45*
Our CNN	0.97	12.64	0.70***

Baseline models (Arctan [7], Logistic [8]) use only VOD, whereas machine learning models use multivariate inputs. Grid-cell-wise evaluation is against the held-out ESA CCI AGB maps (2010, 2021). Temporal performance is assessed by calculating the Pearson correlation (r) between the estimated time series of global annual total AGC and the independent reference global totals from Xu et al. [6] (2000–2019). Temporal comparison for L-band VOD is omitted due to its limited data span (available since 2010). Significance (two-tailed t-test): * $p < 0.05$, ** $p < 0.01$, *** $p < 0.001$.

Table 2: Correlation of AGB time series among observation products across global biomes during overlapping years.

Comparison	Global	Moist tropical	Dry trop. & subtrop.	Temperate	Boreal
**Pearson's r for annual AGC stock time series**					
Ours vs Liu et al. [7] (1993–2012)	-0.36	0.64**	0.25	0.70***	0.61**
Ours vs Xu et al.[6] (2000–2019)	0.70***	0.43	0.85***	0.92***	0.59**
Ours vs Boitard et al.[8] (2011–2021)	-0.01	-0.37	0.84**	0.18	0.19
Liu et al. vs Xu et al. (2000–2012)	-0.59*	0.36	0.27	0.53	0.30
Xu et al. vs Boitard et al. (2011–2019)	0.27	-0.65	0.84**	0.47	-0.18
**Pearson's r for annual AGC flux time series**					
Ours vs Liu et al. (1994–2012)	0.07	0.54*	0.47*	0.08	-0.20
Ours vs Xu et al. (2001–2019)	0.18	0.49*	0.53*	0.83***	0.20
Ours vs Boitard et al. (2012–2021)	0.27	-0.33	0.27	0.00	0.25
Liu et al. vs Xu et al. (2001–2012)	-0.23	0.12	0.23	0.26	0.06
Xu et al. vs Boitard et al. (2012–2019)	0.13	-0.60	-0.22	-0.10	-0.27

Significance (two-tailed t-test): * $p < 0.05$, ** $p < 0.01$, *** $p < 0.001$.

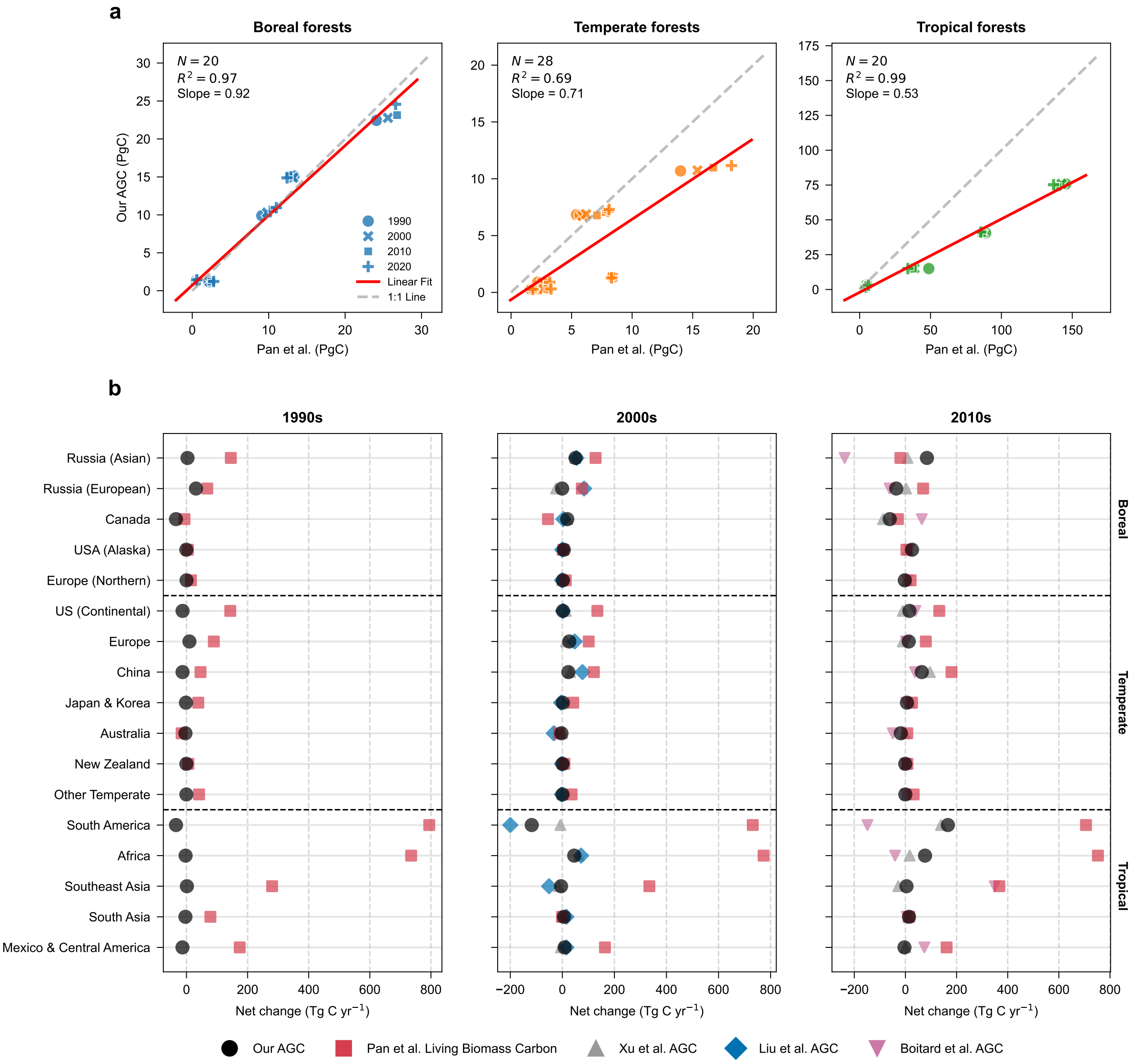

Fig. 1: Comparison of our forest AGC estimates with national inventory-based data and other remote sensing products. a, Scatter plots comparing our regional AGC stocks against the living biomass carbon stocks reported by Pan et al. [2] across boreal, temperate, and tropical forests. Each data point represents a specific geographic region or country for a given year (1990, 2000, 2010, or 2020), following the definitions in Pan et al. [2]. Solid red lines indicate the linear fits, and dashed grey lines represent the 1:1 relationships. The consistent slopes of < 1 reflect the fundamental distinction between our estimator (AGC only) and Pan et al.'s estimator (total living biomass carbon, which includes below-ground components). **b**, Comparison of decadal net carbon stock changes across regions for the periods 1990s (1990–1999), 2000s (2000–2009), and 2010s (2010–2019). Our AGC change estimates (black circles) are compared alongside inventory-based living biomass changes (red squares) by Pan et al. [2] and other available remote sensing-based AGC products (Xu et al. [6], Liu et al. [7], and Boitard et al. [8]). Regions are vertically grouped by their corresponding dominant biomes following the definitions in Pan et al. [2].

Comment 5: Signal vs. Noise: Are the Reported Changes Statistically Robust? The observed AGC changes appear to be smaller than the associated uncertainties. This raises concerns about whether the trends reported are statistically distinguishable from noise.

Response: We thank the reviewer for this insightful suggestion. We agree that demonstrating statistical robustness is essential. In fact, our original manuscript already incorporated statistical significance testing to ensure the reliability of our reported trends. For instance, in our spatially explicit trend analysis (Figure 3), we explicitly applied the modified Mann-Kendall test and exclusively presented pixels that exhibited statistically significant trends ($p < 0.05$). Furthermore, we quantified the decadal net changes and trends alongside their 95% uncertainty intervals and statistical significance tests at aggregated regional scales (detailed in Supplementary Table 3).

To build upon this robust existing foundation and explicitly prove that these changes are statistically distinguishable from underlying noise, as the reviewer suggested, we have now conducted an additional comprehensive Signal-to-Noise Ratio (SNR) analysis, providing a new reliability validation (Supplementary Figures 10 and 11, Supplementary Table 4).

Specifically, we propagated pixel-level predictive uncertainties utilizing a Monte Carlo ensemble approach. At the pixel scale, carbon fluxes naturally exhibit high-frequency variability reflecting localized environmental dynamics. Statistical theory dictates that independent random uncertainties across pixels effectively cancel out during spatial aggregation. Our new analysis quantitatively confirms this phenomenon: when aggregating pixel-level data to biome and regional scales, the SNR increases substantially, yielding highly stable signals. This explicitly validates that the macro-scale decadal sink-to-source shifts reported in our study are mostly driven by actual physical carbon dynamics. We also note that certain biomes or regions exhibit relatively low SNRs. This is because the actual net AGC changes and trends are inherently close to zero (Supplementary Table 3), causing the absolute magnitude of the 'signal' to be extremely small.

We believe that providing these spatially explicit SNR maps and regional tables equips readers with a transparent reference for interpreting the statistical confidence of our reported results across different geographic scales.

We have integrated these critical validations into the revised Results (Page 4, Lines 195-199):

> *"The reliability of these metrics is supported by Signal-to-Noise Ratio (SNR) evaluations and Monte Carlo-derived uncertainty ranges (Supplementary Figs. 10 and 11, Supplementary Tables 3 and 4). Although grid-cell-level flux estimates are inherently susceptible to high-frequency natural variability, our SNR analysis shows that spatial aggregation at regional and global scales averages out such local noise, yielding robust decadal assessments."*

We also reinforced this conclusion in the Discussion (Page 7, Lines 358-361):

> *"The spatially resolved SNR analysis underpins the reliability of our estimated AGC fluxes and trends (Supplementary Figs. 10 and 11 and Supplementary Table 4). These*

evaluations also confirm that aggregating short-term fluxes over regional and longer decadal scales substantially suppresses local noise, yielding robust signals."

For the reviewer's convenience, we provide the newly added SNR maps and the aggregated regional SNR table below:

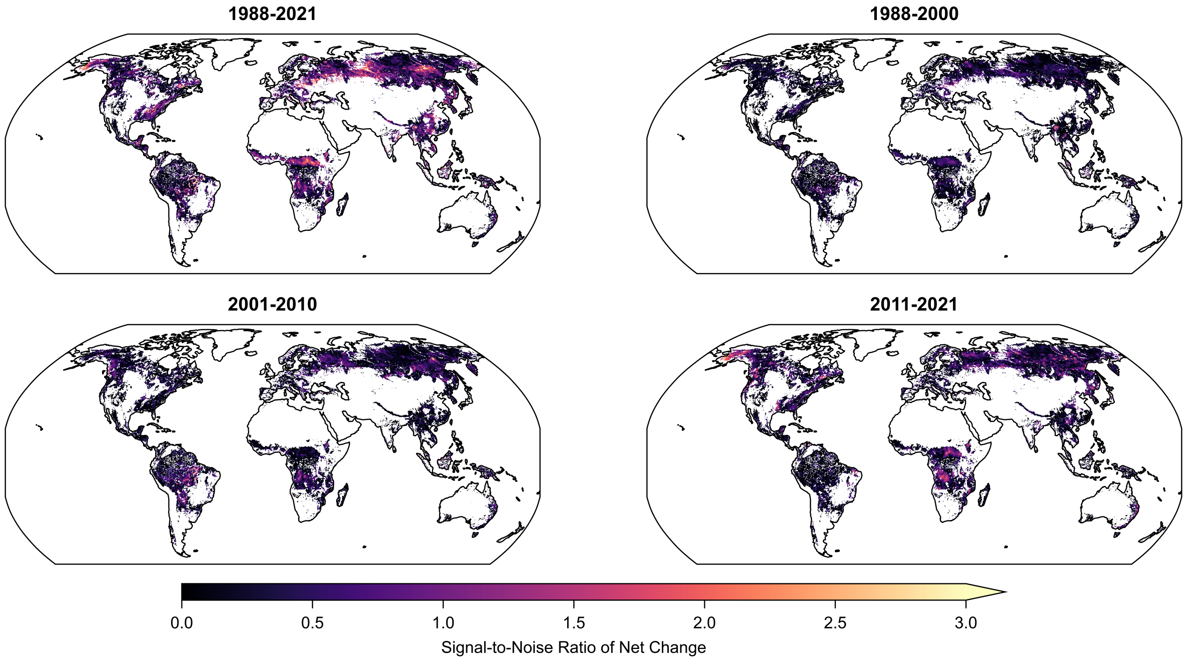

Fig. S10: Grid-cell-wise Signal-to-Noise Ratio (SNR) of forest AGC net changes. Spatially explicit SNR maps of cumulative AGC changes for the overall study period (1988–2021) and three decadal intervals (1988–2000, 2001–2010, and 2011–2021). The SNR is calculated at the 0.25° grid-cell level using a Monte Carlo ensemble approach (based on 1,000 simulations drawn from our model's predictive probability distributions). It is defined as the ratio of the absolute ensemble mean net change to the ensemble standard deviation.

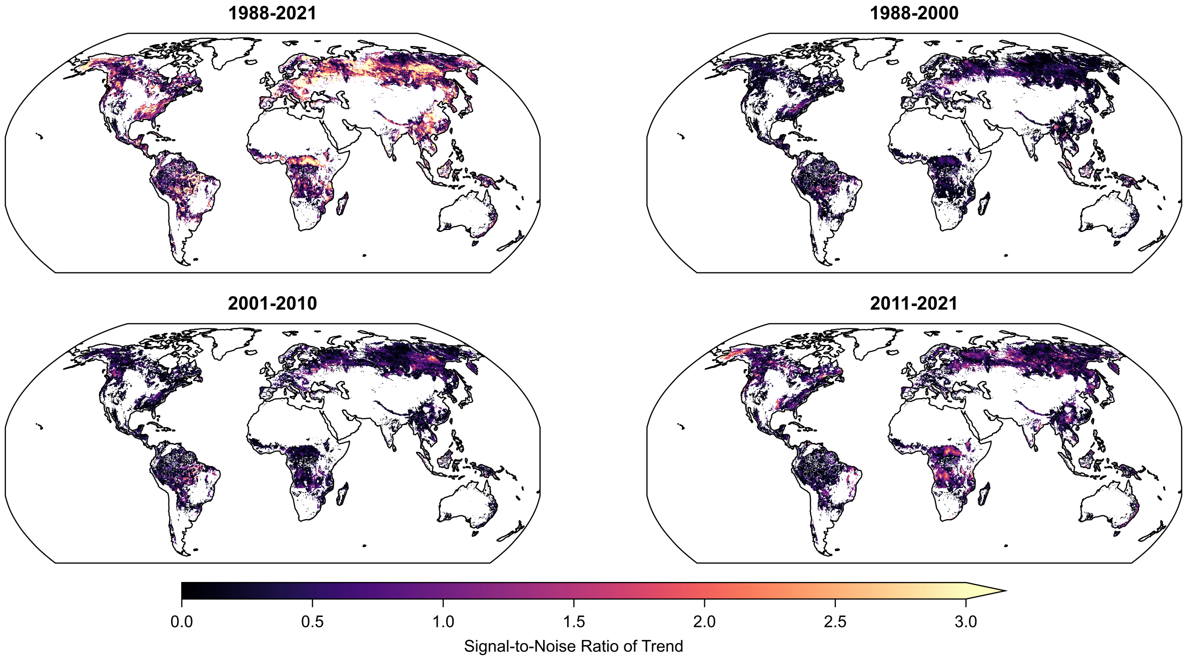

Fig. S11: Grid-cell-wise Signal-to-Noise Ratio (SNR) of forest AGC trends. Spatially explicit SNR maps of AGC trends for the overall study period (1988–2021) and three decadal intervals (1988–2000, 2001–2010, and 2011–2021). The trend SNR is calculated at the 0.25° grid-cell level utilizing a Monte Carlo ensemble approach (based on 1,000 simulations drawn from the model's predictive probability distributions). For each grid cell, the SNR is defined as the ratio of the absolute ensemble mean trend slope to the ensemble standard deviation of the slopes. Note that the years 1991 and 1992, which encompass the carbon flux anomalies caused by the Mt. Pinatubo eruption, are excluded from trend calculations.

Table S4: Signal-to-noise ratio (SNR) of decadal AGC net changes and trends across biomes, countries/regions, and tropical regions.

Category	Region	1988 – 2000		2001 – 2010		2011 – 2021		1988 – 2021	
		SNR of net change	SNR of trend	SNR of net change	SNR of trend	SNR of net change	SNR of trend	SNR of net change	SNR of trend
**Global**		24.5	6.8	4.1	8.3	18.5	24.5	63.6	83.6
**Biome**	**Moist tropical**	15.0	8.6	22.2	11.8	0.6	0.8	1.5	17.5
	**Dry tropical & subtropical**	4.4	3.3	11.2	10.1	22.3	25.6	43.3	40.6
	**Temperate**	26.9	22.2	19.3	9.5	25.4	8.4	72.5	94.5
	**Boreal**	4.8	0.0	30.3	11.2	0.8	0.0	47.0	102.0
**Country/region**	**Russia**	30.6	14.1	36.5	28.5	13.9	10.4	96.0	129.1
	**European Russia**	21.3	19.3	29.8	7.5	9.1	9.2	49.2	69.6
	**West Siberia**	24.1	13.3	2.6	6.0	25.2	19.5	67.7	63.7
	**East Siberia**	7.4	3.8	11.4	8.2	8.4	18.4	39.7	59.5
	**Far East**	7.5	1.1	27.0	16.2	14.5	2.0	45.4	80.0
	**Brazil**	3.1	3.8	30.5	8.5	0.3	7.2	11.0	27.2
	**Canada**	10.4	5.7	9.4	3.7	2.3	4.5	12.6	14.6
	**US (no Alaska)**	10.1	14.4	5.8	4.4	17.3	14.5	35.0	50.6
	**Alaska**	4.2	4.5	2.4	0.3	48.5	21.9	43.1	49.6
	**China**	2.1	2.2	1.6	9.6	18.0	15.5	35.7	54.7
	**Europe**	14.4	9.1	12.8	16.0	7.5	2.0	34.2	66.5
	**Australia**	3.5	4.6	7.8	0.8	14.4	12.8	8.2	8.4
	**DR Congo**	7.3	4.5	5.7	3.9	3.8	4.1	7.5	3.7
	**Indonesia**	1.5	4.8	4.4	5.0	2.7	0.1	6.0	14.0
	**India**	1.6	1.6	1.6	3.1	8.8	11.6	20.6	22.5
	**Peru**	0.9	0.6	7.7	5.6	0.7	4.7	1.1	0.3
**Tropics**	**Pantropical**	15.1	3.4	16.6	7.1	10.7	15.8	15.0	1.7
	**Tropical America**	8.8	0.3	31.3	9.0	1.0	8.9	4.4	22.5
	**Tropical Africa**	11.9	5.4	10.0	9.7	12.0	13.1	26.9	27.6
	**Tropical Asia**	3.5	0.6	2.6	0.8	6.8	6.8	5.6	4.7
	**Amazon Basin Rainforest**	9.1	4.3	31.7	13.8	0.3	6.1	6.3	20.8
	**Congo Basin Rainforest**	8.6	7.7	0.3	0.9	4.0	1.9	0.1	2.0
	**Indonesia Rainforest**	1.4	4.3	4.6	5.3	2.4	0.1	6.3	14.9

Note: The extent of these regions is shown in Fig. 16b, c, d, and e. The SNR values are derived using a Monte Carlo ensemble approach ($N = 1000$) to rigorously propagate grid-cell-level predictive uncertainties to the aggregated regional scales, providing robust reliability support for the estimated AGC net changes and trends. For trend calculation, we consider only grid cells with valid data across all years, and AGC stock values for 1991 and 1992 are set to no data.

6. The use of static geographic variables as model predictors poses a risk of overfitting. The manuscript should demonstrate how the modeling approach accounts for or mitigates overfitting, possibly through spatial cross-validation or independent testing.

Response: We recognize the risk of overfitting when utilizing static geographic variables. However, our methodological design, Explainable AI (XAI) feature attribution, and extensive independent validations demonstrate that our model learns generalized eco-physiological relationships rather than merely memorizing spatial locations.

We understand this concern that in a pooled training scenario (feeding the full-period data into the deep learning model), the model could easily overfit to the static geographical predictors at the expense of subtle dynamic signals. To prevent the model from assigning excessive weight to static geographic variables, we deliberately avoided pooling all years of training data into a single CNN. Instead, we trained an independent probabilistic CNN ensemble for each available reference year (2015-2020). This design forces each independent model to learn the spatial predictor-AGC relationship specific to that year's dynamic conditions without being overwhelmed by repeated static features across time. This strategy not only prevents the model from overfitting to the static artifacts of any single year, but also crucially avoids incorporating the spurious, implausible interannual fluctuations inherent in the original ESA CCI biomass products (Supplementary Fig. 2).

To explicitly prove that the CNN did not overfit to static geographic coordinates, we employed an Explainable AI (XAI) approach using integrated gradients in our original manuscript (Supplementary Fig. 3). The attribution results explicitly quantify the dynamic (time-varying) vegetation-related predictors' dominance in the decision process (56%). Static physical variables (photosynthetically active radiation, elevation, L-VOD) account for 26%. The geographic coordinates, comprising latitude and the cyclically encoded longitude (sin(lon) and cos(lon)), collectively account for less than 19%. This hierarchy confirms that the model prioritizes biophysical and physiological responses. Using coordinates in this study accounts for spatial differences in biome biomass, a methodological necessity previously demonstrated by Lang et al. [9] for monitoring global forests.

Importantly, our reconstructed AGC record demonstrates general spatiotemporal consistency with a wide array of independent reference datasets across different time periods (including NASA GEDI, VOD-based & multi-source products, and Pan et al. [2] inventory assessments). This widespread agreement with completely independent, out-of-sample temporal records serves as the most rigorous form of independent testing, verifying that our dataset is driven by true temporal dynamics rather than overfitted static spatial patterns.

Regarding the suggestion of spatial cross-validation, it is crucial to clarify the fundamental nature of our reconstruction task. Because our input data (e.g., ESA CCI, VOD, NDVI) provides complete global coverage, we are not attempting to predict unobserved locations (which is the primary purpose of spatial cross-validation). Instead, we are conducting temporal extrapolation to predict unobserved times. Therefore, standard spatial cross-validation is not directly applicable to our framework. In fact, recent research confirms that utilizing a global spatial relationship is

preferable to regional modeling for optimal AGC reconstruction [8].

We have explicitly incorporated these validations and XAI results into the rewritten Results section (Page 2, Lines 92-116):

> *"Static predictors comprise aggregated L-band VOD, photosynthetically active radiation (PAR), land surface elevation, and geographic coordinates, which help represent broad spatial and biome-specific heterogeneity [9]."*
>
> *"Using a probabilistic deep learning framework based on convolutional neural networks (CNNs) [10, 9], we model the spatial relationships between these predictors and the ESA CCI AGC reference maps [11] (see Methods; Supplementary Fig. 1). To improve training robustness, our CNN explicitly incorporates per-grid-cell uncertainty from the ESA CCI AGC products into its loss function, enabling the model to weight observations based on their confidence. Furthermore, to reduce the risk of overfitting to spatial patterns and learning spurious year-to-year fluctuations inherent in individual ESA CCI AGC snapshots (Supplementary Fig. 2), we train independent CNNs for each available reference year (2015–2020) and combine them into an ensemble. This ensemble strategy, together with uncertainty quantification techniques [12, 13], yields AGC intervals rather than only deterministic estimates. These intervals represent uncertainties arising from both the inherent noise in the satellite data (aleatoric uncertainty) and the limitations of the model itself (epistemic uncertainty). Finally, we apply the trained ensemble to reconstruct a continuous, harmonized time series of global AGC maps from 1988 to 2021."*
>
> *"We investigate the contributions of predictors to the CNNs' predictions using an Explainable AI (XAI) approach. Specifically, we aggregate feature attributions based on integrated gradients [14] across CNN ensemble members to quantify predictor importance (Supplementary Fig. 3). This analysis reveals that dynamic predictors account for approximately 56% of total attribution, indicating that time-varying vegetation signals play a major role in the reconstruction. Static biophysical and environmental variables (including PAR, elevation, and L-band VOD) contribute about 26%, leaving static encoded geographic coordinates ($<$ 19%) to serve as a complementary spatial context for these dominant eco-physiological drivers."*

7. The dynamic predictors employed (e.g., LAI) may not be sufficiently sensitive to interannual or decadal-scale changes in AGB. The authors should critically evaluate the relevance and responsiveness of their chosen variables and consider incorporating additional or alternative indicators that are more tightly linked to biomass dynamics (e.g., tree height from LiDAR, canopy structure, etc.).

Response: We agree with the reviewer that direct structural indicators, such as LiDAR-derived tree height and canopy structure, are uniquely powerful and tightly linked to AGB dynamics. However, the fundamental objective of our study is to reconstruct a continuous, long-term

AGB dynamics over more than three decades (1988–2021). Spaceborne LiDAR missions (e.g., GEDI, ICESat-2) and other direct structural datasets are relatively recent additions to the Earth observation fleet. Consequently, they cannot be employed as dynamic (time-varying) predictors required for our multi-decadal historical reconstruction.

The selection of predictors for a long-term reconstruction in our study represents a trade-off between temporal depth and direct structural measurement. To overcome the inherent absence of historical structural data, we carefully curated the most advanced and representative long-term optical and microwave proxies available since the late 1980s. The relevance and responsiveness of these variables (e.g., LAI, NDVI, and VOD) for capturing historical AGC dynamics are strongly supported by established literature:

1. While optical data do not directly measure 3D structure, the well-documented power-law scaling between LAI and tree height [15, 16] allows LAI to provide a meaningful, indirect proxy for structural changes.

2. Long-term optical indices like NDVI, which represent canopy cover, have been widely and successfully employed to track historical biomass and carbon dynamics in benchmark global studies [17, 18].

3. By explicitly combining optical data (sensitive to canopy foliage) with microwave data (e.g., VOD, sensitive to woody volume), our model leverages complementary biophysical sensitivities. This synergistic approach of integrating optical and microwave proxies has been shown to improve the estimation of long-term AGC dynamics [17, 19].

To ensure this rationale is clear to all readers, we have explicitly emphasized the biophysical relevance and historical necessity of our chosen predictors in the revised manuscript (Page 17, Lines 944-956):

> *"To reconstruct continuous multi-decadal AGB dynamics in the absence of historical 3D structural data, we rely on a carefully curated set of long-term optical and microwave proxies. As dynamic predictors, we select the recently released VODCAv2 CXKu-band VOD [20], the PKU GIMMS NDVI [21], and GIMMS LAI 4g [22], alongside tree Plant Functional Types (PFTs) and forest cover fractions derived from ESA CCI Land Cover data [23]. Crucially, these variables capture complementary biophysical properties. While optical indices like NDVI reflect canopy density and photosynthetic capacity [24], which are fundamental drivers of carbon accumulation, LAI additionally exhibits a power-law scaling relationship with structural parameters such as tree height [15, 16], serving as a useful proxy for vertical forest growth. Integrating these optical proxies with passive microwave observations (CXKu-band VOD), which are highly sensitive to woody volume and water content [7], allows our model to leverage complementary biophysical sensitivities. This combination provides a more comprehensive and historically consistent characterization of AGB changes [17], while the incorporated land-cover metrics supply essential structural constraints on spatiotemporal forest variations."*

As demonstrated by our XAI feature attribution using Integrated Gradients (Supplementary Fig. 3), these dynamic predictors are highly responsive to temporal changes: dynamic predictors account for 56% of the model's decision-making process for interannual variations, with LAI alone accounting for 17% of the total feature importance. Moreover, as detailed in our comprehensive benchmarking, the temporal comparisons demonstrate that our framework achieves higher temporal consistency and serves as a relatively more reliable baseline than existing reference datasets. These benchmarking results confirm that our chosen predictors effectively capture interannual and decadal-scale biomass dynamics.

Reviewer #2:

General Comment: The paper entitled "Decadal sink-source shifts of forest aboveground carbon since 1988" focuses on the long-term dynamics of aboveground carbon (AGC) in global forests from 1988 to 2021. It investigates the sink-source transitions of forest AGC, the role of natural and anthropogenic disturbances, and the implications for the terrestrial carbon cycle. The study uses a deep learning approach to integrate multi-source satellite observations and ground-based data, producing harmonized AGC estimates at high spatial resolution.

The paper covered a relevant topic for the forest sector and environmental monitoring. Several findings emerged from the paper, some of which could be highlighted more in the manuscript. The following are the most important findings I discovered:

Over the period from 1988 to 2021, global forests consistently acted as a net carbon sink, sequestering an estimated 6.2 PgC. However, this global trend masks important regional differences that reflect varying disturbance regimes, land use changes, and climate dynamics.

In particular, moist tropical forests exhibited a concerning shift in their carbon balance. During the decade 2001–2010, these ecosystems transitioned from functioning as carbon sinks to becoming significant sources of carbon emissions. This reversal was largely attributed to widespread deforestation, prolonged droughts, and increased fire activity. Some recovery was observed in the subsequent decade (2011–2021), although the overall trend remains a point of concern for the stability of tropical carbon stocks.

Regionally, temperate forests made the most substantial contribution to global carbon sequestration, accounting for approximately 3.1 PgC of the total uptake. In contrast, boreal forests, which had historically played a buffering role in the carbon cycle, shifted toward being a weak carbon source in the last decade. This change is primarily due to an upsurge in natural disturbances, particularly wildfires and insect outbreaks. Meanwhile, dry tropical and subtropical forests demonstrated a more stable dynamic, with consistent aboveground carbon (AGC) gains over the study period.

A particularly noteworthy case is the Brazilian Amazon, where the nature of AGC losses has changed over time. In the earlier period (1988–2000), deforested areas accounted for around 60% of total AGC losses. By contrast, in the 2011–2021 period, this share had declined sharply to 13%. Simultaneously, carbon losses from undisturbed (intact) forest areas increased to 76%, underscoring the growing influence of climate-related disturbances, such as heatwaves and drought-induced tree mortality.

Another critical finding is the increasingly negative correlation between tropical AGC fluxes and atmospheric CO2 growth rates. Over the last decade, this relationship reached a Pearson's correlation coefficient of -0.63, suggesting that tropical forests are playing an even more pronounced role in modulating year-to-year variability in the global carbon cycle.

From a methodological perspective, the study employed a probabilistic convolutional neural network (CNN) framework, which significantly outperformed traditional estimation techniques. This approach enabled improved accuracy in both spatial and temporal estimates of AGC, representing a major advancement in forest carbon monitoring at the global scale.

Despite the significant advancements presented in this study, particularly in terms of global AGC estimation and methodological innovation, several limitations and open issues remain that warrant further consideration.

Response: We are deeply grateful to the reviewer for this comprehensive and highly encouraging summary of our work. We are happy that the reviewer recognizes the substantial advancements our study brings to global forest carbon monitoring. Below, we address these constructive suggestions point by point. The manuscript was also revised to improve clarity.

Comment 1: A key methodological limitation lies in the temporal assumptions underpinning the analysis. Specifically, the study assumes that the spatial relationships between predictors and aboveground carbon (AGC) remain stable over time. While this simplification may be necessary for model implementation, it may not fully capture the evolving influence of climate variability and land-use changes on forest structure and function.

Response: We appreciate the reviewer's methodological insight and completely agree that evolving climate and land-use patterns challenge the Space-for-Time (SFT) assumption over time. However, in the absence of historical ground-truth data, relying on SFT remains a necessary and widely established standard practice for long-term AGC reconstruction (e.g., Liu et al. [7]; Fan et al. [25]; Qin et al. [26]; Yang et al. [5]; Boitard et al. [8]).

It is worth noting that our model is applied retrospectively rather than for future projection. Since the model was trained on the contemporary period (2015–2020), which encompasses a broad envelope of recent climate variability, applying it to preceding decades largely operates within the model's learned feature space. This makes our reconstruction inherently less risky than extrapolating into unprecedented future scenarios. Moreover, the estimated uncertainty intervals by our probabilistic deep learning method do not exhibit notable widening in earlier periods; this also suggests that the predictor distributions remained largely consistent and did not shift out of distribution drastically over the 34-year period. We have thoroughly revised the Discussion to transparently acknowledge this limitation (Page 8, Lines 411-422):

> *"Due to the inherent absence of a continuous, fine temporal-resolution ground truth, relying on space-for-time substitution is an unavoidable and widely established standard practice within the remote sensing community for AGC reconstructions [7, 27, 25, 26, 5, 8]. However, this approach inherently assumes that spatial predictor-AGC relationships remain stable over time, a simplification that may not fully capture the evolving impacts of climate and land-use changes. To account for this, our probabilistic framework explicitly generates predictive uncertainty bounds. These estimated uncertainty intervals do not exhibit notable widening in earlier periods,*

suggesting that the predictor distributions remained largely consistent and did not shift out of distribution drastically over the 34-year period. Moreover, because our training period captures broad recent climate variability, climate conditions over the past 30 years generally fall within this observed range, making historical reconstruction more reliable than projecting unknown future AGC dynamics."

Comment 2: Moreover, the reliability of long-term estimates is challenged by persistent gaps in observational data, especially during the earlier years of the study period. These data deficiencies could result in the underestimation of AGC stocks in certain regions, particularly where monitoring infrastructure or historical records are sparse.

Response: We thank the reviewer for the insightful comment. We agree that persistent gaps and sensor transitions in early observational data pose an inherent challenge to long-term AGC estimations, and that this could indeed lead to regional underestimations where historical records are sparse.

To mitigate this risk as much as possible, our study leveraged the most advanced, harmonized long-term datasets currently available (e.g., VODCA v2 and PKU GIMMS LAI4g/NDVI). These state-of-the-art products rely on rigorous multi-source data fusion and utilize bias-correction and spatial gap-filling algorithms designed to address the sparsity that plagued earlier satellite records. Furthermore, our modeling framework aggregates these predictors at the annual level, which helps smooth out transient data gaps such as temporary cloud cover or sensor downtime.

Despite the use of these advanced datasets, we acknowledge that spatial and temporal gaps cannot be entirely eliminated. Therefore, to ensure that these remaining data deficiencies do not compromise the reliability of our temporal analyses, we implemented a strict masking approach. As detailed in our Methods section, we explicitly exclude grid cells with missing values across the study period. While the presence of gaps might lead to a relative underestimation of the total absolute AGC stock in certain regions, this masking approach guarantees that our estimates of AGC changes, trends, and fluxes, which are the primary focus of this study, are based on consistent spatial coverage and remain robust against the influence of early data gaps.

Following the constructive feedback, we have made sure to explicitly discuss these aspects in the revised manuscript. In the Discussion (Page 7, Lines 354-356), we highlighted how the use of bias-corrected Earth observation records helps reduce structural uncertainties:

> *"The recent availability of bias-corrected, state-of-the-art Earth observation records, encompassing diverse vegetation variables and environmental data [20, 21, 22, 28, 29, 30], provides complementary information to reduce these structural uncertainties."*

We have also incorporated the precise concern regarding early satellite data gaps and potential underestimations into the limitations paragraph of the revised Discussion (Page 8, Lines 410-411):

> *"We acknowledge that persistent gaps and sensor transitions in early satellite observations could introduce regional uncertainties or potential underestimations of historical AGC stocks."*

Finally, the detailed processing of these data gaps and our justification for the masking strategy have been thoroughly elaborated in the Methods section (Page 19, Lines 1088-1101):

> *"It is also important to note that the observational data used in our modeling contain spatially and temporally heterogeneous gaps (missing data), primarily due to limitations of optical and passive microwave remote sensing, such as cloud cover, data availability, and sensor-specific constraints. Although aggregating observations over the growing season helps mitigate much of the missing-data issue, some gaps still persist, and the AGC estimates presented exhibit similar patterns of missing values. Such data gaps are common in existing AGC datasets derived from remote sensing [25, 5, 8]. To ensure the reliability of temporal analyses, we strictly exclude grid cells that have missing values at any point in the time series. This masking approach ensures that AGC change and flux estimates are based on consistent spatial coverage without the influence of artificial data gaps. However, because these masked grid cells are omitted from spatial aggregations, this approach inevitably leads to a systematic underestimation of the absolute total AGC stocks in the corresponding regions. Nevertheless, since the primary focus of this study is on AGC changes, trends, and fluxes rather than absolute stock inventories, such conservative masking does not affect our main findings."*

Comment 3: Another important limitation concerns the attribution of observed AGC changes. Although the study successfully identifies major trends and regional transitions, it does not explicitly disentangle the relative contributions of different drivers, such as anthropogenic deforestation, forest degradation, or climate-induced stressors. This makes it difficult to assess the effectiveness of mitigation strategies or to anticipate future dynamics under alternative scenarios.

Response: We thank the reviewer for highlighting the uncertainty in attributing AGB changes. While disentangling the specific drivers of AGC changes provides valuable context for mitigation strategies, performing a formal causal attribution falls outside the scope of our data-driven observational study. Our primary objective is to establish a robust, continuous baseline of long-term AGC dynamics. Quantitative attribution to isolate the relative contributions of climate-induced stressors versus specific anthropogenic drivers inherently requires process-based models and counterfactual simulations, which represent a distinct research domain.

Nevertheless, to assist readers in contextualizing our observed regional trends without overstepping into formal attribution, we have now incorporated a spatial analysis of dominant forest disturbance drivers, re-aggregated from the established global classifications by Curtis et al. [31]. By mapping the spatial fractions of deforestation, agriculture, forestry, wildfire, and urbanization, this supplementary analysis provides explicit, empirical corroboration for the regional interpretations discussed in our manuscript.

We have explicitly clarified this scope boundary, while introducing the new driver maps to assist interpretation, in the revised Discussion (Pages 7-8, Lines 405-409):

"While a formal causal attribution of AGC changes is beyond the scope of this study, we interpret observed AGC dynamics in the context of existing literature and link them to both climatic and anthropogenic influences. These region-specific interpretations regarding the drivers of carbon loss are further corroborated by spatially explicit classifications of global forest disturbance drivers [31] (Supplementary Fig. 15)."

For the reviewer's convenience, we provide the newly added disturbance driver maps below:

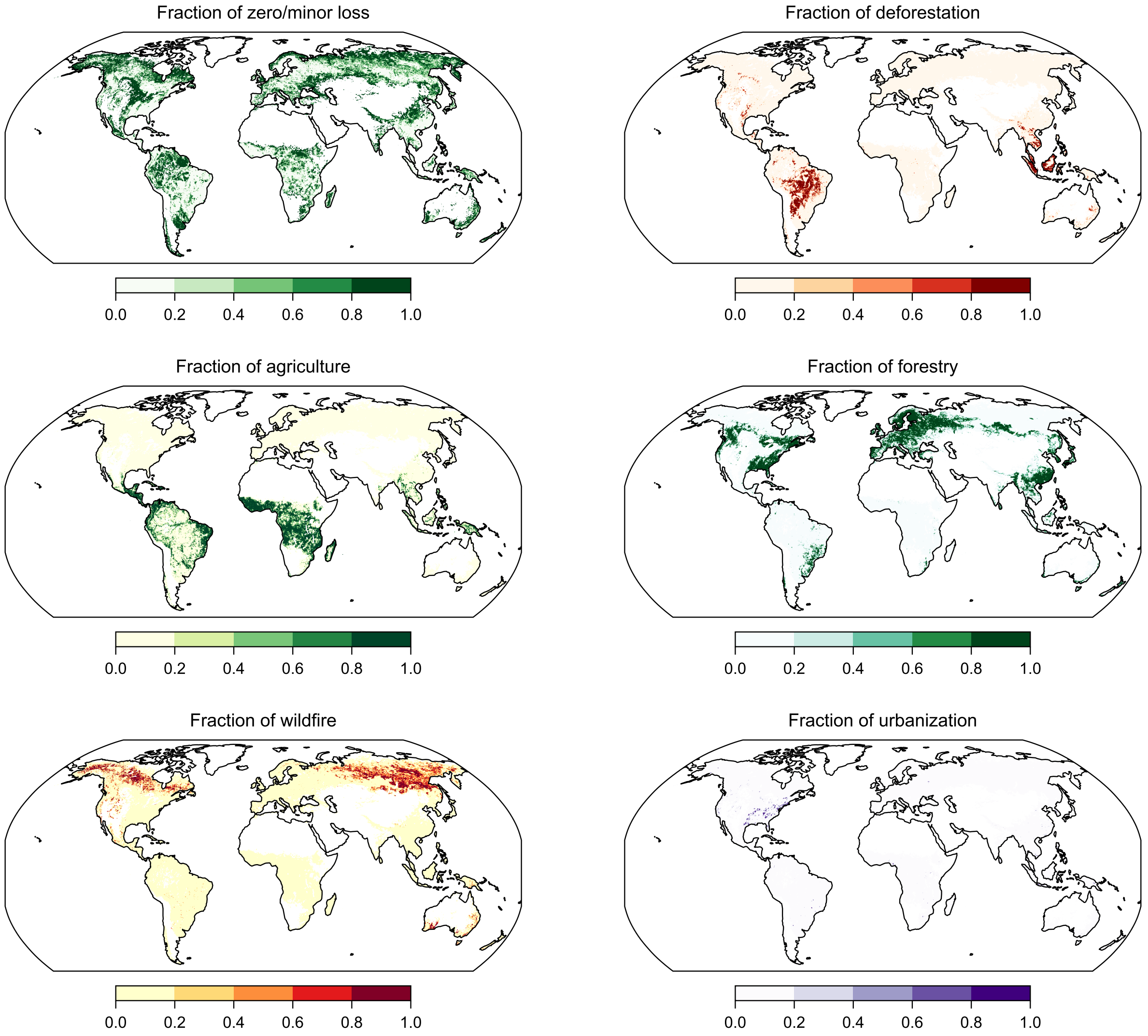

Fig. S15: Spatial distribution of global forest loss drivers at 0.25° resolution during 2001–2015. The maps display the area fraction for six distinct categories of forest cover loss: areas with zero or minor loss (i.e., regions without stand-replacing disturbance), alongside five specific forest loss drivers (commodity-driven deforestation, shifting agriculture, forestry, wildfire, and urbanization). To provide empirical context for the AGC dynamics observed in our study, the original high-resolution driver classification map from Curtis et al. [31] for the period 2001–2015 was spatially aggregated to our 0.25° grid. Grid-cell values ranging from 0.0 to 1.0 represent the areal proportion of forest loss attributed to each specific driver within the grid cell.

Comment 4: The study's focus on aboveground biomass, while valuable, also excludes a substantial portion of the forest carbon pool. Belowground carbon, particularly root biomass

and soil organic carbon, plays a critical role in ecosystem functioning and long-term carbon storage. As such, the absence of this component limits the comprehensiveness of the carbon budget presented.

Response: This is of course true. The strict focus on AGC rather than total biomass carbon is a deliberate methodological design aimed at preserving the observational reliability of our dataset.

As detailed in the Discussion section of our original manuscript, while belowground carbon represents a substantial pool, current methods for estimating belowground biomass at the global scale rely heavily on applying static, biome-specific root-to-shoot ratios or empirical functions of AGC. Integrating these highly uncertain belowground estimations would introduce large errors into our pixel-level carbon flux estimates. This is particularly problematic because the interannual variability in root-to-shoot allocation remains poorly understood, and the respective responses of above- and below-ground biomass to climate anomalies and anthropogenic disturbances can differ substantially.

By restricting our scope to AGC, we provide a more reliable, data-driven observational baseline. This approach intentionally avoids propagating unquantifiable belowground uncertainties, effectively preserving the 'degree of freedom' for end users to readily apply their preferred belowground modeling frameworks rather than enforcing highly uncertain assumptions in our product.

We have retained and further refined this explicit justification in the revised Discussion (Page 7, Lines 398-404):

> *"Several methodological limitations should be noted when interpreting our reconstructed AGC dynamics. First, our analysis specifically focuses on global forest AGC, not total biomass carbon. Total biomass carbon, including both above- and below-ground components, is commonly estimated using biome-specific root-to-shoot ratios [6, 5] or empirical functions [32]. These approaches introduce additional uncertainty into carbon flux estimates, particularly since the interannual variability in the relationship between AGB and below-ground biomass remains poorly understood, and responses of AGB and below-ground biomass to climate and anthropogenic disturbances can also differ [33, 34]."*

Comment 5: Certain aspects of forest response to climate extremes remain underexplored. While the analysis highlights the increasing role of climate-related disturbances in the Amazon, it does not systematically assess the impact of extreme climatic events (such as droughts, heatwaves, and storms) across other regions. Similarly, although El Niño–Southern Oscillation (ENSO) is acknowledged as a factor influencing AGC variability, the study does not establish a robust or quantitative relationship between ENSO phases and carbon fluxes.

Response: Thank you for this comment. While assessing the individual impacts of all localized extreme events (such as regional heatwaves or specific storms) globally provides valuable ecological insights, systematically attributing these high-frequency, localized extremes falls well outside the scope of our multi-decadal data reconstruction. We will make the dataset public to provide the broader scientific community with a more reliable historical baseline. This will facilitate

follow-up research to systematically evaluate the roles of various localized climatic disturbances and their interactions with AGC dynamics.

In fact, we did investigate the impact of droughts in pan-tropical forests using the Standardised Precipitation Evapotranspiration Index (SPEI) in the original third subsection of Results. While our dataset explicitly captures large AGC losses during well-documented droughts in the Amazon (e.g., 2004/05, 2009/10, and 2015/16), the signal is much less clear when the analysis is expanded to the Congo Basin and Southeast Asia (Indonesia). In these other regions, the overall correlation between SPEI-derived drought areas and AGC dynamics is not clear. As shown in Fig. 5, we observed instances in the Congo and Indonesia where high AGC loss occurred in the absence of severe drought.

This discrepancy highlights the highly complex and regionally heterogeneous nature of forest carbon dynamics. It suggests that analyzing a single climatic index, such as drought, cannot fully explain AGC losses. In regions like Indonesia and the Congo, other factors, such as widespread human disturbances (e.g., land-use changes, deforestation) and other localized extreme events, likely play dominant roles that confound the drought signal.

We agree that this analysis requires further clarification. Accordingly, we have revised the text to explicitly highlight this finding (Pages 6, Lines 292-303):

> *"To examine such dynamics in our dataset, we compare regional AGC dynamics with moisture availability conditions quantified as the fraction of grid cells with three-month Standardised Precipitation Evapotranspiration Index (SPEI3) ≤ -1 [35] (see background colouring in Fig. 5c-i). While localized AGC losses co-occur with severe moisture stress during specific events, the broader regional relationship is often less distinct. For example, the Congo and Indonesia rainforests occasionally exhibit sharp declines in AGC without corresponding peaks in drought fraction (Fig. 5g,i). Such mismatches likely reflect confounding influences, including concurrent anthropogenic disturbances and other climate extremes, that also shape AGC dynamics. However, for the Amazon, we specifically examine four well-documented large-scale drought events (1997/98 [36], 2004/05 [37], 2009/10 [38], and 2015/16 [39]) and indeed observe pronounced minima in Amazon AGC stock during the latter three drought years (Fig. 5e). This alignment suggests that climate extremes are driving substantial carbon losses in the Amazon."*

The drought analysis is further highlighted in the Supplementary Discussion:

> *"Moreover, our results do not show a clear impact of environmental conditions, such as well-documented droughts, on the interannual tropical AGC dynamics, except to some extent in the Amazon. At the grid-cell level, the relationship between AGC flux and the SPEI is generally weak, with a median correlation coefficient of 0.09 across the pan-tropical forests (Supplementary Fig. 19). However, stronger positive correlations are observed in dry tropical and subtropical forests, likely due to their higher sensitivity*

to water availability [3, 40]. The overall weak drought fingerprint observed in AGC dynamics may result from several factors: First, interannual variability and long-term stock trajectories in tropical AGC differ across datasets (Supplementary Fig. 14 and Table. 5). Hence, the precision of AGC estimates may still not be high enough to observe drought responses sufficiently well, owing to ongoing uncertainties in existing datasets. In particular, recent studies have shown that purely VOD-derived AGC estimates may be influenced by changes in water stress, which could overstate biomass fluctuations during water scarcity periods [41, 42]. Second, AGC changes are influenced by environmental conditions in complex ways, and affected by multiple local disturbance factors, and by tree regrowth and reforestation. For example, drought is associated with lower cloud coverage and hence higher downwelling short-wave radiation, which may in some cases even increase gross primary productivity and hence AGC [43]. Consequently, further research is needed to clarify the extent to which drought events influence AGC storage."

Regarding ENSO and large-scale water stress, we agree with the reviewer on the critical importance of these climatic drivers. Although our dataset captured severe AGC losses during the extreme 2015/16 El Niño event, our overall analysis did not reveal a pervasive, robust relationship between interannual AGC fluxes and ENSO phases at the pan-tropical scale (Supplementary Fig.17). This low correlation suggests the mechanistic relationship between ENSO and pantropical forest dynamics is highly complex and spatially heterogeneous. Additionally, a likely reason is that limitations in current AGC products may introduce inherent uncertainties; the precision of AGC datasets may not sufficiently capture ENSO impacts on AGC.

We have ensured this critical scientific nuance remains prominently highlighted in our revised Discussion (Page 7, Lines 374-382):

"Crucially, our analysis indicates a strengthening negative coupling between tropical AGC fluxes and the atmospheric CO_2 growth rate over the past three decades. Building on prior hypotheses that attributed the intensifying water-carbon coupling primarily to ENSO-induced water stress [42], we investigate these climatic drivers across our 34-year continuous record (see Supplementary Discussion). While our dataset captures the severe AGC losses associated with the extreme 2015/16 El Niño event, we found insufficient evidence to attribute the overall large-scale interannual carbon anomalies to ENSO phases (Supplementary Fig. 17). The absence of a widespread correlation may partly indicate the inherent uncertainties in AGC reconstructions, which may hinder the full detection of ENSO impacts on AGC dynamics."

We have put an original, detailed discussion into the Supplementary Discussion:

"Interestingly, our analysis reveals an intensifying negative correlation between annual tropical AGC fluxes and atmospheric CO_2 growth rate (Supplementary Fig. 13). This highlights the growing importance of tropical AGC dynamics in shaping the terrestrial

carbon cycle variability. Our results are consistent with an earlier finding showing a strong association between tropical AGC fluxes and CO_2 growth rates during 2011–2017 [25]. Moreover, our results also provide independent support for previous exploratory evidence of a strengthening negative AGC–CO_2 correlation in tropics from 1989–2003 to 2002–2016 [42]. While the authors acknowledged uncertainties in their VOD-derived AGC estimates, particularly the sensitivity of VOD signals to soil moisture [41], our multivariable reconstruction approach helps mitigate these confounding effects. Moreover, our newly developed AGC estimates offer continuous decadal-scale evidence from 1989 to 2021, extending the previously observed relationship. This earlier study [42] further interpreted the intensifying negative AGC-CO_2 correlation as a reflection of strengthened water–carbon coupling in the tropics, likely associated with ENSO teleconnections, based primarily on a comparison between 1960–1989 and 1989–2018. However, our decadal-scale analysis from 1988–2021 does not reveal a consistent relationship between ENSO phases and AGC dynamics (Supplementary Fig.17), nor a clear pattern of intensifying negative water-carbon coupling (Supplementary Fig. 18a). Both the direct correlation between water availability and AGC flux, and the partial correlation after accounting for temperature effects, also show no clear strengthening trend over the 1989–2021 period (Supplementary Fig.18b,c). Notably, the decade 2001–2010 exhibits a marked shift in the coupling structures between water availability and both atmospheric CO_2 growth rate and AGC flux. This anomaly in 2001–2010 may be related to more moderate but frequent climatic fluctuations, as well as accelerating deforestation [44]. Our findings suggest that attributing the intensifying negative AGC-CO_2 correlation solely to ENSO-induced water stress is likely insufficient and oversimplified. In addition, improvements in observational data quality over time, such as changes in satellite retrieval algorithms or sensor characteristics, may have introduced additional sources of uncertainty into the observed AGC-CO_2 coupling. These considerations underscore the need for further investigation to disentangle the relative contributions of climatic and anthropogenic drivers."

Comment 6: From a policy perspective, the study rightly underscores the importance of afforestation, reforestation, and forest conservation. However, it stops short of translating these scientific insights into actionable policy recommendations. There is a missed opportunity to outline how these strategies could be operationalized at national or international levels, or how they might align with climate agreements such as the Paris Accord or REDD+ initiatives.

Response: We appreciate the reviewer's perspective on the importance of translating our scientific insights into real-world policy frameworks. We agree that aligning our findings with global frameworks like the Paris Agreement and REDD+ adds significant practical value. However, Reviewer 1 cautioned against overextending our analysis into the highly complex realm of policy recommendations, noting that drafting actionable policies requires integrating intricate socio-economic and political dimensions that fall substantially outside the data-driven, observational scope of our study.

To address both this valuable recommendation and Reviewer 1's valid concerns, we have revised

the Discussion to specifically incorporate the suggested frameworks (the Paris Agreement and REDD+) and emphasize the necessity of their operationalization.

We have updated the Discussion (Page 7, Lines 390-397) to reflect this balanced perspective:

> *"Consequently, global carbon management frameworks, such as the Paris Agreement and Reducing Emissions from Deforestation and Forest Degradation (REDD+) initiatives, must urgently evolve to reflect these shifting drivers. Moving beyond merely curbing clear-cutting, national and international strategies should be operationalized to prioritize the protection and resilience of intact forest ecosystems against accumulating climate stress. While fully translating these insights into actionable policies requires integrating complex socioecological dimensions and biophysical climate feedbacks, an endeavor that falls beyond the scope of our study, we hope our multi-decadal baseline will actively facilitate these crucial future efforts."*

Comment 7: Finally, the study would benefit from a more integrated approach that considers AGC in the context of other forest carbon pools. Linking aboveground trends with changes in soil carbon or deadwood would offer a more holistic understanding of forest carbon dynamics and improve the ability to model long-term carbon sequestration potential.

In summary, the study represents a substantial advancement in our ability to monitor global aboveground carbon (AGC) dynamics with high spatial and temporal resolution. Through a robust and detailed analysis spanning over three decades, it effectively highlights key trends, including critical sink-to-source transitions and the increasing vulnerability of tropical forests to both anthropogenic and climate-related pressures. The innovative use of a probabilistic convolutional neural network contributes significantly to improving AGC estimates and sets a new benchmark for global carbon monitoring. However, despite these important contributions, several limitations remain—such as assumptions of temporal stationarity, data gaps, limited attribution of change drivers, and the exclusion of belowground carbon pools. Furthermore, the lack of a comprehensive assessment of extreme climatic events, insufficient integration of ENSO-related variability, and the absence of concrete policy guidance somewhat limit the study's broader applicability. Addressing these critical issues would not only strengthen the scientific robustness of the findings but also enhance their relevance for informing effective climate policy and sustainable forest management strategies.

Response: We thank the reviewer for this adequate summary, recognizing our reconstruction as a "substantial advancement" and a "new benchmark for global carbon monitoring."

Regarding the integration of other forest carbon pools (e.g., soil carbon and deadwood), we agree that achieving a holistic, multi-pool carbon budget is the ultimate goal for the Earth system science community. However, as detailed in our response to Comment 4, maintaining a strict focus on AGC in this specific study deliberately prevents the introduction of the massive uncertainties associated with modeling those unobserved pools, thereby preserving the strict observational fidelity of our dataset. We hope our AGC product will serve as a reliable foundation for future studies to couple with advanced soil and deadwood models.

For all the remaining limitations summarized in this conclusion, we have addressed each concern point-by-point in the preceding responses. We have extensively expanded our revised Discussion and Supplementary Information to transparently acknowledge these limitations, provide new empirical validations, and refine our scientific interpretations without overstepping the boundaries of our observational framework.

General Overview & Recommendations: The document is well-written and highly technical, with a clear structure and detailed explanations. However, due to its scientific nature, some sections are dense and difficult to read for non-expert audiences. There are instances of redundancy, overly complex sentences, and inconsistent verb usage. These issues could be improved to enhance readability and flow.

1. Avoid Redundancy: Eliminate repetitive phrases, especially in the abstract and results sections.

The abstract repeats the idea of "integrating multi-source satellite observations with probabilistic deep learning models" multiple times. Please, condense the abstract to avoid redundancy.

Line 581–583: "From the spatially explicit net change and the statistical summaries, we observe AGC sink-to-source shifts in tropical and boreal forests over the past three decades." This idea is repeated in other sections. Consider reducing similar sentences.

2. Improve Readability: Use simpler language where possible, especially in technical sections. By addressing these issues, the document will be more concise, readable, and impactful. Break down long sentences into shorter.

Response: We sincerely appreciate the careful reading of our manuscript. We have performed a comprehensive revision of the text. Specifically, we have:

1. Condensed the Abstract to eliminate redundant statements.

2. Removed repetitive summary sentences in the Results and Discussion sections.

3. Systematically clarify overly complex long sentences throughout the main text.

References and Notes

[1] Piao, S. et al. Interannual variation of terrestrial carbon cycle: Issues and perspectives. Global Change Biology **26**, 300–318 (2020).

[2] Pan, Y. et al. The enduring world forest carbon sink. Nature **631**, 563–569 (2024).

[3] Ahlström, A. et al. The dominant role of semi-arid ecosystems in the trend and variability of the land CO_2 sink. Science **348**, 895–899 (2015).

[4] Gatti, L. V. et al. Amazonia as a carbon source linked to deforestation and climate change. Nature **595**, 388–393 (2021).

[5] Yang, H. et al. Global increase in biomass carbon stock dominated by growth of northern young forests over past decade. Nature Geoscience **16**, 886–892 (2023).

[6] Xu, L. et al. Changes in global terrestrial live biomass over the 21st century. Science Advances **7**, eabe9829 (2021).

[7] Liu, Y. Y. et al. Recent reversal in loss of global terrestrial biomass. Nature Climate Change **5**, 470–474 (2015).

[8] Boitard, S. et al. Aboveground biomass dataset from SMOS L-band vegetation optical depth and reference maps. Earth System Science Data **17**, 1101–1119 (2025).

[9] Lang, N., Jetz, W., Schindler, K. & Wegner, J. D. A high-resolution canopy height model of the Earth. Nature Ecology & Evolution **7**, 1778–1789 (2023).

[10] Lang, N., Schindler, K. & Wegner, J. D. Country-wide high-resolution vegetation height mapping with Sentinel-2. Remote Sensing of Environment **233**, 111347 (2019).

[11] Santoro, M. et al. Design and performance of the Climate Change Initiative Biomass global retrieval algorithm. Science of Remote Sensing **10**, 100169 (2024).

[12] Kendall, A. & Gal, Y. What uncertainties do we need in bayesian deep learning for computer vision? In Guyon, I. et al. (eds.) Advances in Neural Information Processing Systems, vol. 30 (Curran Associates, Inc., 2017).

[13] Lakshminarayanan, B., Pritzel, A. & Blundell, C. Simple and scalable predictive uncertainty estimation using deep ensembles. In Guyon, I. et al. (eds.) Advances in Neural Information Processing Systems, vol. 30 (Curran Associates, Inc., 2017).

[14] Sundararajan, M., Taly, A. & Yan, Q. Axiomatic attribution for deep networks. In Precup, D. & Teh, Y. W. (eds.) International conference on machine learning, 3319–3328. PMLR (PMLR, Sydney, Australia, 2017).

[15] West, G. B., Brown, J. H. & Enquist, B. J. A general model for the structure and allometry of plant vascular systems. Nature **400**, 664–667 (1999).

[16] Zhang, G. et al. Estimation of forest aboveground biomass in california using canopy height and leaf area index estimated from satellite data. Remote Sensing of Environment **151**, 44–56 (2014).

[17] Liu, Y. Y., De Jeu, R. A., McCabe, M. F., Evans, J. P. & Van Dijk, A. I. Global long-term passive microwave satellite-based retrievals of vegetation optical depth. Geophysical Research Letters **38** (2011).

[18] Harris, N. L. et al. Global maps of twenty-first century forest carbon fluxes. Nature Climate Change **11**, 234–240 (2021).

[19] Liu, Y. Y., Van Dijk, A. I. J. M., McCabe, M. F., Evans, J. P. & De Jeu, R. A. M. Global vegetation biomass change (1988-2008) and attribution to environmental and human drivers: Global vegetation biomass change. Global Ecology and Biogeography **22**, 692–705 (2013).

[20] Zotta, R.-M. et al. VODCA v2: Multi-sensor, multi-frequency vegetation optical depth data for long-term canopy dynamics and biomass monitoring. Earth System Science Data **16**, 4573–4617 (2024).

[21] Li, M. et al. Spatiotemporally consistent global dataset of the GIMMS Normalized Difference Vegetation Index (PKU GIMMS NDVI) from 1982 to 2022. Earth System Science Data **15**, 4181–4203 (2023).

[22] Cao, S. et al. Spatiotemporally consistent global dataset of the GIMMS leaf area index (GIMMS LAI4g) from 1982 to 2020. Earth System Science Data **15**, 4877–4899 (2023).

[23] CCI, E. L. C. Land Cover CCI Product user guide version 2.0. UCL-Geomatics: London, UK (2017).

[24] Gamon, J. A. et al. Relationships between ndvi, canopy structure, and photosynthesis in three californian vegetation types. Ecological applications **5**, 28–41 (1995).

[25] Fan, L. et al. Satellite-observed pantropical carbon dynamics. Nature Plants **5**, 944–951 (2019).

[26] Qin, Y. et al. Carbon loss from forest degradation exceeds that from deforestation in the Brazilian Amazon. Nature Climate Change **11**, 442–448 (2021).

[27] Brandt, M. et al. Satellite passive microwaves reveal recent climate-induced carbon losses in African drylands. Nature Ecology & Evolution **2**, 827–835 (2018).

[28] Harper, K. L. et al. A 29-year time series of annual 300 m resolution plant-functional-type maps for climate models. Earth System Science Data **15**, 1465–1499 (2023).

[29] Wang, D. et al. A New Set of MODIS Land Products (MCD18): Downward Shortwave Radiation and Photosynthetically Active Radiation. Remote Sensing **12**, 168 (2020).

[30] MacFerrin, M., Amante, C., Carignan, K., Love, M. & Lim, E. The earth topography 2022 (ETOPO 2022) global DEM dataset. Earth System Science Data Discussions **2024**, 1–24 (2024).

[31] Curtis, P. G., Slay, C. M., Harris, N. L., Tyukavina, A. & Hansen, M. C. Classifying drivers of global forest loss. Science **361**, 1108–1111 (2018).

[32] Feng, Y. et al. Doubling of annual forest carbon loss over the tropics during the early twenty-first century. Nature Sustainability **5**, 444–451 (2022).

[33] Zhang, B., Zhou, X., Zhou, L. & Ju, R. A global synthesis of below-ground carbon responses to biotic disturbance: A meta-analysis. Global Ecology and Biogeography **24**, 126–138 (2015).

[34] Spawn, S. A., Sullivan, C. C., Lark, T. J. & Gibbs, H. K. Harmonized global maps of above and belowground biomass carbon density in the year 2010. Scientific Data **7**, 112 (2020).

[35] Tirivarombo, S., Osupile, D. & Eliasson, P. Drought monitoring and analysis: Standardised Precipitation Evapotranspiration Index (SPEI) and Standardised Precipitation Index (SPI). Physics and Chemistry of the Earth, Parts A/B/C **106**, 1–10 (2018).

[36] Siegert, F., Ruecker, G., Hinrichs, A. & Hoffmann, A. A. Increased damage from fires in logged forests during droughts caused by El Niño. Nature **414**, 437–440 (2001).

[37] Aragão, L. E. O. et al. Spatial patterns and fire response of recent Amazonian droughts. Geophysical Research Letters **34** (2007).

[38] Lewis, S. L., Brando, P. M., Phillips, O. L., Van Der Heijden, G. M. F. & Nepstad, D. The 2010 Amazon Drought. Science **331**, 554–554 (2011).

[39] Bennett, A. C. et al. Sensitivity of South American tropical forests to an extreme climate anomaly. Nature Climate Change **13**, 967–974 (2023).

[40] Poulter, B. et al. Contribution of semi-arid ecosystems to interannual variability of the global carbon cycle. Nature **509**, 600–603 (2014).

[41] Konings, A. G., Holtzman, N. M., Rao, K., Xu, L. & Saatchi, S. S. Interannual Variations of Vegetation Optical Depth are Due to Both Water Stress and Biomass Changes. Geophysical Research Letters **48**, e2021GL095267 (2021).

[42] Liu, L. et al. Increasingly negative tropical water–interannual CO2 growth rate coupling. Nature **618**, 755–760 (2023).

[43] Jones, M. O., Kimball, J. S. & Nemani, R. R. Asynchronous Amazon forest canopy phenology indicates adaptation to both water and light availability. Environmental Research Letters **9**, 124021 (2014).

[44] Malhi, Y. et al. Climate Change, Deforestation, and the Fate of the Amazon. Science **319**, 169–172 (2008).

Point-by-point response to Reviewers' comments

In the following, the referee comments will be shown in blue, our responses in black. And the quoted changes we made to the manuscript main text are in *black italics*.

Reviewer #1:

General Comment: I would like to thank the authors for the substantial effort invested in revising the manuscript and for the thorough, well-structured, and generally convincing responses to my comments. The revised version demonstrates clear improvements in methodological transparency, validation, and discussion of limitations. The authors have addressed all major concerns raised during the review process, and the manuscript now meets the solid scientific rigor expected in remote sensing studies of this kind. While the level of methodological innovation remains consistent with current practices in the remote sensing literature, the study is well-executed and provides a valuable, consistent long-term dataset of aboveground carbon dynamics. The responses are generally appropriate, balanced, and technically sound. At this stage, only a few minor points remain that would benefit from further clarification or slight refinement before final acceptance.

Response: We sincerely appreciate the constructive feedback provided throughout the review process, which has significantly improved the methodological transparency and overall scientific rigor of our work. We have carefully addressed the remaining minor points below, with all modifications highlighted in the revised manuscript text.

Comment 1: Attribution of AGC changes. The authors appropriately clarify that a formal causal attribution is beyond the scope of the study. However, it would strengthen the manuscript to briefly outline (in the Discussion) how the presented dataset could be used in future attribution frameworks or coupled with process-based models.

Response: We agree with the reviewer's constructive suggestion. To address this, we have expanded the final paragraph of the Discussion section to explicitly outline the potential applications of our reconstruction in future climate attribution frameworks and process-based modeling efforts. Specifically, we have added the following text to the revised manuscript (Page 8, Lines 428-435):

> *"Crucially, our multi-decadal reconstruction serves as a robust observational benchmark to evaluate and calibrate process-based vegetation models and constrain their uncertainties. Once calibrated by, or evaluated against, our long-term AGC dataset, these models can then be effectively employed in attribution studies to disentangle the specific impacts of anthropogenic radiative forcing, direct land-use change, and natural variability. Furthermore, empirically identified phenomena in our product, such as the strengthening negative correlation between tropical AGC fluxes and the growth rate of CO_2, provide critical targets for evaluating and enhancing the transient responses of existing models."*

Comment 2: Role of climate extremes and ENSO. The discussion on climate extremes and ENSO variability has been expanded but remains somewhat inconclusive. The lack of a strong or consistent relationship is itself a result and could be stated more explicitly. Please consider emphasizing that this finding reflects the complexity and regional heterogeneity of AGC responses, rather than limitations alone.

Response: We agree with the reviewer's observation. We have revised the Discussion section to explicitly acknowledge that inherent observational limitations may obscure macro-scale climatic signals, while also noting that the lack of a consistent signal highlights the complexity and regional heterogeneity of forest carbon dynamics.

We have modified the relevant paragraph in the Discussion to read as follows (Page 7, Lines 381-385):

> *"The absence of a widespread correlation may partly indicate the inherent uncertainties in AGC reconstructions, which may hinder the full detection of ENSO impacts on AGC dynamics. At the same time, AGC dynamics reflect the combined effects of multiple interacting biogeochemical processes and anthropogenic disturbances. These complex and spatially heterogeneous controls might obscure the potential mechanistic influence of ENSO."*

Comment 3: Treatment of early-period data gaps. The explanation of data gaps and the masking strategy is clear and appropriate. However, the statement that this approach "does not affect the main findings" might be slightly too strong. A more cautious wording (e.g., indicating that the approach minimizes potential bias) would improve the robustness and transparency of the interpretation.

Response: We agree with the reviewer that our previous phrasing was too definitive. To improve the transparency and robustness of our interpretation, we have modified the relevant sentence in the Methods section (Page 19, Lines 1099-1102):

> *"Nevertheless, because the primary focus of this study is on long-term AGC changes, trends, and fluxes rather than absolute stock inventories, this conservative masking strategy is expected to minimize potential biases arising from inconsistent spatial coverage and is unlikely to materially alter our main findings."*

General Overview: Overall, the manuscript is now scientifically sound, clearly structured, and suitable for publication after minor revisions. The remaining points are relatively small and do not require substantive methodological changes.

Response: We acknowledge the reviewer's concluding assessment. As detailed in the responses above, we have addressed all the minor points and incorporated the necessary refinements into the revised manuscript. We thank the reviewer for their time and contribution to the peer-review process.
